# Prevalence of anemia in India: a systematic review, meta-analysis and geospatial analysis

**DOI:** 10.1186/s12889-025-22439-3

**Published:** 2025-04-04

**Authors:** Jyothika Jeevan, Kalesh M. Karun, Amitha Puranik, C. Deepa, Lintu MK, Manish Barvaliya

**Affiliations:** 1 Department of Health Systems Research, ICMR - National Institute of Traditional Medicine, Nehru Nagar, Belagavi, Karnataka 590010 India; 2 Department of Biostatistics, St. Thomas College, Palai, Kerala 686574 India; 3 Department of Cardiovascular Sciences, College of Life Sciences, University of Leicester, Leicester, LE1 7RH UK; 4 Department of Data Science, Prasanna School of Public Health, Manipal Academy of Higher Education, Manipal, Karnataka 576104 India

**Keywords:** Anemia, Geospatial analysis, India, Meta-analysis, Prevalence, Systematic review

## Abstract

**Background:**

Anemia is a major health concern in India, ranking second in maternal mortality and exhibits a higher prevalence compared to many other developing nations. This study aims to analyze prevalence of anemia across age groups using systematic review and meta-analysis.

**Methods:**

The present systematic review and meta-analysis includes cross-sectional studies from 1995 to 2023, reporting prevalence of anemia in India. Two authors independently screened and extracted data from relevant articles sourced from PubMed, Scopus, and Web of Science. Study quality was assessed using the Newcastle–Ottawa Scale and model selection was based on observed heterogeneity (I^2^). Geospatial analysis and cumulative meta-analysis were performed using R 4.3.3 and STATA 16 software.

**Results:**

Across 157 studies, the prevalence of anemia varied among different age groups and regions in India. Toddlers (under 3 years) had a 69% prevalence, with highest in the Eastern (87%) and lowest in the Northern (50%) regions. Pre-school children (3–5 years) had a 64% prevalence, exhibiting 85% and 37% in the Central and the North-Eastern regions, respectively.

Among the school going children, the overall prevalence was 51.2%. The highest prevalence at 83.9% was seen in the North-Eastern regions, while the Central regions had the lowest prevalence at 40%. An overall prevalence of 53% was observed among individuals aged 19–59 years. The Northern region exhibited the highest prevalence (64%) and the lowest in the North-Eastern (39%) regions in this age group. Elderly individuals had a prevalence between 52 to 68%, with the highest in the Eastern (65%) and the lowest in the North-Eastern (44%) regions.

**Conclusions:**

Anemia prevalence was highest among toddlers and lowest among school children, with notable regional variations. Cumulative meta-analysis uncovered both consistent and increasing trends across various age groups. This meta-analysis provides essential insights for effective strategies against persistence of anemia prevalence.

**Trial registration:**

PROSPERO registration number is CRD42023431577.

**Supplementary Information:**

The online version contains supplementary material available at 10.1186/s12889-025-22439-3.

## Background

Anemia is a condition characterised by a deficiency in the quantity of red blood cells or a decrease in the concentration of haemoglobin inside these cells, resulting in levels below the established normal range. The presence of haemoglobin is essential for the transportation of oxygen. In cases where there is a deficiency or abnormality in red blood cells, or insufficient levels of haemoglobin, the ability of the blood to effectively deliver oxygen to the body's tissues is compromised [1].

Anemia prevalence in India exceeds that of other developing countries [2] and severe maternal anemia is one of the major causes of adverse fetal and neonatal outcomes in low/middle-income countries [3]. The prevalence of anemia in India poses a significant public health concern. The National Family Health Survey 5 (2019–21) recorded an anemia prevalence of 25.0% in Indian men aged 15–49 years versus a much higher prevalence of 57.0% in women of the same age range. Among adolescent boys and adolescent girls aged 15–19 years, the prevalence was 31.1% and 59.1%, respectively. Pregnant women aged 15–49 years exhibited prevalence of 52.2% and 67.1% among children aged 6–59 months [4]. Anemia imposes a substantial health impact across diverse gender and age categories. To better comprehend anemia prevalence patterns and related risk factors in India, targeted local research specific to age groups and contexts is needed.

Many published articles have examined anemia prevalence across different Indian states [5–7]. The prevalence and associated factors of anemia in different states of India vary due to various conditions such as socioeconomic status, healthcare infrastructure, cultural norms, social support networks, urban–rural differences, educational levels, and government policies. A comprehensive evaluation of anemia prevalence nationally and by state is required to fully grasp its extent and geographic distribution. This will aid in developing targeted prevention programs.

The geospatial analysis is instrumental in understanding the geographical prevalence of anemia across diverse regions and states in India. Furthermore, cumulative meta-analysis will contribute to discerning the trends in the proportion of anemia over the years at a national scale. These estimates may then be used to guide evidence-based decision-making and the implementation of public health initiatives. As per our knowledge, there is a lack of up-to-date systematic reviews and meta-analyses that offer comprehensive estimates of prevalence of anemia among different age groups of Indian population across different regions and states of India. The present study aims to evaluate the anemia prevalence in India and offer an analysis of its spatial distribution across different regions within the country.

## Methods

### Protocol registration

The present protocol has been duly registered with PROSPERO [8] (International Prospective Register of Systematic Reviews) and has been formulated in agreement with the guidelines issued by the Preferred Reporting Items for Systematic Reviews and Meta-analyses Protocol (PRISMA-P). PROSPERO registration number is CRD42023431577.

### Exploration approach of primary studies

Two reviewers independently searched the titles and abstracts for potentially appropriate studies and excluded unrelated studies. If discrepancies arose, they were deliberated upon or escalated to a neutral third party for a final decision. Thereafter, articles meeting the standards were retrieved in full text and reviewed independently by the two authors to make a final selection of studies for review. A comprehensive search was conducted using appropriate keywords to retrieve all relevant articles. The search terms included “India OR Bharat OR Hindustan” AND “Prevalence OR prevalent OR occurrence OR proportion OR frequency OR frequence OR incidence” AND “anemia OR iron deficiency OR anaemia OR bloodlessness OR iron-poor blood OR anemic”. The electronic bibliographic databases namely Scopus, Web of Science, and PubMed were used for the literature search. All search results were imported into Rayyan QCRI software to ensure a systematic and comprehensive search and document the selection process.

### Inclusion criteria and exclusion criteria

#### Participants

Participants eligible for the study include individuals of all age groups from India. There were no specific gender restrictions.

#### Study design

Cross-sectional studies (published from 1995 to June 2023) reporting the prevalence of anemia in India were included. Case studies, qualitative studies and single case series were excluded. The included studies were written in English.

#### Outcome

The primary outcome of the study was prevalence/proportion of anemia. However, upon literature review, studies focusing on anemia in pregnant women were identified and included in the analysis.

Anemia is defined based on haemoglobin (Hb) levels across age groups. For children < 6 years (toddlers and preschool children), anemia is Hb < 110 g/L, while for 6–18 years, cutoffs differ by sex: < 130 g/L for males and < 120 g/L for females. Among adults 19–59 years, anemia is < 130 g/L for men, < 110 g/L for pregnant women. In the elderly (≥ 60 years), anemia is generally < 130 g/L for men and < 120 g/L for women.

### Data extraction

Using a data extraction form, information was extracted from the research articles in a systematic format. Articles with full text from the above mentioned databases were downloaded. The data extracted from them were fed with the following titles into the Microsoft Excel software: Study ID, Year of publication, Year of conduct, Prevalence (p), Sample size (n), Standard error (SE), Region/State, geographical areas namely North (N), South (S), Central (C), West (W), East (E), North East (NE) and gender of the study participants.

### Quality assessments

Quality of the included studies were evaluated using the Newcastle–Ottawa Scale (NOS) adapted for cross-sectional studies. Using the tool, each study is judged on eight items, categorized into three groups: the selection of the study groups; the comparability of the groups; and the ascertainment of either the exposure or outcome of interest. Maximum score of 9 is given to high-quality studies and a score less than 5 indicates weak study [9].

### Statistical analysis

The data were extracted using a Microsoft Excel for management and analysis. The standard error of prevalence for each original article were calculated. Meta-analysis was employed to consolidate prevalence data. The heterogeneity between the prevalence of the studies was checked using the Cochran Q test and I^2^ statistic [10]. Due to the presence of significant heterogeneity (I^2^ > 50%), random effects model (using Restricted Maximum Likelihood method) was performed across all the age groups. Results were visually represented through a forest plot, incorporating individual prevalence, pooled estimates, and 95% confidence intervals. Funnel plot and Egger’s test were used to evaluate the potential publication bias of the included studies. A p-value < 0.05 from Egger’s test indicates evidence of publication bias.

STATA 16 software was used for meta-analysis, subgroup analysis, and sensitivity analysis. The geospatial analysis and cumulative meta-analysis were performed in R 4.3.3 software.

#### Geospatial analysis

The proposed study conducted a geospatial analysis to assess the distribution of the prevalence of anemia among various age groups at different states and union territories of India and also at different geographical regions [11] namely North, South, Central, West, East, and Northeast. The analysis was done by means of choropleth maps in which the prevalence rates within each region were georeferenced and depicted using a colour scale, with higher rates represented by deeper shades of red. The number of studies included in the meta-analysis was indicated by blue circles of varying sizes.

#### Subgroup analysis

Subgroup analysis was done based on the state and union territories, geographical areas and gender of the participants.

#### Sensitivity analysis

Sensitivity analysis was performed by excluding the studies of poor quality i.e., studies with a score less than 5 in the NOS.

#### Cumulative meta-analysis

Cumulative meta-analysis [12] was conducted by sequentially adding studies, ordered by their year of study (provided within the square bracket of the study ID in the cumulative forest plot), to examine the temporal trends in the proportion of anemia.

## Results

### Selection of articles

A total of 157 articles were selected based on the inclusion and exclusion criteria formulated prior to the review of literature. Among these, 148 articles were obtained from PubMed, Scopus and Web of Science database after the entire process of systematic review and the remaining nine articles were included from other sources.

**Fig. 1:**
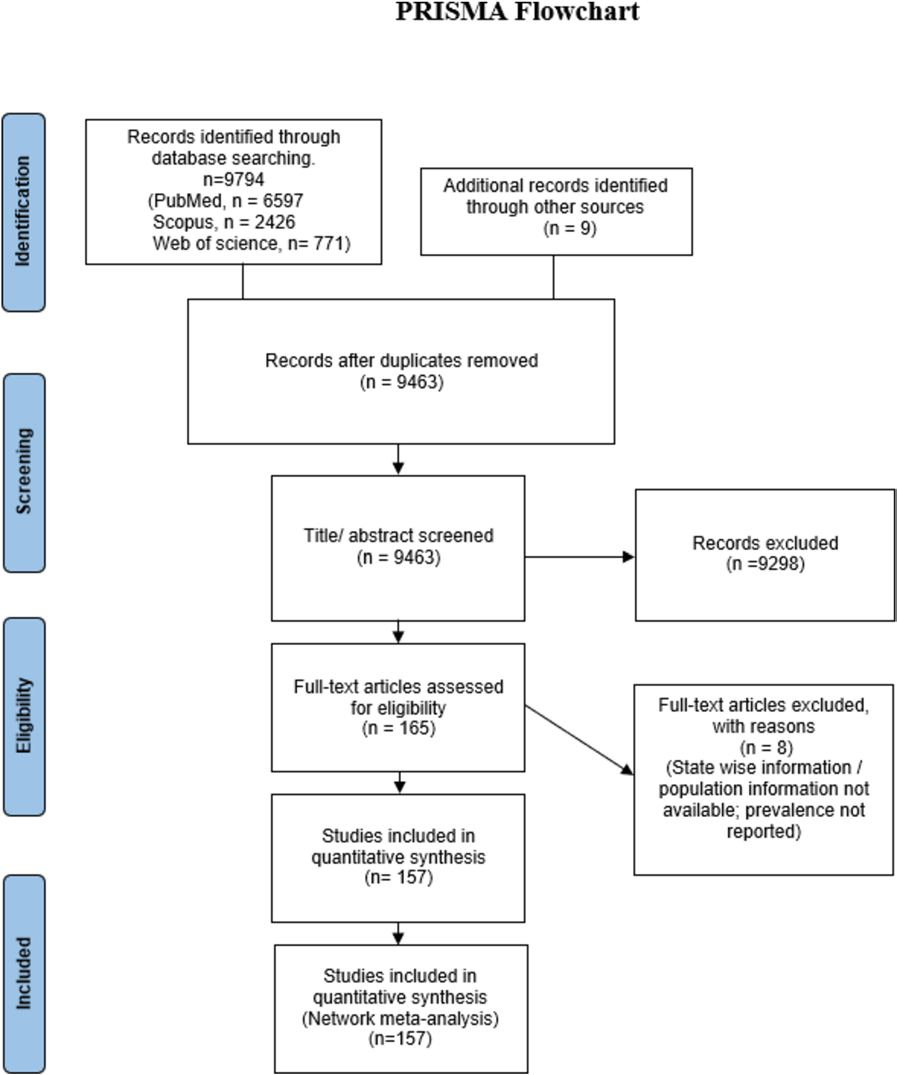
PRISMA flowchart for selection of studies

All the articles provided sufficient data, either in the form of prevalence or proportion. Some of the studies reported data on prevalence among males, females, or both, and for various states. In 151 studies, the prevalence of anemia was reported explicitly whereas for the remaining six studies the prevalence was calculated from the data presented. The characteristics of 157 included studies are provided in the Supplementary Table 1. The entire process of screening is represented using a PRISMA diagram (Fig. 1). The quality assessment was carried out using NOS adapted for cross-sectional studies (Supplementary Table 2). After the quality assessment procedure, all the studies were included in the meta-analysis.

### Pooled prevalence of anemia among toddlers (under 3 years)

**Fig. 2:**
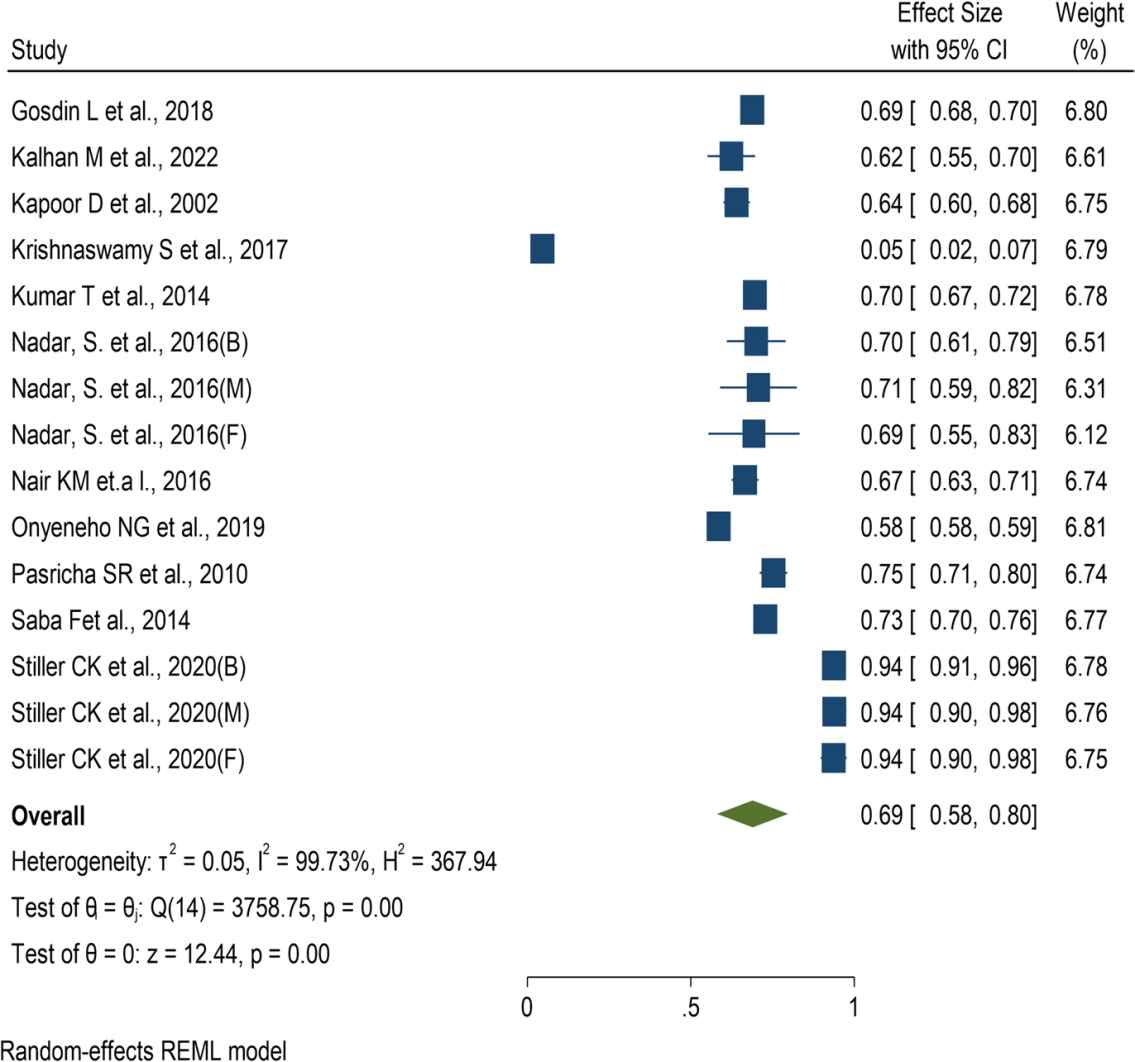
Forest plot of prevalence of anemia among toddlers (under 3 years)

Of the 157 articles reviewed, 11 studies [5–7, 13–20] provided sufficient data for the meta-analysis of anemia prevalence among toddlers. A random-effects meta-analysis was selected due to a high level of heterogeneity, as indicated by an I^2^ statistic of 99.73%. The overall pooled estimate was found to be 69% (95% CI: 58%, 80%) (Fig. 2). A sensitivity analysis, excluding poor-quality studies, indicated that the main meta-analysis is not sensitive to any of the included studies (Supplementary Fig. 1). Cumulative meta-analysis showed no discernible trend over the period from 1997 to 2016 (Supplementary Fig. 2).

Subgroup analysis concerning various regions of India indicated that the Eastern region had the highest pooled prevalence at 87% (95% CI: 75%, 100%), while the Northern region exhibited the lowest pooled prevalence at 50% (95% CI: 20%, 80%). It was also observed that there was a statistically significant difference in the prevalence of anemia in different regions of India (Q_b_ (2) = 8.23, p = 0.02). Among the different states and union territories of India, the highest pooled prevalence was found in West Bengal at 94% (95% CI: 92%, 96%) and the lowest pooled prevalence was found in Chandigarh at 5% (95% CI: 2%, 7%). It was also observed that there was a statistically significant difference in the prevalence of anemia across different states and union territories of India (Q_b_ (7) = 3419.50, p < 0.001). The forest plot of subgroup analysis based on region, state and gender are provided as supplementary files (Supplementary Fig. 3–5).

A few studies reported anemia prevalence separately for males, females, and a mixed group. In the study ID, we denote these as M, F, and B, where M indicates males only, F indicates females only and B indicates both males and females.

### Pooled prevalence of anemia among pre-school children (3–5 years)

**Fig. 3:**
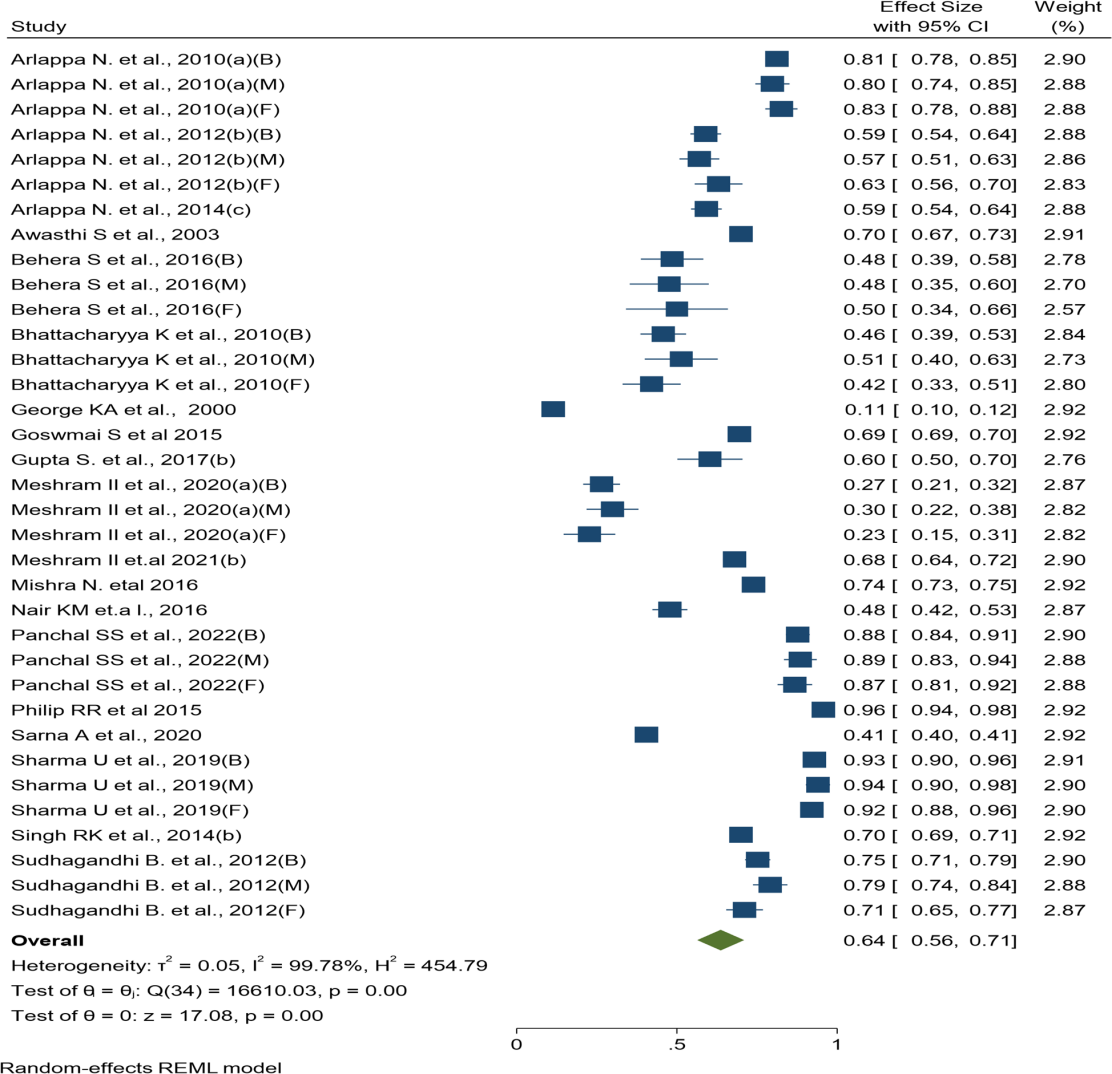
Forest plot of prevalence of anemia among pre-school children (3–5 years)

The meta-analysis of anemia prevalence among pre-school children was based on 19 studies [16, 21–38] out of the 157 reviewed. The overall pooled estimate of anemia prevalence based on the random-effects model was found to be 64% (95% CI: 56%, 71%), provided in Fig. 3. Sensitivity analysis was not performed for this age group as there were no poor-quality studies. A slight upward trend over the period from 1998 to 2018 was observed in the cumulative meta-analysis (Supplementary Fig. 6).

The Central region of India exhibited highest pooled prevalence of 85% (95% CI: 74%, 95%) in this age group, as shown in the subgroup analysis while the North-Eastern region exhibited the lowest pooled prevalence of 37% (95% CI: 16%, 58%). A statistically significant difference in the prevalence of anemia at different regions of India (Q_b_ (5) = 27.57, p < 0.001) was also observed. When examining anemia prevalence by state and union territory, Gujarat and Nagaland demonstrated highest and lowest pooled prevalence at 88% (95% CI: 85%, 90%) and 26% (95% CI: 22%, 30%), respectively. The prevalence of anemia was significantly different across states and union territories (Q_b_ (10) = 754.51, *p* < 0.001). The forest plot of subgroup analysis based on region, state and gender are provided in Supplementary Fig. 7–9.

### Pooled prevalence of anemia among school children (6–18 years)

The pooled prevalence of anemia of 51.2% (95% CI: 46%, 56.4%) among school children was derived from the meta-analysis of 48 articles [39–82] out of 157 articles reviewed. As a large number of studies were included in the meta-analysis, forest plot was not generated (details provided in Supplementary Table 3). The details of sensitivity analysis are provided in Supplementary Table 4 which indicate that the main meta-analysis is not sensitive to any of the included studies. Cumulative meta-analysis revealed that there is an upward trend over the period from 2002 to 2020 (Supplementary Table 5).

In contrast to the results among pre-school children, the highest and the lowest pooled prevalence of anemia among school children were presented in the North-Eastern (83.9%; 95% CI: 59.6%, 108.2%) and Central regions (40%; 95% CI: 29.6%, 50.4%), respectively, based on the subgroup analysis. A statistically significant difference in the prevalence of anemia was observed at different regions (Q_b_ (5) = 23.01, p < 0.001) as well as at different states and union territories of India (Q_b_ (18) = 532.16, *p* < 0.001). Assam had the highest pooled prevalence of 83.9% (95% CI: 59.6%, 108.2%) whereas Chandigarh had the lowest at 15.9% (95% CI: 6.8%, 25%) among the different states and union territories analyzed. The results of subgroup analysis based on region, state and gender are provided as Supplementary Table 6–8.

### Pooled prevalence of anemia among young and middle-aged adults (19–59 years)

**Fig. 4:**
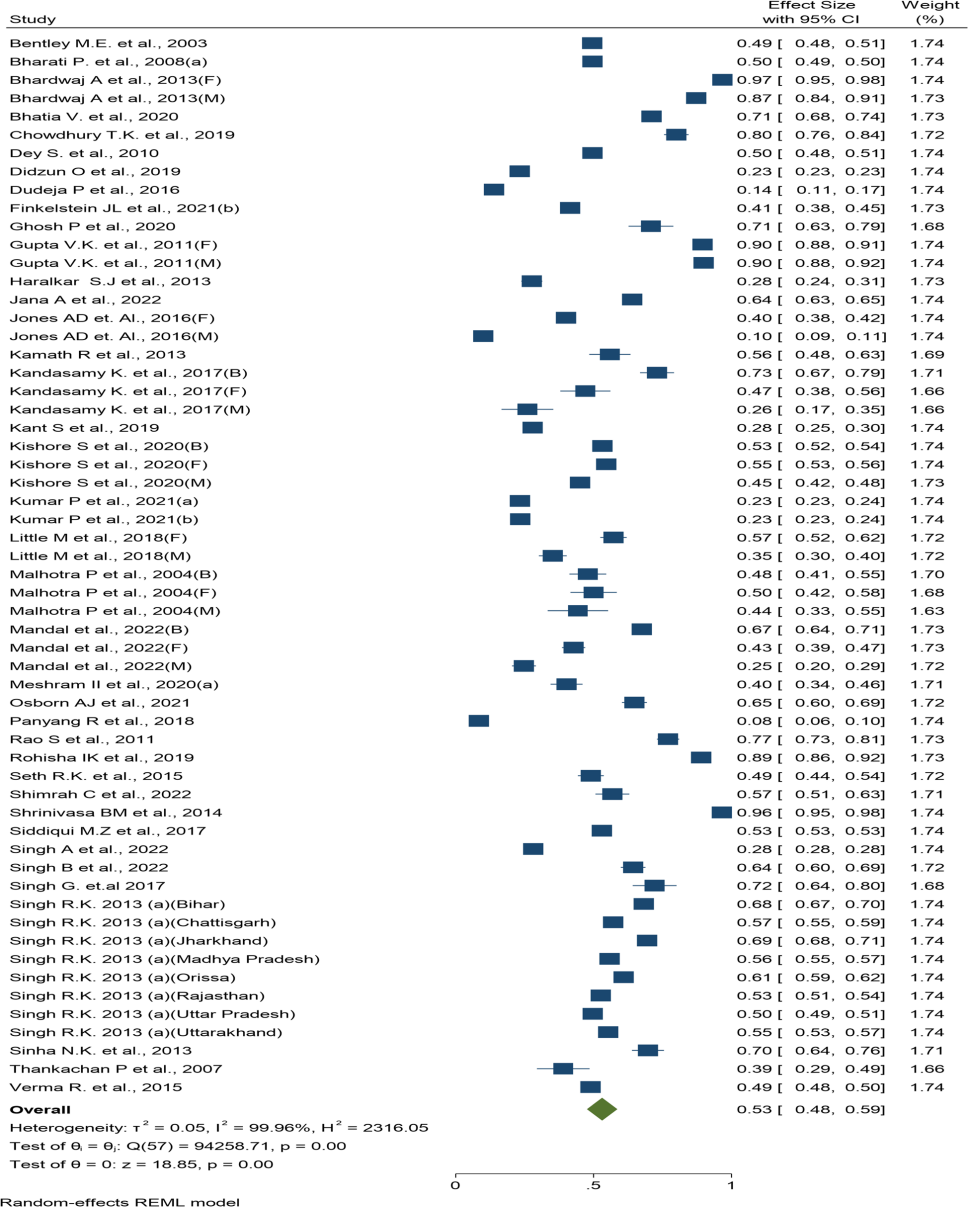
Forest plot of prevalence of anemia- among adults (19–59 years)

Young and middle-aged adults exhibited a pooled anemia prevalence of 53% (95% CI: 48%, 59%), based on 39 studies [83–119] (Fig. 4). Sensitivity analysis (Supplementary Fig. 10) and cumulative meta-analysis (Supplementary Fig. 11) indicated the robustness of meta-analysis against excluded poor-quality studies, and a stable 1995–2022 prevalence trend from the cumulative meta-analysis, respectively.

The subgroup analysis results indicated the highest pooled prevalence of anemia in the Northern region (64%; 95% CI: 48%, 79%) and the lowest prevalence in the North-Eastern region (39%; 95% CI: 18%, 60%). When compared across states and union territories, Kerala demonstrated a prevalence of anemia at 92.8% (95% CI: 85.5%, 100.2%), however, these estimates were obtained from two studies conducted among the tribal population. Himachal Pradesh demonstrated the highest prevalence of 92.1% (95% CI: 82.8%, 101.4%) and Assam demonstrated the lowest pooled prevalence at 8.4% (95% CI: 6.4%, 10.4%). The prevalence of anemia demonstrated statistical significance in the analyses of individual state and union territory-level subgroups (Q_b_ (18) = 7187.44, p < 0.001) but not at the region level (Q_b_ (5) = 6.98, p = 0.22). The forest plot of subgroup analysis based on region, state and gender are provided as Supplementary Fig. 12–13 and Supplementary Table 9.

### Pooled prevalence of anemia among elderly (≥ 60 years)

**Fig. 5:**
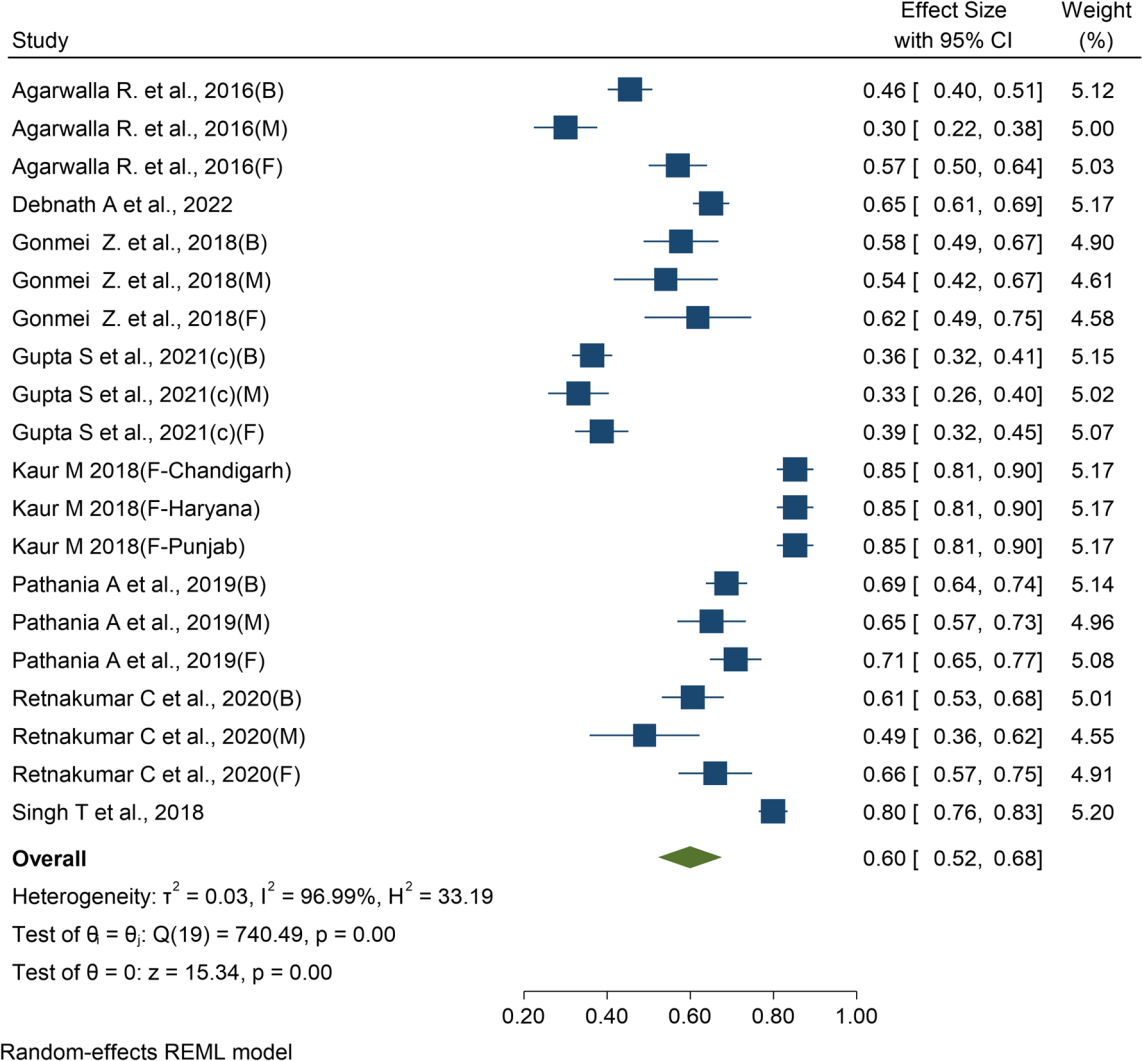
Forest plot of prevalence of anemia among elderly persons (≥ 60 years)

Out of the 157 articles reviewed, eight articles [120–127] provided data relevant for the meta-analysis of elderly persons. The overall pooled estimate found to be 60% (95% CI: 52%, 68%) (Fig. 5). A sensitivity analysis excluding poor-quality studies showed the main meta-analysis to be robust to the inclusion of any individual study (Supplementary Fig. 14). Cumulative meta-analysis showed a slight decreasing trend in the data over the period from 2010 to 2021 (Supplementary Fig. 15).

A subgroup analysis by region found the highest pooled prevalence in Eastern region at 65% (95% CI: 61%, 69%), while the North-Eastern region exhibited the lowest pooled prevalence at 44% (95% CI: 29%, 59%). It was observed that the prevalence of anemia differed significantly between Indian states and union territories (Q_b_ (6) = 100.9, *p *< 0.001). Chandigarh and Punjab had the highest pooled prevalence at 85% (95% CI: 81%, 90%), while Assam had the lowest at 44% (95% CI: 29%, 59%). The forest plot of subgroup analysis based on region, state and gender are provided as Supplementary Fig. 16–18.

### Pooled prevalence of anemia among pregnant women

**Fig. 6:**
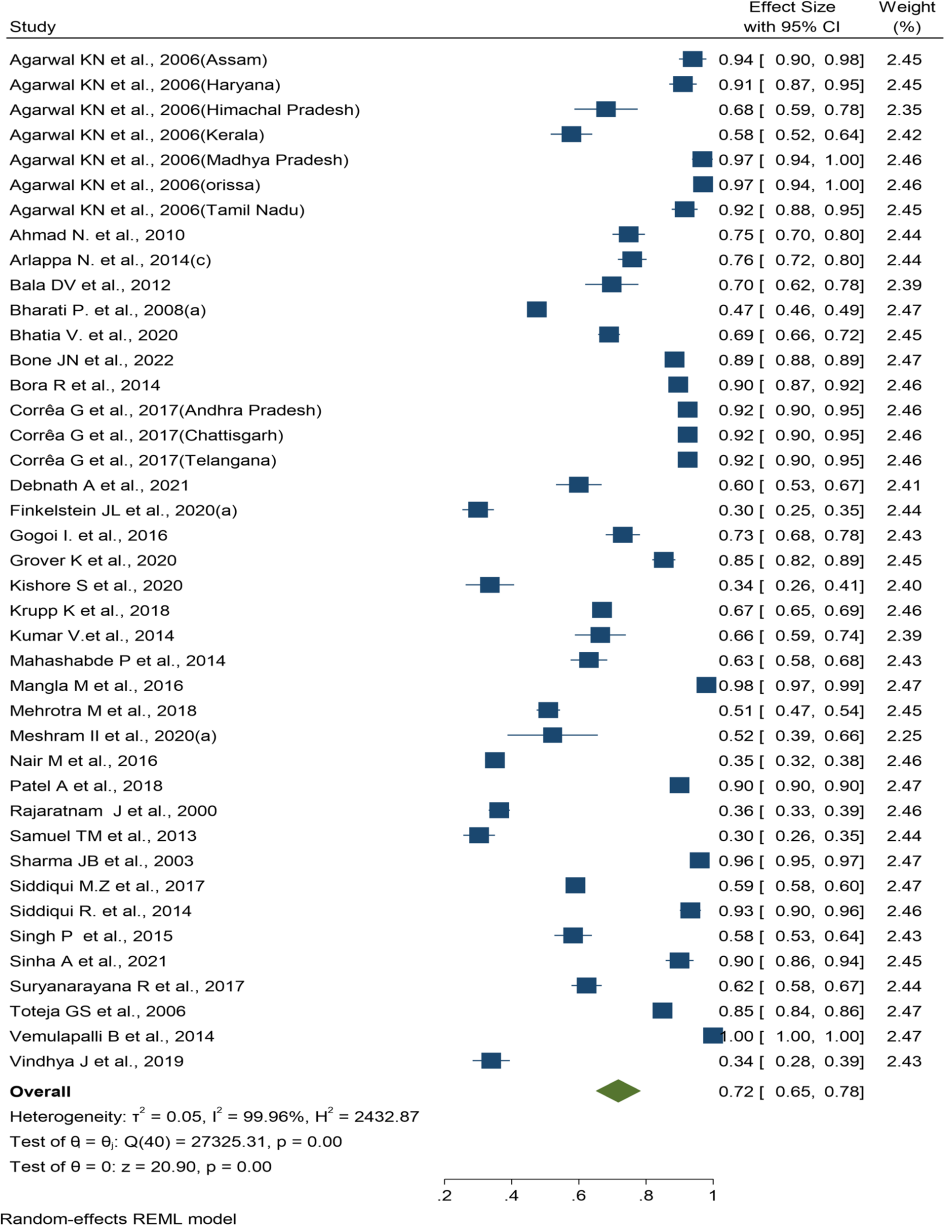
Forest plot of prevalence of anemia among pregnant women

Of the 157 articles reviewed, 33 [128–153] met the inclusion criteria and were included in the meta-analysis to obtain the pooled prevalence of anemia among pregnant women. The overall pooled estimate was found to be 72% (95% CI: 65%, 78%) (Fig. 6). A stable trend in the anemia prevalence within this group was observed over the period of 2010 to 2021 (Supplementary Fig. 19). Subgroup analysis stratified by region in India indicated that the Northern region had the highest pooled prevalence at 88% (95% CI: 79%, 98%), while the Southern region exhibited the lowest pooled prevalence at 67% (95% CI: 53%, 82%). Among the states and union territories across the country, Andhra Pradesh (96.3%; 95% CI: 88.8%, 103.7%) and Uttarakhand (33.5%; 95% CI: 26.3%, 40.7%) had the highest and least pooled prevalence of anemia among pregnant women, respectively. The forest plot of subgroup analysis based on region and state are provided as Supplementary Fig. 20 and Supplementary Table 10. The review also resulted in studies focused on anemia prevalence among different populations, such as antenatal, lactating, postnatal, and delivering women etc [154–166]. The results of meta-analysis for these groups are provided in Supplementary Fig. 21–25.

### Pooled prevalence of severe anemia across different age groups

As most of the studies reported the proportion of severe anemia, we also estimated the pooled prevalence of severe anemia separately for each age group using a random-effects model, as heterogeneity was found to be high (Supplementary Fig. 26–31). The results showed that the pooled prevalence of severe anemia was 9% (95% CI: 1%, 16%) among toddlers; 7% (95% CI: 2%, 12%) among preschool children (3–5 years); 10% (95% CI: 5%, 15%) among school-aged children (6–18 years); 4% (95% CI: 3%, 5%) among young and middle-aged adults (19–59 years); 2% (95% CI: 1%, 4%) among the elderly (≥ 60 years); and 6% (95% CI: 4%, 9%) among pregnant women.

### Publication bias

Publication bias was evaluated using funnel plots, which appeared symmetrical (Supplementary Fig. 32–37), indicating no potential bias for all age groups except for anemia among pre-school children and pregnant women. This finding was further confirmed by the regression-based Egger’s test. The Egger’s test p-values were 0.768 for the < 3 years group, 0.034 for the 3–5 years group, 0.183 for the 6–18 years group, 0.846 for the 19–59 years group, 0.128 for the elderly and 0.0008 for pregnant women, respectively.

### Geospatial analysis

**Fig. 7:**
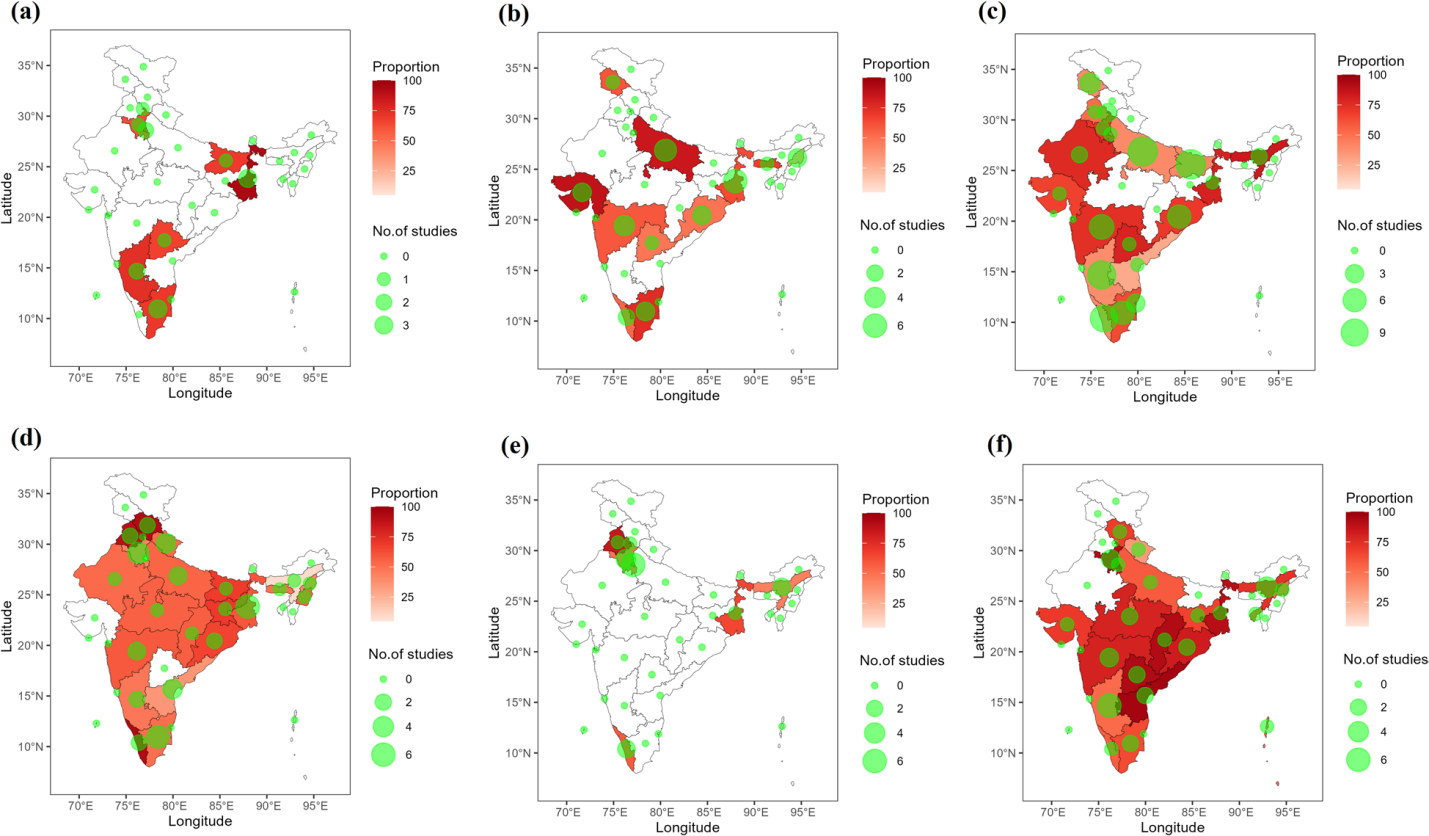
Spatial distribution of prevalence of anemia based on states and union territories, **a** toddlers; **b** pre-school children; **c** school going children; **d** adults; **e **elderly and **f** pregnant women

The Choropleth maps illustrating the prevalence of anemia by state and age group are shown in Fig. 7. The deepest red regions of the choropleth map depicting the highest pooled prevalence of anemia among toddlers occurred in the state of West Bengal and the lowest in the union territory Chandigarh (Fig. 7a). Additionally, inspecting the red colour gradations across the other state-level maps (Fig. 7b-7f) shows the specific states/territories with the highest versus lowest anemia prevalence within each age group.

## Discussion

This meta-analysis comprised a total of 157 studies, encompassing diverse age categories and including studies focused on pregnant women. The highest pooled prevalence of anemia was observed among toddlers, while the lowest prevalence was found among school children. The age group of 6 to 18 years had the highest number of studies (45), whereas the least number of studies were reported for toddlers. Irrespective of age groups, the majority of studies were published from the southern region, with fewer publications from the northeast. Notably, there were no published articles on toddlers from the central, west, and northeast regions. However, for pre-school children, school children, and adults, publications were available from all areas of India. Among the elderly, no studies have been reported from the central and west regions of India. Generalizability and comparison of estimates across different regions depends on the number of included studies and characteristics of the study population.

In toddlers under 3 years old, a substantial overall pooled anemia prevalence of 69% is highlighted, with the East region, particularly West Bengal, exhibiting the highest rates (94%), and Chandigarh in the North reporting the lowest (5%). The absence of gender-based differences and the stable trend over 1997 to 2016 underscore the persisting challenge of anemia in this age group. Similarly, in preschool children (3–5 years), the pooled prevalence of 64% raises significant public health concerns. Regional disparities are evident, with the Central region having the highest prevalence (85%) and the North-East region the lowest (37%). Gujarat records a prevalence of 88%, emphasizing the need for targeted interventions. The upward trend identified from 1998 to 2018 needs continuous monitoring and proactive measures. School children exhibit a 51.2% pooled anemia prevalence, with pronounced regional variations. The North-East region, particularly Assam, reports the highest rates (83.9%), while the Central region shows the lowest (40%). Despite state-level extremes, with Assam at the top (83.9%) and Chandigarh at the bottom (15.9%), the cumulative meta-analysis from 2002 to 2020 reveals an overall upward trend, emphasizing the urgency of sustained efforts. A systematic review by Daniel et al [167] in 2023 reported that the pooled prevalence of anaemia among adolescent girls in India was 65.7% (95% CI 59.3%–71.9%), indicating a significant public health concern. Another systematic review by Rakesh et al [168] in 2017 conducted in Kerala found that anaemia prevalence among tribal women and children remained disproportionately high, ranging from 78.3% to 96.5%.

Analysis of individuals aged 19–59 years presents a 53% overall pooled prevalence, with notable regional differences. Kerala reports the highest prevalence (92.8%), while Assam records the lowest (8.4%). The stable trend from 1995 to 2022 underlines the persistent burden of anemia in this age group, reinforcing the importance of continued monitoring. For elderly individuals (≥ 60 years), the 60% overall pooled prevalence underscores the need for targeted interventions, with notable regional variations. The stability identified from 2010 to 2021 calls for sustained efforts to address anemia in this vulnerable demographic. Gender-based differences, although statistically significant, require further exploration. A systematic review by Daniel et al., 2023, [169] revealed that the pooled prevalence of anemia among the elderly in India to be 68.3% (95% CI: 60.7%–75.9%).

In pregnant women, the 72% overall pooled prevalence emphasizes the critical concern of anemia during pregnancy. Regional disparities are evident, with Andhra Pradesh reporting the highest (96.3%) and Uttarakhand the lowest (33.5%). The consistent prevalence rate over 1996 to 2019 underscores the persistent nature of the issue. Sensitivity analyses across all demographic groups confirm the robustness of the primary meta-analysis. A 2013 study by Stevens G. A. [170] reported a decline in anemia prevalence among pregnant women from 43 to 38%. The study analyzed global trends in hemoglobin levels and anemia prevalence from 1995 to 2011, using data from 257 sources across 107 countries. Findings indicated a slight global increase in mean hemoglobin and a reduction in anemia prevalence, though millions remained affected, particularly in South Asia and central and west Africa.

The high heterogeneity observed in this meta-analysis, as indicated by the I^2^ value, may be attributed to variations in anemia prevalence across different geographical regions, as explored through subgroup and geospatial analyses. Additional clinical factors contributing to this heterogeneity include differences in dietary patterns, socioeconomic status, healthcare access, and underlying health conditions such as infections, parasitic diseases, and genetic hemoglobin disorders. Despite these variations, the use of a random-effects model accounts for between-study differences, enhancing the robustness of our findings.

This meta-analysis underscores the persistent burden of anaemia across all age groups in India, with significant regional disparities. The high prevalence among toddlers, pregnant women, and the elderly highlights the need for targeted interventions. Despite existing public health efforts, stable or rising trends suggest gaps in implementation. Socio-economic and healthcare disparities further emphasize the necessity of region-specific strategies. Continued research, especially in underrepresented groups, is crucial to refining interventions and reducing the anaemia burden nationwide.

When compared to national estimates from the National Family Health Survey-5 (NFHS-5, 2019–21) [171], our prevalence estimates for children under five (64–69%) and pregnant women (72%) are slightly higher than NFHS-5 findings, which reported anemia prevalence of 67.1% in children (6–59 months) and 52.2% in pregnant women (15–49 years). Among adults, NFHS-5 reported anemia prevalence of 25.0% in men and 57.0% in women (15–49 years), while our estimate for the general adult population (19–59 years) was 53%.

Globally, anemia remains a significant public health concern, particularly in low- and lower-middle-income countries where it disproportionately affects rural populations, economically disadvantaged groups, and those with limited education. According to WHO estimates [172], anemia affects 40% of children aged 6–59 months, 37% of pregnant women, and 30% of women aged 15–49 years, contributing to 50 million years of healthy life lost due to disability in 2019. The primary causes include dietary iron deficiency, genetic blood disorders (thalassemia and sickle cell trait), and infectious diseases like malaria.

The higher prevalence observed in our meta-analysis compared to NFHS-5 and WHO estimates may be attributed to differences in study settings, socioeconomic factors, dietary habits, healthcare access, and variations in diagnostic criteria. Additionally, our study may have included populations at higher risk of anemia, leading to elevated prevalence rates.

Recognizing this ongoing challenge, India launched the Anemia Mukt Bharat initiative under the National Nutrition Strategy and POSHAN Abhiyaan, aiming to reduce anemia prevalence by three percentage points annually among children, adolescents, and women of reproductive age from 2018 to 2022. Additionally, the government introduced Mission Poshan 2.0 and the Rice Fortification Initiative to enhance iron intake through fortified foods. These large-scale programs are expected to have a significant impact on anemia reduction. The forthcoming NFHS-6 (2023–24) data will be crucial in assessing the effectiveness of these interventions and guiding future policy refinements for sustainable improvements in anemia prevalence.

The geospatial analysis in this study provides critical insights into the regional distribution of anemia. Region-specific variations in prevalence of anaemia need further explorations for implementation challenges for factors like socio-economic, cultural, nutrition and region-specific health issues (Parasitic infections/ helminth infestation). Future research should focus on identifying specific risk factors contributing to anaemia in various age groups and geographical regions. Policy-makers can utilize data of present review to allocate resources efficiently, ensuring high-burden areas receive intensified interventions. Customizing anaemia control programs based on regional and demographic needs will enhance their effectiveness.

### Strength and limitations

The strength of this study lies in its ability to offer a comprehensive analysis of anemia prevalence in India across different age groups and regions. Through cumulative meta-analysis, it tracks the evolving trends in anemia prevalence over time. The geospatial analysis reveals the geographical distribution of anemia throughout India. However, using only cross-sectional studies in this review may limit establishing causal relationships due to their snapshot nature. Despite thorough efforts, some studies may have been missed, potentially impacting the completeness of the findings. Additionally, restricting the review to English articles might have excluded valuable insights from non-English studies.

## Conclusion

The present study reveals that the anemia prevalence is highest among toddlers and lowest among school children. The spatial analysis revealed that there is significant difference in the anemia prevalence across various regions and states of India. A cumulative meta-analysis revealed persistent and growing patterns of prevalence across diverse age categories. This current and comprehensive meta-analysis, which focuses on anemia prevalence among various age groups in the Indian population, alongside spatial distribution data, has the potential to offer invaluable support to governments, healthcare providers, and local communities. It highlights the multifaceted nature of anemia prevalence in India, urging targeted interventions, continuous monitoring, and sustained efforts across diverse demographic segments.

## Supplementary Information

Supplementary Material 1.

## Data Availability

Data supporting this meta-analysis can be obtained through corresponding author on special request.

## References

[1] World Health Organization. Health topics: anaemia. 2018. Available from: https://www.who.int/health-topics/anaemia#tab=tab_1. Cited 2019 Mar 20.

[2] Stevens GA, Finucane MM, De-Regil LM, et al. Global, regional, and national trends in hemoglobin concentration and prevalence of total and severe anemia in children and pregnant and non-pregnant women for 1995–2011: A systematic analysis of population representative data. Lancet Glob Health. 2013;1:E16-25. 25103581 10.1016/S2214-109X(13)70001-9 PMC4547326

[3] Parks S, Hoffman MK, Goudar SS, et al. Maternal anaemia and maternal, fetal, and neonatal outcomes in a prospective cohort study in India and Pakistan. BJOG. 2019;126(6):737–43. 30554474 10.1111/1471-0528.15585 PMC6459713

[4] Anaemia Mukt Bharat. Ministry of health and family welfare. Available from: https://pib.gov.in/PressReleasePage.aspx?PRID=1795421. Accessed 4 Feb 2022.

[5] Gosdin L, Martorell R, Bartolini RM, Mehta R, Srikantiah S, Young MF. The co-occurrence of anaemia and stunting in young children. Matern Child Nutr. 2018;14(3): e12597. 29468825 10.1111/mcn.12597 PMC6866136

[6] Kalhan M, Kaushal P, Chayal V, et al. Prevalence of anemia among toddlers (12–36 months) in urban area of district Rohtak. Haryana J Fam Med Prim Care. 2022;11(6):2532–6. 10.4103/jfmpc.jfmpc_1469_21 PMC9480634 36119233

[7] Kapoor D, Agarwal KN, Sharma S, Kela K, Kaur I. Iron status of children aged 9–36 months in an urban slum Integrated Child Development Services project in Delhi. Indian Pediatr. 2002;39(2):136–44. 11867843

[8] Tawfik GM, Dila KA, Mohamed MY, Tam DN, Kien ND, Ahmed AM, Huy NT. A step by step guide for conducting a systematic review and meta-analysis with simulation data. Trop Med Health. 2019;47:1–9. 31388330 10.1186/s41182-019-0165-6 PMC6670166

[9] Peterson J, Welch V, Losos M, Tugwell P. The Newcastle-Ottawa scale (NOS) for assessing the quality of nonrandomised studies in meta-analyses. Ottawa: Ottawa Hospital Research Institute; 2011.

[10] Higgins JPT, Thomas J, Chandler J, Cumpston M, Li T, Page MJ, Welch VA (editors). Cochrane handbook for systematic reviews of interventions. 2nd Ed. Chichester (UK): Wiley; 2019.

[11] Pilania M, Yadav V, Bairwa M, et al. Prevalence of depression among the elderly (60 years and above) population in India, 1997–2016: a systematic review and meta-analysis. BMC Public Health. 2019;19:832. 31248394 10.1186/s12889-019-7136-z PMC6598256

[12] Behera P, Pilania M, Yadav V, et al. Estimation of the prevalence of depression using diagnostic instruments in the elderly population in India, 2000–2019: a systematic review protocol. BMJ Open. 2020;10: e034330. 32385060 10.1136/bmjopen-2019-034330 PMC7228514

[13] Krishnaswamy S, Bhattarai D, Bharti B, Bhatia P, Das R, Bansal D. Iron deficiency and iron deficiency anemia in 3–5 months-old. Breastfed Healthy Infants Indian J Pediatr. 2017;84(7):505–8. 28321611 10.1007/s12098-017-2330-4

[14] Kumar T, Taneja S, Yajnik CS, Bhandari N, Strand TA; Study Group. Prevalence and predictors of anemia in a population of North Indian children. Nutrition. 2014;30(5):531–7. 24560137 10.1016/j.nut.2013.09.015

[15] Nadar S, Vijaykumar M, Gheena R. Prevalence of anemia in urban children attending a pediatric hospital of a metro city in South India. Res J Pharm Technol. 2016;9:1571–4.

[16] Nair KM, Fernandez-Rao S, Nagalla B, et al. Characterisation of anaemia and associated factors among infants and pre-schoolers from rural India. Public Health Nutr. 2016;19(5):861–71. 26139153 10.1017/S1368980015002050 PMC10271129

[17] Onyeneho NG, Ozumba BC, Subramanian SV. Determinants of childhood anemia in India. Sci Rep. 2019;9(1):16540. 31719548 10.1038/s41598-019-52793-3 PMC6851096

[18] Pasricha SR, Black J, Muthayya S, et al. Determinants of anemia among young children in rural India. Pediatrics. 2010;126(1):e140–9. 20547647 10.1542/peds.2009-3108

[19] Saba F, Poornima S, Balaji PA, Varne SR, Jayashree K. Anemia among hospitalized children at a multispecialty hospital, Bangalore (Karnataka). India J Fam Med Prim Care. 2014;3(1):48–53. 10.4103/2249-4863.130275 PMC4005201 24791237

[20] Stiller CK, Golembiewski SKE, Golembiewski M, Mondal S, Biesalski HK, Scherbaum V. Prevalence of undernutrition and anemia among santal adivasi children, Birbhum District, West Bengal, India. Int J Environ Res Public Health. 2020;17(1): 342. 31947849 10.3390/ijerph17010342 PMC6981430

[21] Arlappa N, Balakrishna N, Laxmaiah A, Brahmam GN. Prevalence of anaemia among rural pre-school children of West Bengal. India Ann Hum Biol. 2010;37(2):231–42. 19657766 10.1080/03014460902991979

[22] Arlappa N, Balakrishna N, Laxmaiah A, Brahmam G. Prevalence of anaemia among rural pre-school children of Maharashtra, India. Indian J Community Health. 2012;24(1):4–8.

[23] Arlappa N, Meshram II, Balakrishna N, Harikumar R, Rao KM, Laxmaiah A. Prevalence of anaemia among different physiological groups in the rural areas of Maharashtra. Indian J Community Health. 2014;26(3):278–84.

[24] Awasthi S, Das R, Verma T, Vir S. Anemia and undernutrition among preschool children in Uttar Pradesh. India Indian Pediatr. 2003;40(10):985–90. 14581738

[25] Behera S, Bulliyya G. Magnitude of anemia and hematological predictors among children under 12 years in Odisha. India Anemia. 2016;2016:1729147. 27127647 10.1155/2016/1729147 PMC4834407

[26] Bhattacharyya K, Sarkar TK. Nutritional profile of children under 5 years of age in a tribal community in the district of Maldah, West Bengal. J Prim Care Community Health. 2010;1(3):184–6. 23804609 10.1177/2150131910378692

[27] George KA, Kumar NS, Lal JJ. Anaemia and nutritional status of pre-school children in Kerala. Indian J Pediatr. 2000;67:575. 10984998 10.1007/BF02758483

[28] Goswmai S, Das KK. Socio-economic and demographic determinants of childhood anemia. J Pediatr (Rio J). 2015;91(5):471–7. 26070864 10.1016/j.jped.2014.09.009

[29] Gupta S, Gupta A, Raina B, Khajuria A. Prevalence and pattern of anaemias in children at ASCOMS & hospital Jammu. JK Science. 2017;19(2):76–80.

[30] Meshram II, Kumar BN, Venkaiah K, Longvah T. Subclinical vitamin A deficiency and anemia among women and preschool children from Northeast India. Indian J Community Med. 2020;45(3):371–4. 33354022 10.4103/ijcm.IJCM_356_19 PMC7745803

[31] Meshram II, Neeraja G, Longvah T. Vitamin A deficiency, anemia, and nutritional status of under 5-year children from Northeast India. Indian J Community Med. 2021;46(4):673–9. 35068732 10.4103/ijcm.IJCM_62_21 PMC8729277

[32] Mishra N, Kumar S, Parveen K. Essential determinants of anaemia among children of Uttar Pradesh (India): evidence from National Family Health Surveys. Indian J Community Health. 2016;28(3):254–9.

[33] Panchal SS, Mishra U, Kothari C, et al. Prevalence of anemia in pre-school tribal children with reference to parasitic infections and nutritional impact. J Taibah Univ Med Sci. 2022;17(6):1087–93. 36212591 10.1016/j.jtumed.2022.05.002 PMC9519373

[34] Philip RR, Vijayakumar K, Indu PS, Shrinivasa BM, Sreelal TP, Balaji J. Prevalence of undernutrition among tribal preschool children in Wayanad district of Kerala. Int J Adv Med Health Res. 2015;2:33–8.

[35] Sarna A, Porwal A, Ramesh S, et al. Characterisation of the types of anaemia prevalent among children and adolescents aged 1–19 years in India: a population-based study. Lancet Child Adolesc Health. 2020;4(7):515–25. 32562633 10.1016/S2352-4642(20)30094-8

[36] Sharma U, Yadav N. Prevalence and risk factors of anemia and zinc deficiency among 4-6-year-old children of Allahabad District, Uttar Pradesh. Indian J Public Health. 2019;63(1):79–82. 30880742 10.4103/ijph.IJPH_342_17

[37] Singh RK, Patra S. Extent of anaemia among preschool children in EAG States, India: a challenge to policy makers. Anemia. 2014;2014: 868752. 25140250 10.1155/2014/868752 PMC4129919

[38] Sudhagandhi B, Prema A, Sundaresan S, William WE, Shivashekar G. Anemia in toddlers of Kattankulathur, Kancheepuram District,(Tamil Nadu) India. Int J of Pharma Bio. 2012;3(1):B687–92.

[39] Ahankari AS, Myles PR, Fogarty AW, Dixit JV, Tata LJ. Prevalence of iron-deficiency anaemia and risk factors in 1010 adolescent girls from rural Maharashtra, India: a cross-sectional survey. Public Health. 2017;142:159–66. 27592006 10.1016/j.puhe.2016.07.010

[40] Banerjee M, Bhatti BVK, Roy D, Tomo S. A national survey of the prevalence of anemia and obesity in Indian School Children. J Community Hosp Intern Med Perspect. 2022;12(5):48–53. 36262494 10.55729/2000-9666.1110 PMC9529649

[41] Basu S, Basu S, Hazarika R, Parmar V. Prevalence of anemia among school going adolescents of Chandigarh. Indian Pediatr. 2005;42(6):593–7. 15995276

[42] Bharati P, Shome S, Chakrabarty S, Bharati S, Pal M. Burden of anemia and its socioeconomic determinants among adolescent girls in India. Food Nutr Bull. 2009;30(3):217–26. 19927601 10.1177/156482650903000302

[43] Bhatia V, Parida SP, Mahajan PB, Sahoo DP, Bhattacharjee S. A community based study of anaemia burden using hemocue 201 in Eastern India. Indian J Community Health. 2020;32(2):365–70.

[44] Biradar SS, Biradar SP, Alatagi AC, Wantamutte AS, Malur PR. Prevalence of anaemia among adolescent girls: A one year cross-sectional study. J Clin Diagn Res. 2012;6:372–7.

[45] Bulliyy G, Mallick G, Sethy GS, Kar SK. Hemoglobin status of non-school going adolescent girls in three districts of Orissa, India. Int J Adolesc Med Health. 2007;19(4):395–406. 18348415

[46] Chandrakumari AS, Sinha P, Singaravelu S, Jaikumar S. Prevalence of anemia among adolescent girls in a rural area of Tamil Nadu. India J Family Med Prim Care. 2019;8(4):1414–7. 31143731 10.4103/jfmpc.jfmpc_140_19 PMC6510068

[47] Chaudhary SM, Dhage VR. A study of anemia among adolescent females in the urban area of Nagpur. Indian J Community Med. 2008;33(4):243–5. 19876498 10.4103/0970-0218.43230 PMC2763695

[48] Chauhan S, Kumar P, Marbaniang SP, Srivastava S, Patel R. Prevalence and predictors of anaemia among adolescents in Bihar and Uttar Pradesh, India. Sci Rep. 2022;12(1):8197. 35581388 10.1038/s41598-022-12258-6 PMC9114399

[49] Gopalakrishnan S, Eashwar VMA, Muthulakshmi M, Geetha A. Intestinal parasitic infestations and anemia among urban female school children in Kancheepuram district, Tamil Nadu. J Family Med Prim Care. 2018;7(6):1395–400. 30613531 10.4103/jfmpc.jfmpc_89_18 PMC6293916

[50] Goyle A Jr, Prakash S. Iron status of adolescent girls (10–15 years) attending a government school in Jaipur City, Rajasthan. India Malays J Nutr. 2009;15(2):205–11. 22691818

[51] Gupta S, Taraphdar P, Roy TG, et al. The silent burden of anemia in school age children: a community based study in West Bengal. Indian J Med Sci. 2012;66(7–8):163–8. 23807035

[52] Gunjal SS, Narlawar UW, Humne AY, Chaudhari VVL. Prevalence of sickle cell disorder and anaemia in tribal school students from central India. Int J Collab Res Internal Med Public Healh. 2012;4(6):1321–9.

[53] Jain T, Chopra H, Mohan Y, Rao S. Prevalence of anemia and its relation to socio-demographic factors: crosssectional study among adolescent boys in urban Meerut, India. Biol Med. 2011;3:1–5.

[54] Kumar KJ, Chethak KB, Rama HV, et al. Prevalence of anaemia and undernutrition among street children in Mysuru. India Sri Lanka J Child Health. 2017;46(1):44–7.

[55] Kamble BD, Gunjan M, Sumit J, Singh SK, Jha D, Singh S. Prevalence of anaemia among school going adolescent girls attending Test, Treat and Talk (T-3) camp under Anaemia Mukt Bharat in Delhi. J Family Med Prim Care. 2021;10(2):898–903. 34041095 10.4103/jfmpc.jfmpc_1510_20 PMC8138413

[56] Kumar SV, Harshita S. A study on prevalence of anemia in school going Yanadi tribal children of Nellore District, Andhra Pradesh, India. Rom J Pediatr. 2023;72:11–7.

[57] Kumari R, Bharti RK, Singh K, et al. Prevalence of iron deficiency and iron deficiency anaemia in adolescent girls in a tertiary care hospital. J Clin Diagn Res. 2017;11(8):BC04–6. 28969109 10.7860/JCDR/2017/26163.10325 PMC5620749

[58] Mahanta TG, Mahanta BN, Gogoi P, Dixit P, Joshi V, Ghosh S. Prevalence and determinants of anaemia and effect of different interventions amongst tea tribe adolescent girls living in Dibrugarh district of Assam. Clin Epidemiol Global Health. 2015;3(2):85–93.

[59] Manjula AA, Aravindan KP. Nutritional status of children in different types of schools. 2003. http://www.cds.ac.in/krpcds/report/Aravindan.pdf. Accessed 20 Oct 2016.

[60] Muthayya S, Thankachan P, Zimmermann MB, et al. Low anemia prevalence in school-aged children in Bangalore, South India: possible effect of school health initiatives. Eur J Clin Nutr. 2007;61(7):865–9. 17251926 10.1038/sj.ejcn.1602613

[61] Nair A, Doibale MK. Prevalence of anemia among adolescent girls in rural area of a district of Maharashtra. Indian J Community Health. 2023;35(1):21–6.

[62] Prabhakar SJ, Gangadhar MR. Prevalence of anaemia in Jenukuruba Primitive Tribal Children of Mysore District. Karnataka Anthropol. 2009;11:49–51.

[63] Rahman MHU, Chauhan S, Patel R, et al. Anaemia among Indian children: A study of prevalence and associated factors among 5–9 years old. Child Youth Serv Rev. 2020;119: 105529.

[64] Rai RK, Shinde S, De Neve JW, Fawzi WW. Predictors of incidence and remission of anemia among never-married adolescents aged 10–19 years: a population-based prospective longitudinal study in India. Curr Dev Nutr. 2023;7(3):100031. 37181932 10.1016/j.cdnut.2023.100031 PMC10111602

[65] Rakesh PS, George LS, Joy TM, et al. Anemia among school children in Ernakulam District, Kerala, India. Indian J Hematol Blood Transfus. 2019;35(1):114–8. 30828157 10.1007/s12288-018-1001-6 PMC6369096

[66] Rakesh SR, Asimsha A, Shanavas A, et al. Anaemia among school children from a rural area in Kollam District. Kerala Kerala Med J. 2014;3(15):359–61.

[67] Ramesh Masthi NR, Sathish Chandra MR, Undi M, Aravind M, Puthussery YP. Global positioning system: a new tool to measure the distribution of anemia and nutritional status of children (5–10 years) in a rural area in south India. Indian J Med Sci. 2012;66(1–2):13–22. 23603568

[68] Rakesh PS, Rajeswaran T, Rakesh R, Gigil M, Sheeja AL, Subhagan S. Anaemia among schoolchildren from southern Kerala, India: a cross-sectional study. Natl Med J India. 2015;28(5):225–7. 27132950

[69] Sahoo J, Epari V, Panigrahi SK, et al. Challenges in detection of adolescent anaemia: validation of point-of-care device (Mission® plus) for haemoglobin measurement among tribal residential school children of selected districts of Odisha, India. Indian J Community Med. 2021;46(4):680–4. 35068733 10.4103/ijcm.IJCM_96_21 PMC8729287

[70] Sen A, Kanani SJ. Deleterious functional impact of anemia on young adolescent school girls. Indian Pediatr. 2006;43(3):219–26. 16585816

[71] Shanmugam J, Kumar M, GD Ravikumar S. Prevalence and determinants of anemia among adolescents in Coimbatore District, Tamil Nadu – a school based analytical cross-sectional study. Natl J Community Med. 2023;14(01):3–9.

[72] Sharma SK, Narain K, Devi KR, Mohapatra PK, Phukana RK, Mahanta J. Haemoglobinopathies - major associating determinants in prevalence of anaemia among adolescent girl students of Assam, India. WHO South-East Asia J Public Health. 2012;1(3):299–308. 28615556 10.4103/2224-3151.207026

[73] Naidu CS, Venela P, Ammika P, Kattula SR, Kokkiligadda SV, Deshmukh H. Factors influencing anaemia among adolescent girls from urban slums of Hyderabad-A Cross Sectional Cohort Study. Indian J Public Health Res Dev. 2014;5(3):16.

[74] Siva PM, Sobha A, Manjula VD. Prevalence of anaemia and its associated risk factors among adolescent girls of Central Kerala. J Clin Diagn Res. 2016;10(11):LC19–23. 28050409 10.7860/JCDR/2016/20939.8938 PMC5198362

[75] Srivastava S, Kumar P, Paul R, Debnath P. Effect of change in individual and household level characteristics on anemia prevalence among adolescent boys and girls in India. BMC Public Health. 2022;22(1):1478. 35922790 10.1186/s12889-022-13863-w PMC9351076

[76] Subramanian M, Malhotra S, Kant S, Goswami K, Perumal V, Kaloiya G. Prevalence of anemia among adolescent girls residing in Rural Haryana: a community-based cross-sectional study. Cureus. 2022;14(1): e21091. 35165551 10.7759/cureus.21091 PMC8830372

[77] Sulakshana B, Naik Vijaya A, Mallapur MD. A study of anaemia among adolescent girls in rural area of Belgaum district, Karnataka, south India. Indian J Public Health Res Dev. 2014;5(2):238–43.

[78] Toteja GS, Singh P, Dhillon BS, et al. Prevalence of anemia among pregnant women and adolescent girls in 16 districts of India. Food Nutr Bull. 2006;27(4):311–5. 17209473 10.1177/156482650602700405

[79] Verma K, Baniya GC. Prevalence, knowledge, and related factor of anemia among school-going adolescent girls in a remote area of western Rajasthan. J Family Med Prim Care. 2022;11(4):1474–81. 35516663 10.4103/jfmpc.jfmpc_1372_21 PMC9067232

[80] Verma M, Chhatwal J, Kaur G. Prevalence of anemia among urban school children of Punjab. Indian Pediatr. 1998;35(12):1181–6. 10216692

[81] Wangaskar SA, Sahu SK, Majella MG, Rajaa S. Prevalence of anaemia and compliance to weekly iron-folic acid supplementation programme amongst adolescents in selected schools of urban Puducherry. India Niger Postgrad Med J. 2021;28(1):44–50. 33642324 10.4103/npmj.npmj_336_20

[82] William RF, Balaji R, Logaraj M. Anaemia and associated factors among school going adolescent girls in Chidambaram, Tamilnadu - A cross sectional study. J Int Med Sci Acad. 2016;29(1):11–3.

[83] Bentley M, Griffiths P. The burden of anemia among women in India. Eur J Clin Nutr. 2003;57:52–60. 12548297 10.1038/sj.ejcn.1601504

[84] Bhardwaj A, Kumar D, Raina SK, Bansal P, Bhushan S, Chander V. Rapid assessment for coexistence of vitamin B12 and iron deficiency anemia among adolescent males and females in Northern Himalayan State of India. Anemia. 2013;2013:959605. 23970962 10.1155/2013/959605 PMC3736489

[85] Bharati P, Som S, Chakrabarty S, Bharati S, Pal M. Prevalence of anemia and its determinants among nonpregnant and pregnant women in India. Asia Pac J Public Health. 2008;20(4):347–59. 19124329 10.1177/1010539508322762

[86] Chowdhury TK, Roy SK. Prevalence of anaemia and associated factors among Oraon females of North 24 Parganas, West Bengal, India. Anthropol Rev. 2019;82(1):15–27.

[87] Dey S, Goswami S, Goswami M. Prevalence of anaemia in women of reproductive age in Meghalaya: A logistic regression analysis. Turk J Med Sci. 2010;40(5):783–9.

[88] Didzun O, De Neve JW, Awasthi A, et al. Anaemia among men in India: a nationally representative cross-sectional study. Lancet Glob Health. 2019;7(12):e1685–94. 31708149 10.1016/S2214-109X(19)30440-1

[89] Dudeja P, Tewari R, Singh A, Roy SD. Low prevalence of anaemia among the wives of serving personnel in a military station: A community-based study. Med J Armed Forces India. 2016;72(4):356–61. 27843183 10.1016/j.mjafi.2016.07.008 PMC5099445

[90] Finkelstein JL, Fothergill A, Johnson CB, et al. Anemia and vitamin B-12 and folate status in women of reproductive age in Southern India: estimating population-based risk of neural tube defects. Curr Dev Nutr. 2021;5(5):nzab069. 34027296 10.1093/cdn/nzab069 PMC8128722

[91] Ghosh P, Dasgupta A, Paul B, Roy S, Biswas A, Yadav A. A cross-sectional study on prevalence and determinants of anemia among women of reproductive age in a rural community of West Bengal. J Family Med Prim Care. 2020;9(11):5547–53. 33532393 10.4103/jfmpc.jfmpc_1209_20 PMC7842492

[92] Gupta VK, Maria AK, Kumar R, Bahia JS, Arora S. To study the prevalence of anaemia in young males and females with respect to the age, body mass index (BMI), activity profile and the socioeconomic status in rural Punjab. J Clin Diagnostic Res. 2011;5:1020–6.

[93] Haralkar SJ, Khandekar SV, Pore PD, Haralkar AS, Tapare VS, Rayate MV. Socio-demographic correlates of anaemia among married women in rural area of Maharashtra. Indian J Public Health Res Dev. 2013;4(3):107.

[94] Jana A, Chattopadhyay A, Saha UR. Identifying risk factors in explaining women’s anaemia in limited resource areas: evidence from West Bengal of India and Bangladesh. BMC Public Health. 2022;22(1):1433. 35897059 10.1186/s12889-022-13806-5 PMC9330636

[95] Jones AD, Hayter AK, Baker CP, et al. The co-occurrence of anemia and cardiometabolic disease risk demonstrates sex-specific sociodemographic patterning in an urbanizing rural region of southern India. Eur J Clin Nutr. 2016;70(3):364–72. 26508461 10.1038/ejcn.2015.177 PMC4874465

[96] Kamath R, Majeed JA, Chandrasekaran V, Pattanshetty SM. Prevalence of anemia among tribal women of reproductive age in Udupi Taluk. Karnataka J Family Med Prim Care. 2013;2(4):345–8. 26664839 10.4103/2249-4863.123881 PMC4649888

[97] Kandasamy K, Prasad A, Surendran A, Sebastian AC, Rajagopal SS, Ramanathan S. Epidemiological study of prevalence of anemia and associated risk factors in a rural community; a home-based screening. Asian J Pharm Clin Res. 2017;10(2):307–9.

[98] Kant S, Kumar R, Malhotra S, Kaur R, Haldar P. Prevalence and determinants of anemia among adult males in a rural area of Haryana, India. J Epidemiol Global Health. 2019;9(2):128–34. 10.2991/jegh.k.190513.001 PMC7310746 31241871

[99] Kishore S, Singh M, Jain B, Verma N, Gawande K, Kishore S, Aggarwal P, Verma SK. A study to assess prevalence of anaemia among beneficiaries of Anaemia Mukt Bharat Campaign in Uttarakhand. J Family Med Prim Care. 2020;9(3):1691–4. 32509673 10.4103/jfmpc.jfmpc_941_19 PMC7266259

[100] Kumar P, Sharma H, Patel KK. Prevalence and risk factors of anaemia among men: A study based on Empowered Action Group states. India Nutr Health. 2021;27(2):191–8. 33472523 10.1177/0260106020982348

[101] Kumar P, Sharma H, Sinha D. Socio-economic inequality in anaemia among men in India: a study based on cross-sectional data. BMC Public Health. 2021;21(1):1345. 34233633 10.1186/s12889-021-11393-5 PMC8265140

[102] Little M, Zivot C, Humphries S, Dodd W, Patel K, Dewey C. Burden and determinants of anemia in a rural population in South India: a cross-sectional study. Anemia. 2018;2018:7123976. 30112198 10.1155/2018/7123976 PMC6077670

[103] Malhotra P, Kumari S, Kumar R, Varma S. Prevalence of anemia in adult rural population of north India. J Assoc Physicians India. 2004;52:18–20. 15633712

[104] Mandal M, Mishra NP, Chatterjee P, Bhattacharyya S, Mondal KC, Acharya S. A longitudinal study on the prevalence of iron deficiency anemia in the multiethnic communities of Jhargram, West Bengal. Acta Biol Szeged. 2023;66(2):180–91.

[105] Osborn AJ, Muhammad GM, Ravishankar SL, Mathew AC. Prevalence and correlates of anemia among women in the reproductive age (15–49 years) in a rural area of Tamil Nadu: An exploratory study. J Educ Health Promot. 2021;10:355. 34761041 10.4103/jehp.jehp_1526_20 PMC8552273

[106] Panyang R, Teli AB, Saikia SP. Prevalence of anemia among the women of childbearing age belonging to the tea garden community of Assam, India: A community-based study. J Family Med Prim Care. 2018;7(4):734–8. 30234046 10.4103/jfmpc.jfmpc_274_17 PMC6131991

[107] Rao S, Joshi S, Bhide P, Puranik B, Kanade A. Social dimensions related to anaemia among women of childbearing age from rural India. Public Health Nutr. 2011;14(2):365–72. 20939942 10.1017/S1368980010002776

[108] Rohisha IK, Jose TT, Chakrabarty J. Prevalence of anemia among tribal women. J Family Med Prim Care. 2019;8(1):145–7. 30911496 10.4103/jfmpc.jfmpc_249_16 PMC6396609

[109] Seth RK, Khan S. Biosocial correlates of anemia in rural women of Bareilly. Uttar Pradesh Indian J Community Health. 2015;27(1):72–6.

[110] Shimrah C, Devi HS. Prevalence of anemia and associated risk factors among the lactating and nonpregnant-nonlactating Tangkhul women. Indian J Public Health. 2022;66(2):182–6. 35859502 10.4103/ijph.ijph_1633_21

[111] Shrinivasa BM, Philip RR, Krishnapali VK, Suraj A, Sreelakshmi PR. Prevalence of anaemia among tribal women of reproductive age-group in Wayanad district of Kerala. Int J Health Allied Sci. 2014;3:120–4.

[112] Siddiqui MZ, Goli S, Reja T, et al. Prevalence of anemia and its determinants among pregnant, lactating, and nonpregnant nonlactating women in India. SAGE Open. 2017;7(3):2158244017725555.

[113] Singh A, Ram S, Singh S, Tripathi P. Prevalence and determinants of anaemia among men in rural India: Evidence from a nationally representative survey. PLOS global public health. 2022;2(12): e0001159. 36962811 10.1371/journal.pgph.0001159 PMC10021440

[114] Singh B, Verma SP, Chauhan AS, Verma DP. Prevalence of anemia among reproductive-age females in the Tharu tribe of the Indo-Nepal border region. J Family Med Prim Care. 2022;11(6):2961–4. 36119223 10.4103/jfmpc.jfmpc_2055_21 PMC9480751

[115] Singh G, Singh K. Prevalence of anaemia in urban college going girl students. Biomedical Research (India). 2017;28(24):1040–2.

[116] Singh RK. Lifestyle behavior affecting prevalence of anemia among women in EAG states. India J Public Health. 2013;21:279–88.

[117] Sinha NK, Chattopadhyay JC, Das PK, Maiti S, Maiti K. Prevalence of anemia and its possible attributing factors in psychologically healthy women of reproductive ages in Midnapore (Jangalmahal-area), India. Indian J Community Health. 2013;25(3):226–32.

[118] Thankachan P, Muthayya S, Walczyk T, Kurpad AV, Hurrell RF. An analysis of the etiology of anemia and iron deficiency in young women of low socioeconomic status in Bangalore. India Food Nutr Bull. 2007;28(3):328–36. 17974366 10.1177/156482650702800309

[119] Verma R, Kharb M, Deswal S, Arora V, Kamboj R. Prevalence of anaemia among women of reproductive age group in a rural block of Northern India. Indian J Community Health. 2014;26(Supp 2):359–64.

[120] Agarwalla R, Saikia AM, Parashar M, Pathak R, Islam F. Assessment of prevalence of anemia in and its correlates among community-dwelling elderly of Assam, India: A cross-sectional study. Int J Nutr Pharmacol Neurol Dis. 2016;6(1):23–7.

[121] Debnath A, Rehman T, Ghosh T, Kaur A, Ahamed F. Prevalence of anemia among elderly population residing in an Urban Area of West Bengal: a community-based cross-sectional analytical study. Indian J Community Med. 2022;47(4):604–8. 36742972 10.4103/ijcm.ijcm_522_22 PMC9891053

[122] Gonmei Z, Dwivedi S, Toteja GS, Singh K, Vikram NK, Bansal PG. Anemia and vitamin b12 deficiency in elderly. Asian J Pharm Clin Res. 2018;11(1):402–4.

[123] Gupta S, Kumar R, Kalaivani M, Nongkynrih B, Kant S, Gupta SK. Underweight, overweight, and anemia among elderly persons in a rural area of Ballabgarh, Haryana. Indian J Community Med. 2021;46(3):511–4. 34759499 10.4103/ijcm.IJCM_688_20 PMC8575191

[124] Kaur M. Dietary intake, prevalence, and the effect of anemia on various morphophysiological variables of postmenopausal women of North India. J Midlife Health. 2018;9(2):72–8. 29962805 10.4103/jmh.JMH_20_18 PMC6006805

[125] Pathania A, Haldar P, Kant S, Gupta SK, Pandav CS, Bachani D. Prevalence of anemia among elderly persons residing in old age homes in national capital territory, Delhi, India. Indian J Public Health. 2019;63(4):288–92. 32189646 10.4103/ijph.IJPH_412_18

[126] Retnakumar C, Chacko M, Ramakrishnan D, George LS, Krishnapillai V. Prevalence of anemia and its association with dietary pattern among elderly population of urban slums in Kochi. J Family Med Prim Care. 2020;9(3):1533–7. 32509645 10.4103/jfmpc.jfmpc_1113_19 PMC7266240

[127] Singh T, Nagesh S, Ray TK. Magnitude and correlates of anemia in elderly women of a resettlement colony of Delhi. J Midlife Health. 2018;9(1):21–5. 29628724 10.4103/jmh.JMH_57_17 PMC5879843

[128] Agarwal KN, Agarwal DK, Sharma A, et al. Prevalence of anaemia in pregnant & lactating women in India. Indian J Med Res. 2006;124(2):173–84. 17015931

[129] Ahmad N, Kalakoti P, Bano R, Aarif SM. The prevalence of anaemia and associated factors in pregnant women in a rural Indian community. Australasian Medical J. 2010;3(5):276–80.

[130] Bala DV, Vyas S, Shukla A, Tiwari H, Bhatt G, Gupta K. Validity and reliability of haemoglobin colour scale and its comparison with clinical signs in diagnosing anaemia in pregnancy in Ahmedabad, India. East Mediterr Health J. 2012;18(7):749–54. 22891524 10.26719/2012.18.7.749

[131] Bone JN, Bellad M, Goudar S, et al. Anemia and adverse outcomes in pregnancy: subgroup analysis of the CLIP cluster-randomized trial in India. BMC Pregnancy Childbirth. 2022;22(1):407. 35562720 10.1186/s12884-022-04714-y PMC9101819

[132] Bora R, Sable C, Wolfson J, Boro K, Rao R. Prevalence of anemia in pregnant women and its effect on neonatal outcomes in Northeast India. J Matern Fetal Neonatal Med. 2014;27(9):887–91. 24041147 10.3109/14767058.2013.845161

[133] Corrêa G, Das M, Kovelamudi R, et al. High burden of malaria and anemia among tribal pregnant women in a chronic conflict corridor in India. Conflict Health. 2017;11:1–9. 28649273 10.1186/s13031-017-0113-1 PMC5477337

[134] Debnath A, Debbarma A, Debbarma SK, Bhattacharjya H. Proportion of anaemia and factors associated with it among the attendees of the antenatal clinic in a teaching institute of northeast India. J Family Med Prim Care. 2021;10(1):283–8. 34017741 10.4103/jfmpc.jfmpc_1499_20 PMC8132755

[135] Finkelstein JL, Kurpad AV, Bose B, Thomas T, Srinivasan K, Duggan C. Anaemia and iron deficiency in pregnancy and adverse perinatal outcomes in Southern India. Eur J Clin Nutr. 2020;74(1):112–25. 31296936 10.1038/s41430-019-0464-3 PMC10122513

[136] Gogoi I, Mahanta TG, Sarma R, Gogoi PP, Saikia H. Prevalence and socio-demographic factors affecting anaemia in pregnant women of Dibrugarh District, Assam, India. Indian J Community Health. 2016;28(2):202–7.

[137] Grover K, Kumar T, Doda A, et al. Prevalence of anaemia and its association with dietary habits among pregnant women in the urban area of Haryana. J Fam Med Prim Care. 2020;9(2):783–7. 10.4103/jfmpc.jfmpc_1062_19 PMC7114061 32318420

[138] Krupp K, Placek CD, Wilcox M, et al. Financial decision-making power is associated with moderate to severe anemia: A prospective cohort study among pregnant women in rural South India. Midwifery. 2018;61:15–21. 29522982 10.1016/j.midw.2018.02.014 PMC5916045

[139] Kumar V, Sunderam S, Haider S, Kashyap V. A study on status of anaemia in pregnant women attending urban health training centre, RIMS, Ranchi. Indian J Community Health. 2014;26(Supp 2):112–7.

[140] Mahashabde P, Arora VK, Sharma S, Shahjada A, Dabhi HM. Prevalence of anaemia and its socio-demographic determinants in pregnant women: a cross-sectional study in tertiary health care setup in central India. Natl J Commun Med. 2014;5:126–30.

[141] Mangla M, Singla D. Prevalence of anaemia among pregnant women in rural India: a longitudinal observational study. Int J Reprod Contracept Obstet Gynecol. 2016;5:3500–5.

[142] Mehrotra M, Yadav S, Deshpande A, Mehrotra H. A study of the prevalence of anemia and associated sociodemographic factors in pregnant women in Port Blair, Andaman and Nicobar Islands. J Fam Med Prim Care. 2018;7(6):1288–93. 10.4103/jfmpc.jfmpc_139_18 PMC6293883 30613513

[143] Nair M, Choudhury MK, Choudhury SS, et al. Association between maternal anaemia and pregnancy outcomes: a cohort study in Assam, India. BMJ Glob Health. 2016;1(1): e000026. 28588921 10.1136/bmjgh-2015-000026 PMC5321311

[144] Patel A, Prakash AA, Das PK, Gupta S, Pusdekar YV, Hibberd PL. Maternal anemia and underweight as determinants of pregnancy outcomes: cohort study in eastern rural Maharashtra, India. BMJ Open. 2018;8(8): e021623. 30093518 10.1136/bmjopen-2018-021623 PMC6089300

[145] Rajaratnam J, Abel R, Ganesan C, Jayaseelan SA. Maternal anaemia: a persistent problem in rural Tamil Nadu. Natl Med J India. 2000;13:242–5. 11190052

[146] Samuel TM, Thomas T, Finkelstein J, et al. Correlates of anaemia in pregnant urban South Indian women: a possible role of dietary intake of nutrients that inhibit iron absorption. Public Health Nutr. 2013;16(2):316–24. 22575487 10.1017/S136898001200119X PMC3713478

[147] Sharma JB, Soni D, Murthy NS, Malhotra M. Effect of dietary habits on prevalence of anemia in pregnant women of Delhi. J Obstet Gynaecol Res. 2003;29(2):73–8. 12755525 10.1046/j.1341-8076.2003.00079.x

[148] Siddiqui R, Mangi MM, Shah AA, Soomro RA, Memon KN. Major determinants of anemia in pregnant women residing in the urban slums of Taluka Qasimabad, district Hyderabad. Med Forum Mon. 2014;25(12):35–8.

[149] Singh P, Chaudhary V. Prevalence of anaemia and its socio demographic determinants among pregnant women in Bareilly district, Uttar Pradesh. Indian J Community Health. 2015;67(5):348–52.

[150] Sinha A, Adhikary M, Phukan JP, Kedia S, Sinha T. A study on anemia and its risk factors among pregnant women attending antenatal clinic of a rural medical college of West Bengal. J Family Med Prim Care. 2021;10(3):1327–31. 34041173 10.4103/jfmpc.jfmpc_1588_20 PMC8140236

[151] Suryanarayana R, Chandrappa M, Santhuram AN, Prathima S, Sheela SR. Prospective study on prevalence of anemia of pregnant women and its outcome: a community-based study. J Family Med Prim Care. 2017;6(4):739–43. 29564255 10.4103/jfmpc.jfmpc_33_17 PMC5848390

[152] Vemulapalli B, Rao K. Prevalence of anaemia among pregnant women of rural community in Vizianagaram, North Coastal Andhra Pradesh, India. Asian J Med Sci. 2013;5:21–5.

[153] Vindhya J, Nath A, Murthy GVS, et al. Prevalence and risk factors of anemia among pregnant women attending a public-sector hospital in Bangalore, South India. J Family Med Prim Care. 2019;8(1):37–43. 30911478 10.4103/jfmpc.jfmpc_265_18 PMC6396586

[154] Arora K, Bahadur A, Mishra D, Mundhra R. Assessment of anaemia and nutritional status of antenatal women attending a tertiary care hospital. J Family Med Prim Care. 2022;11(6):3238–44. 36119212 10.4103/jfmpc.jfmpc_2500_20 PMC9480653

[155] Nair MS, Raphael L, Chandran P. Prevalence of anaemia and associated factors among antenatal women in rural Kozhikode. Kerala J Family Med Prim Care. 2022;11(5):1851–7. 35800537 10.4103/jfmpc.jfmpc_1326_20 PMC9254825

[156] Noronha JA, Bhaduri A, Vinod Bhat H, Kamath A. Maternal risk factors and anaemia in pregnancy: a prospective retrospective cohort study. J Obstet Gynaecol. 2010;30(2):132–6. 20143970 10.3109/01443610903267457

[157] Saraswathi KS, Aljabri F, Shyamala R. Prevalence of anaemia among antenatal women in a Tertiary Care Hospital, South India. Der Pharm Lett. 2013;5(1):146–8.

[158] Yadav U, Singh TB, Chaubey L. Prevalence of anemia in antenatal women at first point-of-care visit to district combined hospital, Chakia, Uttar Pradesh, India. Med J Dr DY Patil Vidyapeeth. 2020;13(4):350–5.

[159] Bhagwan D, Kumar A, Rao CR, Kamath A. Prevalence of anaemia among postnatal mothers in coastal Karnataka. J Clin Diagn Res. 2016;10(1):LC17–20. 26894096 10.7860/JCDR/2016/14534.7086 PMC4740624

[160] Rakesh P, Gopichandran V, Jamkhandi D, Manjunath K, George K, Prasad J. Determinants of postpartum anemia among women from a rural population in southern India. Int J Womens Health. 2014;6:395–400. 24748821 10.2147/IJWH.S58355 PMC3990363

[161] Selvaraj R, Ramakrishnan J, Sahu SK, et al. High prevalence of anemia among postnatal mothers in Urban Puducherry: A community-based study. J Family Med Prim Care. 2019;8(8):2703–7. 31548960 10.4103/jfmpc.jfmpc_386_19 PMC6753800

[162] Kant S, Kaur R, Goel AD, Malhotra S, Haldar P, Kumar R. Anemia at the time of delivery and its association with pregnancy outcomes: A study from a secondary care hospital in Haryana, India. Indian J Public Health. 2018;62(4):315–8. 30539898 10.4103/ijph.IJPH_40_18

[163] Kumari S, Garg N, Kumar A, et al. Maternal and severe anaemia in delivering women is associated with risk of preterm and low birth weight: a cross sectional study from Jharkhand. India One Health. 2019;8:100098. 31485474 10.1016/j.onehlt.2019.100098 PMC6715890

[164] Manjula VD, Parameshwari P, Pothen L, Sobha A. Prevalence of anaemia among female undergraduate students of government medical college Kottayam, Kerala. Int J Med Health Sci. 2014;3(2):133–8.

[165] Rani NA, Arasegowda R, Mukherjee P, Dhananjay SY. Prevalence of nutritional deficiency anaemia and its impact on scholastic performance among undergraduate medical students. J Clin Diagn Res. 2017;11(3):BC21–3. 28511373 10.7860/JCDR/2017/25367.9597 PMC5427299

[166] Vibhute NA, Shah U, Belgaumi U, Kadashetti V, Bommanavar S, Kamate W. Prevalence and awareness of nutritional anemia among female medical students in Karad, Maharashtra, India: A cross-sectional study. J Family Med Prim Care. 2019;8(7):2369–72. 31463259 10.4103/jfmpc.jfmpc_353_19 PMC6691421

[167] Daniel RA, Kalaivani M, Kant S, Gupta S. Prevalence of anaemia among adolescent girls (10–19 years) in India: A systematic review and meta-analysis. Nat Med J India. 2023;36(4):233. 10.25259/NMJI_637_21 38692640

[168] Rakesh PS. Prevalence of anaemia in Kerala State, Southern India - a systematic review. J Clin Diagn Res. 2017;11(5):LE01–4. 28658816 10.7860/JCDR/2017/24681.9951 PMC5483718

[169] Daniel RA, Ahamed F, Mandal S, Lognathan V, Ghosh T, Ramaswamy G, Loganathan V. Prevalence of anemia among the elderly in India: evidence from a systematic review and meta-analysis of cross-sectional studies. Cureus. 2023;15(7):e42333. 10.7759/cureus.42333 PMC10443921 37614252

[170] Stevens GA, Finucane MM, De-Regil LM, et al. Global, regional, and national trends in haemoglobin concentration and prevalence of total and severe anaemia in children and pregnant and non-pregnant women for 1995–2011: a systematic analysis of population-representative data. Lancet Glob Health. 2013;1(1):e16-25. 25103581 10.1016/S2214-109X(13)70001-9 PMC4547326

[171] IIPS and ICF. National Family Health Survey (NFHS-5): 2019–21: India. Mumbai: International Institute of Population Sciences; 2022.

[172] World Health Organization. Anaemia. 2025. https://www.who.int/news-room/fact-sheets/detail/anaemia.

