# Supplementary material for: Prevalence of anemia in India: a systematic review, meta-analysis and geospatial analysis
Source: BMC Public Health. 2025 Apr 4;25:1270. doi: 10.1186/s12889-025-22439-3 (PMC11969930; doi:10.1186/s12889-025-22439-3)
Supplement: Supplementary file 1 — Supplementary Material 1. [file 12889_2025_22439_MOESM1_ESM.docx]

**Supplementary Information**

**Full search terms for one database**

**PubMed:** ((((((((prevalence) OR (proportion)) OR (incidence)) OR (frequency)) OR (prevalent)) OR (frequence)) OR (occurrence)) AND ((((((anaemia) OR (anemia)) OR (bloodlessness)) OR (iron-deficiency anaemia)) OR (iron-poor blood)) OR (anaemic))) AND ((((((((((((((((((((((((((((((((((((((((((((((((((((India) OR (Bharat)) OR (Hindustan)) OR (southAsia)) OR (Indian subcontinent)) OR (Kerala)) OR (Karnataka)) OR (TamilNadu)) OR (AndhraPradesh)) OR (Arunachal Pradesh)) OR (Assam)) OR (Bihar)) OR (Chhattisgarh)) OR (Goa)) OR (Gujarat)) OR (Haryana)) OR (HimachalPradesh)) OR (Jharkhand)) OR (Madhya Pradesh)) OR (Maharashtra)) OR (Manipur)) OR (Meghalaya)) OR (Mizoram)) OR (Nagaland)) OR (Odisha)) OR (Punjab)) OR (Rajasthan)) OR (Sikkim)) OR (Telangana)) OR (Tripura)) OR (Uttar Pradesh)) OR (Uttarakhand)) OR (West Bengal)) OR (Jammu)) OR (kashmir)) OR (ladakh)) OR (pondicherry)) OR (delhi)) OR (ANDAMAN)) OR (NICOBAR ISLANDS)) OR (CHANDIGARH)) OR (orissa)) OR (DADRA)) OR (NAGARHAVELI)) OR (DAMAN)) OR (DIU)) OR (north india)) OR (southindia)) OR (northeastindia)) OR (central)) OR (eastindia)) OR (westindia))

### Supplementary Table 1: Characteristics of included studies

| **Study ID** | **Year of**  **publication** | **Year of conduct** | **Region** | **n** | **p** | **SE** | **Gender** | **NOS-Quality Score** | **Category** |
| --- | --- | --- | --- | --- | --- | --- | --- | --- | --- |
| Gosdin L et al., 2018 | 2018 | - | Bihar | 5664 | 0.69 | 0.0062 | female | 8 | Toddlers |
| Kalhan M et al., 2022 | 2022 | - | Haryana | 170 | 0.62 | 0.0372 | both | 8 |  |
| Kapoor D et al., 2002 | 2002 | 1997 | Delhi | 545 | 0.64 | 0.0206 | both | 6 |  |
| Krishnaswamy S et al., 2017 | 2007 | 2014 | Chandigarh | 296 | 0.05 | 0.0122 | both | 4 |  |
| Kumar T et al., 2014 | 2014 | 2011 | Delhi | 1000 | 0.70 | 0.0145 | both | 8 |  |
| Nadar, S. et al., 2016 | 2016 | 2016 | Tamil Nadu | 100 | 0.7 | 0.0458 | both | 4 |  |
|  | 2016 | 2016 | Tamil Nadu | 58 | 0.71 | 0.0597 | male | 4 |  |
|  | 2016 | 2016 | Tamil Nadu | 42 | 0.69 | 0.0712 | female | 4 |  |
| Nair KM et al., 2016 | 2016 | 2016 | Telangana | 512 | 0.67 | 0.0208 | both | 7 |  |
| Onyeneho NG et al., 2019 | 2019 | - | India | 112714 | 0.59 | 0.0015 | both | 8 |  |
| Pasricha SR et al., 2010 | 2010 | 2008 | Karnataka | 401 | 0.75 | 0.0215 | both | 8 |  |
| Saba F et al., 2014 | 2014 | 2012 | Karnataka | 882 | 0.73 | 0.0150 | both | 6 |  |
| Stiller CK et al., 2020 | 2020 | 2015 | West Bengal | 307 | 0.94 | 0.0138 | both | 8 |  |
|  | 2020 | 2015 | West Bengal | 164 | 0.94 | 0.0187 | male | 8 |  |
|  | 2020 | 2015 | West Bengal | 143 | 0.94 | 0.0203 | female | 8 |  |
| Arlappa N. et al., 2010 (a) | 2010 | 2003 | West Bengal | 437 | 0.81 | 0.0187 | both | 9 | Pre-school children |
|  | 2010 | 2003 | West Bengal | 218 | 0.80 | 0.0272 | male | 9 |  |
|  | 2010 | 2003 | West Bengal | 219 | 0.83 | 0.0256 | female | 9 |  |
| Arlappa N. et al., 2012 (b) | 2012 | 2003 | Maharashtra | 404 | 0.59 | 0.0245 | both | 9 |  |
|  | 2012 | 2003 | Maharashtra | 243 | 0.57 | 0.0318 | male | 9 |  |
|  | 2012 | 2003 | Maharashtra | 161 | 0.63 | 0.0381 | female | 9 |  |
| Arlappa N. et al., 2014 (c) | 2014 | - | Maharashtra | 404 | 0.59 | 0.0245 | both | 9 |  |
| Awasthi S et al., 2003 | 2003 | 2002 | Uttar Pradesh | 1200 | 0.70 | 0.0132 | both | 6 |  |
| Behera S et al., 2016 | 2016 | - | Orissa | 101 | 0.49 | 0.0497 | both | 5 |  |
|  | 2016 | - | Orissa | 63 | 0.48 | 0.0629 | male | 5 |  |
|  | 2016 | - | Orissa | 38 | 0.50 | 0.0811 | female | 5 |  |
| Bhattacharyya K et al., 2010 | 2010 | - | West Bengal | 188 | 0.46 | 0.0363 | both | 5 |  |
|  | 2010 | - | West Bengal | 74 | 0.51 | 0.0581 | male | 5 |  |
|  | 2010 | - | West Bengal | 114 | 0.42 | 0.0462 | female | 5 |  |
| George KA et al., 2000 | 2000 | 1998 | Kerala | 3633 | 0.11 | 0.0053 | both | 7 |  |
| Goswmai S et al., 2015 | 2015 |  | India | 40885 | 0.70 | 0.0023 | both | 8 |  |
| Gupta S. et al., 2017(b) | 2017 | 2010 | Jammu & Kashmir | 89 | 0.60 | 0.0519 | both | 5 |  |
| Meshram II et al., 2020 (a) | 2020 | - | Nagaland | 227 | 0.27 | 0.0293 | both | 7 |  |
|  | 2020 | - | Nagaland | 123 | 0.30 | 0.0413 | male | 7 |  |
|  | 2020 | - | Nagaland | 104 | 0.23 | 0.0411 | female | 7 |  |
| Meshram II et al., 2021 (b) | 2021 | - | Meghalaya | 632 | 0.68 | 0.0186 | both | 7 |  |
| Mishra N. et al., 2016 | 2016 | - | Uttar Pradesh | 4556 | 0.74 | 0.0065 | both | 7 |  |
| Nair KM et al., 2016 | 2016 | 2016 | Telangana | 321 | 0.48 | 0.0279 | both | 7 |  |
| Panchal SS et al., 2022 | 2022 | 2018 | Gujarat | 300 | 0.88 | 0.0190 | both | 8 |  |
|  | 2022 | 2018 | Gujarat | 149 | 0.89 | 0.0261 | male | 8 |  |
|  | 2022 | 2018 | Gujarat | 151 | 0.87 | 0.0275 | female | 8 |  |
| Philip RR et al., 2015 | 2015 | 2010 | Kerala | 438 | 0.96 | 0.0097 | both | 7 |  |
| Sarna A et al., 2020 | 2020 | 2018 | India | 11233 | 0.41 | 0.0046 | both | 6 |  |
| Sharma U et al., 2019 | 2019 | 2016 | Uttar Pradesh | 365 | 0.93 | 0.0134 | both | 9 |  |
|  | 2019 | 2016 | Uttar Pradesh | 151 | 0.94 | 0.0193 | male | 9 |  |
|  | 2019 | 2016 | Uttar Pradesh | 214 | 0.92 | 0.0184 | female | 9 |  |
| Singh RK et al., 2014(b) | 2014 | - | India | 16065 | 0.70 | 0.0036 | both | 7 |  |
| Sudhagandhi B. et al., 2012 | 2012 | - | Tamil Nadu | 460 | 0.75 | 0.0201 | both | 5 |  |
|  | 2012 | - | Tamil Nadu | 220 | 0.79 | 0.0274 | male | 5 |  |
|  | 2012 | - | Tamil Nadu | 240 | 0.71 | 0.0293 | female | 5 |  |
| Ahankari AS et al., 2017 | 2017 | *-* | Maharashtra | 1010 | 0.87 | 0.0106 | female | 8 | School children |
| Arlappa N. et al., 2014(c) | 2014 | - | Maharashtra | 833 | 0.61 | 0.0169 | female | 9 |  |
| Banerjee M et al., 2022 | 2022 | *-* | Jammu & Kashmir | 1675 | 0.34 | 0.0116 | both | 5 |  |
|  | 2022 | *-* | Jammu & Kashmir | 1014 | 0.30 | 0.0143 | male | 5 |  |
|  | 2022 | *-* | Jammu & Kashmir | 661 | 0.44 | 0.0193 | female | 5 |  |
| Basu S et al., 2005 | 2005 | 2002 | Chandigarh | 1120 | 0.16 | 0.0110 | both | 7 |  |
|  | 2005 | 2002 | Chandigarh | 530 | 0.08 | 0.0116 | male | 7 |  |
|  | 2005 | 2002 | Chandigarh | 590 | 0.24 | 0.0176 | female | 7 |  |
| Behera S et al., 2016 | 2016 | *-* | Orissa | 212 | 0.69 | 0.0318 | both | 5 |  |
|  | 2016 | *-* | Orissa | 85 | 0.73 | 0.0482 | male | 5 |  |
|  | 2016 | *-* | Orissa | 127 | 0.66 | 0.0420 | female | 5 |  |
| Bharati P. et al.,2009(b) | 2009 | *-* | India | 177670 | 0.90 | 0.0007 | female | 5 |  |
| Bhatia V. et al., 2020 | 2020 | *-* | Orissa | 800 | 0.68 | 0.0165 | female | 8 |  |
| Biradar S.S. et al., 2012 | 2012 | 2008 | Karnataka | 840 | 0.41 | 0.0170 | female | 7 |  |
| Bulliyy G et al., 2007 | 2007 | *-* | Orissa | 1937 | 0.97 | 0.0042 | female | 9 |  |
| Chandrakumari AS et al., 2019 | 2019 | *-* | Tamil Nadu | 225 | 0.49 | 0.0333 | female | 7 |  |
| Chaudhary SM et al., 2008 | 2008 | *-* | Maharashtra | 296 | 0.35 | 0.0277 | female | 9 |  |
| Chauhan S et al., 2022 | 2022 | 2016 | Bihar | 4770 | 0.20 | 0.0058 | female | 6 |  |
|  | 2022 | 2016 | Bihar | 1900 | 0.09 | 0.0065 | male | 6 |  |
|  | 2022 | 2016 | Uttar Pradesh | 9855 | 0.20 | 0.0040 | female | 6 |  |
|  | 2022 | 2016 | Uttar Pradesh | 4069 | 0.09 | 0.0044 | male | 6 |  |
| Gopalakrishnan S et al., 2018 | 2018 | 2017 | Tamil Nadu | 250 | 0.85 | 0.0227 | female | 8 |  |
| Goyle A Jr et al., 2009 | 2009 | *-* | Rajasthan | 109 | 0.96 | 0.0181 | female | 4 |  |
| Gunjal Sandeep, S. et al., 2012 | 2012 | 2009 | Maharashtra | 908 | 0.90 | 0.0101 | both | 8 |  |
|  | 2012 | 2009 | Maharashtra | 463 | 0.88 | 0.0153 | male | 8 |  |
|  | 2012 | 2009 | Maharashtra | 445 | 0.92 | 0.0129 | female | 8 |  |
| Gupta S et al., 2012 (a) | 2012 | 2009 | West Bengal | 172 | 0.80 | 0.0304 | both | 6 |  |
| Gupta S. et al., 2017 (b) | 2017 | 2010 | Jammu & Kashmir | 214 | 0.50 | 0.0342 | both | 5 |  |
| Jagadish Kumar K. et al., 2017 | 2017 | *-* | Karnataka | 204 | 0.53 | 0.0350 | both | 6 |  |
|  | 2017 | *-* | Karnataka | 158 | 0.53 | 0.0397 | male | 6 |  |
|  | 2017 | *-* | Karnataka | 46 | 0.52 | 0.0736 | female | 6 |  |
| Jain T. et al., 2011 | 2011 | *-* | Uttar Pradesh | 400 | 0.43 | 0.0247 | male | 8 |  |
| Kamble BD et al., 2021 | 2021 | *-* | Delhi | 203 | 0.59 | 0.0345 | female | 7 |  |
| Kumar et al., 2023 | 2023 | 2016 | Andhra Pradesh | 384 | 0.28 | 0.0229 | male | 4 |  |
| Kumari R et al., 2017 | 2017 | *-* | Bihar | 200 | 0.50 | 0.0354 | female | 4 |  |
| Mahanta T.G. et al., 2015 | 2015 | *-* | Assam | 802 | 0.96 | 0.0067 | female | 9 |  |
| Manjula AA et al., 2003 | 2003 | 2003 | Kerala | 862 | 0.44 | 0.0169 | both | 4 |  |
| Muthayya S et al., 2007 | 2007 | 2006 | Karnataka | 2030 | 0.14 | 0.0076 | both | 5 |  |
| Nair et al., 2023 | 2023 | *-* | Maharashtra | 420 | 0.66 | 0.0232 | female | 9 |  |
| Prabhakar SCJ et al., 2009 | 2009 | *-* | Karnataka | 175 | 0.78 | 0.0315 | both | 5 |  |
| Rahman MHU et al., 2020 | 2020 | *-* | India | 14664 | 0.23 | 0.0035 | both | 9 |  |
| Rai RK et al., 2023 | 2023 | 2016 | Bihar | 696 | 0.60 | 0.0186 | female | 7 |  |
|  | 2023 | 2019 | Bihar | 696 | 0.74 | 0.0167 | female | 7 |  |
|  | 2023 | 2016 | Bihar | 909 | 0.29 | 0.0150 | male | 7 |  |
|  | 2023 | 2019 | Bihar | 909 | 0.31 | 0.0153 | male | 7 |  |
|  | 2023 | 2016 | Uttar Pradesh | 796 | 0.57 | 0.0176 | female | 7 |  |
|  | 2023 | 2019 | Uttar Pradesh | 796 | 0.59 | 0.0175 | female | 7 |  |
|  | 2023 | 2016 | Uttar Pradesh | 878 | 0.37 | 0.0163 | male | 7 |  |
|  | 2023 | 2019 | Uttar Pradesh | 878 | 0.32 | 0.0158 | male | 7 |  |
| Rakesh PS et al., 2019 | 2019 | *-* | Kerala | 880 | 0.44 | 0.0167 | both | 10 |  |
|  | 2019 | *-* | Kerala | 440 | 0.40 | 0.0233 | male | 10 |  |
|  | 2019 | *-* | Kerala | 440 | 0.48 | 0.0238 | female | 10 |  |
| Rakesh SR et al., 2014 | 2014 | 2012 | Kerala | 4813 | 0.72 | 0.0065 | both | 6 |  |
| Ramesh Masthi NR et al., 2012 | 2012 | 2012 | Karnataka | 323 | 0.09 | 0.0157 | both | 5 |  |
|  | 2012 | 2012 | Karnataka | 147 | 0.08 | 0.0217 | male | 5 |  |
|  | 2012 | 2012 | Karnataka | 176 | 0.10 | 0.0223 | female | 5 |  |
| S RP et al., 2015 | 2015 | 2014 | Kerala | 3200 | 0.31 | 0.0082 | both | 9 |  |
|  | 2015 | 2014 | Kerala | 1600 | 0.32 | 0.0116 | male | 9 |  |
|  | 2015 | 2014 | Kerala | 1600 | 0.31 | 0.0116 | female | 9 |  |
| Sahoo J et al., 2021 | 2021 | 2019 | Orissa | 953 | 0.46 | 0.0161 | both | 7 |  |
| Sarna A et al., 2020 | 2020 | 2018 | India | 14300 | 0.28 | 0.0038 | both | 6 |  |
|  | 2020 | 2018 | India | 14664 | 0.23 | 0.0035 | both | 6 |  |
| Sen A et al., 2006 | 2006 | *-* | Gujarat | 322 | 0.67 | 0.0262 | female | 5 |  |
| Shanmugam J et al., 2023 | 2023 | 2019 | Tamil Nadu | 2263 | 0.89 | 0.0067 | both | 8 |  |
|  | 2023 | 2019 | Tamil Nadu | 1073 | 0.50 | 0.0153 | female | 8 |  |
|  | 2023 | 2019 | Tamil Nadu | 1190 | 0.50 | 0.0145 | male | 8 |  |
| Sharma SK et al., 2012 | 2012 | *-* | Assam | 4457 | 0.72 | 0.0068 | female | 7 |  |
| Simhachalam Naidu C.H. et al., 2014 | 2014 | 2010 | Telangana | 300 | 0.79 | 0.0234 | female | 6 |  |
| Siva PM et al., 2016 | 2016 | 2014 | Kerala | 257 | 0.21 | 0.0254 | female | 9 |  |
| Srivastava S et al., 2022 | 2022 | 2016 | Bihar | 2581 | 0.59 | 0.0097 | female | 6 |  |
|  | 2022 | 2019 | Bihar | 2581 | 0.63 | 0.0095 | female | 6 |  |
|  | 2022 | 2016 | Bihar | 1465 | 0.33 | 0.0123 | male | 6 |  |
|  | 2022 | 2019 | Bihar | 1465 | 0.31 | 0.0120 | male | 6 |  |
|  | 2022 | 2016 | Uttar Pradesh | 3393 | 0.59 | 0.0084 | female | 6 |  |
|  | 2022 | 2019 | Uttar Pradesh | 3393 | 0.63 | 0.0083 | female | 6 |  |
|  | 2022 | 2016 | Uttar Pradesh | 2571 | 0.33 | 0.0093 | male | 6 |  |
|  | 2022 | 2019 | Uttar Pradesh | 2571 | 0.31 | 0.0091 | male | 6 |  |
| Subramanian M et al., 2022 | 2022 | *-* | Haryana | 272 | 0.72 | 0.0273 | female | 9 |  |
| Sulakshana B. et al., 2014 | 2014 | *-* | Karnataka | 400 | 0.75 | 0.0217 | female | 7 |  |
| Toteja GS et al., 2006 | 2006 | *-* | India | 4337 | 0.90 | 0.0045 | female | 5 |  |
| Verma K et al., 2022 | 2022 | 2020 | Rajasthan | 625 | 0.56 | 0.0198 | female | 8 |  |
| Verma M et al., 1998 | 1998 | *-* | Punjab | 2000 | 0.52 | 0.0112 | both | 5 |  |
| Wangaskar SA et al., 2021 | 2021 | 2019 | Pondicherry | 458 | 0.63 | 0.0226 | both | 9 |  |
|  | 2021 | 2019 | Pondicherry | 346 | 0.62 | 0.0261 | female | 9 |  |
|  | 2021 | 2019 | Pondicherry | 112 | 0.65 | 0.0450 | male | 9 |  |
| William R.F. et al., 2016 | 2016 | *-* | Tamil Nadu | 204 | 0.61 | 0.0341 | female | 8 |  |
| Bentley M.E. et al., 2003 | 2003 | 1999 | Andhra Pradesh | 4032 | 0.50 | 0.0079 | female | 8 | Adults |
| Bharati P. et al., 2008(a) | 2008 | - | India | 72660 | 0.50 | 0.0019 | female | 8 |  |
| Bhardwaj A et al., 2013 | 2013 | 2010 | Himachal Pradesh | 539 | 0.97 | 0.0077 | female | 6 |  |
|  | 2013 | 2010 | Himachal Pradesh | 346 | 0.87 | 0.0180 | male | 6 |  |
| Bhatia V. et al., 2020 | 2020 | *-* | Orissa | 800 | 0.71 | 0.0160 | female | 8 |  |
| Chowdhury T.K. et al., 2019 | 2019 | *-* | West Bengal | 309 | 0.80 | 0.0228 | female | 7 |  |
| Dey S. et al., 2010 | 2010 | *-* | Meghalaya | 3934 | 0.50 | 0.0080 | female | 7 |  |
| Didzun O et al., 2019 | 2019 | 2016 | India | 106298 | 0.23 | 0.0013 | male | 9 |  |
| Dudeja P et al., 2016 | 2016 | - | India | 575 | 0.14 | 0.0144 | female | 6 |  |
| Finkelstein JL et al., 2021(b) | 2021 | - | Andhra Pradesh | 980 | 0.42 | 0.0154 | female | 6 |  |
| Ghosh P et al., 2020 | 2020 | 2019 | West Bengal | 120 | 0.71 | 0.0415 | female | 8 |  |
| Gupta V.K. et al., 2011 | 2011 | *-* | Punjab | 3099 | 0.90 | 0.0055 | female | 4 |  |
|  | 2011 | *-* | Punjab | 1221 | 0.90 | 0.0086 | male | 4 |  |
| Haralkar S.J et al., 2013 | 2013 | 2004 | Maharashtra | 529 | 0.28 | 0.0194 | female | 5 |  |
| Jana A et al., 2022 | 2022 | *-* | West Bengal | 15756 | 0.64 | 0.0038 | female | 8 |  |
| Jones AD et. al., 2016 | 2016 | 2012 | Andhra Pradesh | 2895 | 0.40 | 0.0091 | female | 5 |  |
|  | 2016 | 2012 | Andhra Pradesh | 3322 | 0.10 | 0.0052 | male | 5 |  |
| Kamath R et al., 2013 | 2013 | *-* | Karnataka | 170 | 0.56 | 0.0381 | female | 5 |  |
| Kandasamy K. et al., 2017 | 2017 | *-* | Tamil Nadu | 200 | 0.73 | 0.0314 | both | 5 |  |
|  | 2017 | *-* | Tamil Nadu | 116 | 0.47 | 0.0463 | female | 5 |  |
|  | 2017 | *-* | Tamil Nadu | 84 | 0.26 | 0.0479 | male | 5 |  |
| Kant S et al., 2019 | 2019 | 2016 | Haryana | 1226 | 0.28 | 0.0128 | male | 10 |  |
| Kishore S et al., 2020 | 2020 | 2018 | Uttarakhand | 5776 | 0.53 | 0.0066 | both | 5 |  |
|  | 2020 | 2018 | Uttarakhand | 4942 | 0.55 | 0.0071 | female | 5 |  |
|  | 2020 | 2018 | Uttarakhand | 834 | 0.45 | 0.0172 | male | 5 |  |
| Kumar P et al., 2021(a) | 2021 | *-* | India | 108261 | 0.23 | 0.0001 | male | 5 |  |
| Kumar P et al., 2021(b) | 2021 | *-* | India | 112122 | 0.23 | 0.0013 | male | 8 |  |
| Little M et al., 2018 | 2018 | *-* | Tamil Nadu | 412 | 0.57 | 0.0244 | female | 8 |  |
|  | 2018 | *-* | Tamil Nadu | 341 | 0.35 | 0.0259 | male | 8 |  |
| Malhotra P et al., 2004 | 2004 | 1995 | Haryana | 215 | 0.48 | 0.0341 | both | 4 |  |
|  | 2004 | 1995 | Haryana | 136 | 0.5 | 0.0429 | female | 4 |  |
|  | 2004 | 1995 | Haryana | 79 | 0.44 | 0.0559 | male | 4 |  |
| Mandal et al., 2022 | 2022 | 2022 | West Bengal | 910 | 0.67 | 0.0155 | both | 4 |  |
|  | 2022 | 2022 | West Bengal | 529 | 0.43 | 0.0215 | female | 4 |  |
|  | 2022 | 2022 | West Bengal | 381 | 0.25 | 0.0221 | male | 4 |  |
| Meshram II et al., 2020(a) | 2020 | - | Nagaland | 274 | 0.40 | 0.0296 | female | 7 |  |
| Osborn AJ et al., 2021 | 2021 | 2016 | Tamil Nadu | 426 | 0.65 | 0.0231 | female | 9 |  |
| Panyang R et al., 2018 | 2018 | 2016 | Assam | 770 | 0.08 | 0.0100 | female | 6 |  |
| Rao S et al., 2011 | 2011 | *-* | Maharashtra | 418 | 0.77 | 0.0206 | female | 5 |  |
| Rohisha IK et al., 2019 | 2019 | *-* | Kerala | 445 | 0.89 | 0.0148 | female | 7 |  |
| Seth R.K. et al., 2015 | 2015 | *-* | Uttar Pradesh | 435 | 0.49 | 0.0240 | female | 8 |  |
| Shimrah C et al., 2022 | 2022 | *-* | Manipur | 250 | 0.57 | 0.0313 | female | 6 |  |
| Shrinivasa BM et al., 2014 | 2014 | 2013 | Kerala | 347 | 0.97 | 0.0099 | female | 7 |  |
| Siddiqui M.Z et al., 2017 | 2017 | - | India | 97418 | 0.53 | 0.0016 | female | 7 |  |
| Singh A et al., 2022 | 2022 | - | India | 61481 | 0.28 | 0.0018 | male | 9 |  |
| Singh B et al., 2022 | 2022 | 2019 | Uttar Pradesh | 440 | 0.64 | 0.0228 | female | 4 |  |
| Singh G. et al., 2017 | 2017 | *-* | Maharashtra | 122 | 0.72 | 0.0406 | female | 6 |  |
| Singh R.K. 2013 (a) | 2013 | *-* | Bihar | 3323 | 0.68 | 0.0081 | female | 7 |  |
|  | 2013 | *-* | Chhattisgarh | 3573 | 0.57 | 0.0083 | female | 7 |  |
|  | 2013 | *-* | Jharkhand | 2573 | 0.69 | 0.0091 | female | 7 |  |
|  | 2013 | *-* | Madhya Pradesh | 6048 | 0.56 | 0.0064 | female | 7 |  |
|  | 2013 | *-* | Orissa | 4152 | 0.61 | 0.0076 | female | 7 |  |
|  | 2013 | *-* | Rajasthan | 3643 | 0.53 | 0.0083 | female | 7 |  |
|  | 2013 | *-* | Uttar Pradesh | 10175 | 0.50 | 0.0050 | female | 7 |  |
|  | 2013 | *-* | Uttarakhand | 2693 | 0.55 | 0.0096 | female | 7 |  |
| Sinha N.K. et al., 2013 | 2013 | 2011 | West Bengal | 241 | 0.70 | 0.0296 | female | 7 |  |
| Thankachan P et al., 2007 | 2007 | *-* | Karnataka | 100 | 0.39 | 0.0488 | female | 5 |  |
| Verma R. et al., 2015 | 2015 | 2014 | Haryana | 8590 | 0.49 | 0.0054 | female | 7 |  |
| Agarwalla R. et al., 2016 | 2016 | 2012 | Assam | 330 | 0.46 | 0.0274 | both | 8 | Elderly Persons |
|  | 2016 | 2012 | Assam | 139 | 0.30 | 0.0389 | male | 8 |  |
|  | 2016 | 2012 | Assam | 191 | 0.57 | 0.0358 | female | 8 |  |
| Debnath A et al., 2022 | 2022 | 2021 | West Bengal | 457 | 0.65 | 0.0223 | both | 10 |  |
| Gonmei Z et al., 2018 | 2018 | *-* | Delhi | 116 | 0.58 | 0.0459 | both | 4 |  |
|  | 2018 | *-* | Delhi | 61 | 0.54 | 0.0638 | male | 4 |  |
|  | 2018 | *-* | Delhi | 55 | 0.62 | 0.0655 | female | 4 |  |
| Gupta S et al., 2021(c) | 2021 | 2018 | Haryana | 382 | 0.36 | 0.0246 | both | 9 |  |
|  | 2021 | 2018 | Haryana | 160 | 0.33 | 0.0372 | male | 9 |  |
|  | 2021 | 2018 | Haryana | 222 | 0.39 | 0.0327 | female | 9 |  |
| Kaur M 2018 | 2018 | 2010 | Chandigarh | 250 | 0.85 | 0.0225 | female | 6 |  |
|  | 2018 | 2010 | Haryana | 250 | 0.85 | 0.0225 | female | 6 |  |
|  | 2018 | 2010 | Punjab | 250 | 0.85 | 0.0225 | female | 6 |  |
| Pathania A et al., 2019 | 2019 | 2015 | Delhi | 335 | 0.69 | 0.0253 | both | 9 |  |
|  | 2019 | 2015 | Delhi | 129 | 0.65 | 0.0420 | male | 9 |  |
|  | 2019 | 2015 | Delhi | 206 | 0.71 | 0.0316 | female | 9 |  |
| Retnakumar C et al., 2020 | 2020 | 2018 | Kerala | 165 | 0.61 | 0.0380 | both | 9 |  |
|  | 2020 | 2018 | Kerala | 55 | 0.49 | 0.0674 | male | 9 |  |
|  | 2020 | 2018 | Kerala | 110 | 0.66 | 0.0452 | female | 9 |  |
| Singh T et al., 2018 | 2018 | 2013 | Delhi | 512 | 0.80 | 0.0177 | female | 9 |  |
| Agarwal KN et al., 2006 | 2006 | 2003 | Assam | 132 | 0.94 | 0.0208 | female | 6 | Pregnant women |
|  | 2006 | 2003 | Haryana | 188 | 0.91 | 0.0209 | female | 6 |  |
|  | 2006 | 2003 | Himachal Pradesh | 94 | 0.68 | 0.0481 | female | 6 |  |
|  | 2006 | 2003 | Kerala | 244 | 0.58 | 0.0316 | female | 6 |  |
|  | 2006 | 2003 | Madhya Pradesh | 125 | 0.97 | 0.0157 | female | 6 |  |
|  | 2006 | 2003 | Orissa | 164 | 0.97 | 0.0133 | female | 6 |  |
|  | 2006 | 2003 | Tamil Nadu | 201 | 0.92 | 0.0197 | female | 6 |  |
| Ahmad N. et al., 2010 | 2010 | - | Maharashtra | 310 | 0.75 | 0.0246 | female | 7 |  |
| Arlappa N. et al., 2014(c) | 2014 | - | Maharashtra | 386 | 0.76 | 0.0218 | female | 9 |  |
| Bala DV et al., 2012 | 2012 | 2010 | Gujarat | 129 | 0.70 | 0.0404 | female | 7 |  |
| Bharati P. et al., 2008(a) | 2008 | - | India | 5619 | 0.48 | 0.0067 | female | 8 |  |
| Bhatia V. et al., 2020 | 2020 | - | Orissa | 786 | 0.69 | 0.0165 | female | 8 |  |
| Bone JN et al., 2022 | 2022 | - | Karnataka | 11085 | 0.89 | 0.0030 | female | 7 |  |
| Bora R et al., 2014 | 2014 | - | Assam | 470 | 0.90 | 0.0141 | female | 5 |  |
| Corrêa G et al., 2017 | 2017 | 2015 | Andhra Pradesh | 575 | 0.92 | 0.0111 | female | 7 |  |
|  | 2017 | 2015 | Chhattisgarh | 575 | 0.92 | 0.0111 | female | 7 |  |
|  | 2017 | 2015 | Telangana | 575 | 0.92 | 0.0111 | female | 7 |  |
| Debnath A et al., 2021 | 2021 | 2019 | Tripura | 200 | 0.60 | 0.0346 | female | 6 |  |
| Finkelstein JL et al., 2020(a) | 2020 | - | Karnataka | 366 | 0.30 | 0.0240 | female | 8 |  |
| Gogoi I. et al., 2016 | 2016 | 2016 | Assam | 290 | 0.73 | 0.0260 | female | 9 |  |
| Grover K et al., 2020 | 2020 | 2018 | Haryana | 408 | 0.85 | 0.0175 | female | 8 |  |
| Kishore S et al., 2020 | 2020 | - | Uttarakhand | 164 | 0.34 | 0.0369 | female | 5 |  |
| Krupp K et al., 2018 | 2018 | 2012 | Karnataka | 1654 | 0.67 | 0.0116 | female | 7 |  |
| Kumar V et al., 2014 | 2014 | 2014 | Jharkhand | 149 | 0.66 | 0.0387 | female | 9 |  |
| Mahashabde P et al., 2014 | 2014 | 2013 | Madhya Pradesh | 300 | 0.63 | 0.0279 | female | 8 |  |
| Mangla M et al., 2016 | 2016 | 2015 | Haryana | 850 | 0.98 | 0.0048 | female | 5 |  |
| Mehrotra M et al., 2018 | 2018 | 2017 | Andaman &Nicobar | 786 | 0.51 | 0.0178 | female | 8 |  |
| Meshram II et al., 2020(a) | 2020 | - | Nagaland | 53 | 0.52 | 0.0686 | female | 7 |  |
| Nair M et al., 2016 | 2016 | 2015 | Assam | 1007 | 0.35 | 0.0150 | female | 9 |  |
| Patel A et al., 2018 | 2018 | 2016 | Maharashtra | 72750 | 0.90 | 0.0011 | female | 7 |  |
| Rajaratnam J et al., 2000 | 2000 | 1996 | Tamil Nadu | 895 | 0.36 | 0.0161 | female | 4 |  |
| Samuel TM et al., 2013 | 2013 | - | Karnataka | 366 | 0.30 | 0.0240 | female | 7 |  |
| Sharma JB et al., 2003 | 2003 | 2000 | Delhi | 1150 | 0.96 | 0.0058 | female | 5 |  |
| Siddiqui M.Z et al., 2017 | 2017 | - | India | 5911 | 0.59 | 0.0064 | female | 7 |  |
| Siddiqui R et al., 2014 | 2014 | 2011 | Telangana | 250 | 0.93 | 0.0159 | female | 5 |  |
| Singh P et al., 2015 | 2015 | 2014 | Uttar Pradesh | 300 | 0.58 | 0.0285 | female | 8 |  |
| Sinha A et al., 2021 | 2021 | - | West Bengal | 200 | 0.90 | 0.0212 | female | 7 |  |
| Suryanarayana R et al., 2017 | 2017 | 2015 | Karnataka | 446 | 0.62 | 0.0229 | female | 7 |  |
| Toteja GS et al., 2006 | 2006 | - | India | 6923 | 0.85 | 0.0043 | female | 5 |  |
| Vemulapalli B et al., 2014 | 2014 | 2011 | Andhra Pradesh | 986 | 1.00 | 0.0005 | female | 6 |  |
| Vindhya J et al., 2019 | 2019 | 2018 | Karnataka | 280 | 0.34 | 0.0283 | female | 7 |  |
| Arora K et al., 2022 | 2022 | 2018 | Uttarakhand | 151 | 0.37 | 0.0393 | female | 6 | Antenatal women |
| Nair MS et al., 2022 | 2022 | 2017 | Kerala | 295 | 0.40 | 0.0285 |  | 9 |  |
| Noronha JA et al., 2010 | 2010 | - | Karnataka | 1077 | 0.50 | 0.0152 | female | 8 |  |
| Saraswathi K.S et al., 2013 | 2013 | 2012 | India | 9642 | 0.51 | 0.0051 |  | 4 |  |
| Yadav U et al., 2020 | 2020 | - | Bihar | 631 | 0.92 | 0.0108 | female | 5 |  |
| Agarwal KN et al., 2006 | 2006 | 2003 | Assam | 93 | 0.96 | 0.0210 |  | 6 | Lactating women |
|  | 2006 | 2003 | Haryana | 112 | 0.96 | 0.0196 | female | 6 |  |
|  | 2006 | 2003 | Himachal Pradesh | 76 | 0.91 | 0.0332 |  | 6 |  |
|  | 2006 | 2003 | Kerala | 56 | 0.61 | 0.0653 | female | 6 |  |
|  | 2006 | 2003 | Madhya Pradesh | 44 | 1.00 | 0.0048 |  | 6 |  |
|  | 2006 | 2003 | Orissa | 134 | 0.99 | 0.0072 | female | 6 |  |
|  | 2006 | 2003 | Tamil Nadu | 88 | 0.91 | 0.0307 |  | 6 |  |
| Arlappa N. et al., 2014(c) | 2014 | - | Maharashtra | 410 | 0.73 | 0.0220 | female | 9 |  |
| Bhatia V. et al., 2020 | 2020 | - | Orissa | 788 | 0.77 | 0.0150 |  | 8 |  |
| Meshram II et al., 2020(a) | 2020 | - | Nagaland | 198 | 0.44 | 0.0353 | female | 7 |  |
| Shimrah C et al., 2022 | 2022 | - | Manipur | 150 | 0.62 | 0.0396 |  | 6 |  |
| Siddiqui M.Z et al., 2017 | 2017 | - | India | 21973 | 0.63 | 0.0033 | female | 7 |  |
| Bhagwan D et al., 2016 | 2016 | - | Karnataka | 343 | 0.27 | 0.0238 |  | 9 | Postnatal women |
| Noronha JA et al., 2010 | 2010 | - | Karnataka | 1000 | 0.54 | 0.0158 | female | 8 |  |
| Rakesh P et al., 2014 | 2014 | - | Tamil Nadu | 93 | 0.47 | 0.0518 |  | 8 |  |
| Selvaraj R et al., 2019 | 2019 | 2016 | Pondicherry | 227 | 0.76 | 0.0283 | female | 8 |  |
| Kant S. et al., 2018 | 2018 | 2016 | Haryana | 8748 | 0.78 | 0.0044 |  | 5 | Delivering women |
| Kumari S et al., 2019 | 2019 | 2017 | Jharkhand | 515 | 0.78 | 0.0181 | female | 8 |  |
| Manjula VD et al., 2014 | 2014 | 2012 | Kerala | 183 | 0.19 | 0.0291 | female | 9 | Medical  students |
| Rani NA et al., 2017 | 2017 | 2015 | Karnataka | 289 | 0.16 | 0.0213 | both | 6 |  |
| Vibhute NA et al., 2019 | 2019 | - | Maharashtra | 300 | 0.29 | 0.0261 | both | 5 |  |

**Supplementary Table 2: Quality assessment of included studies using Newcastle-Ottawa scale**

| **Study ID** | **Selection** | | | | **Comparability** | **Outcome** | **Statistics** | **Score** |
| --- | --- | --- | --- | --- | --- | --- | --- | --- |
|  | **Sampling mentioned** | **Sample size justified** | **Non respondent** | **Ascertainment of exposure (max**)-genuine source** | **Confounding controlled (max**)** | **Outcome assessment (max**)- genuine tool** | **Statistical test justified** | **Total** |
| Agarwal KN et al., 2006 | 1 | 0 | 0 | 2 | 0 | 2 | 1 | 6 |
| Agarwalla R. et al., 2016 | 0 | 1 | 0 | 2 | 2 | 2 | 1 | 8 |
| Ahankari AS et.al 2017 | 0 | 0 | 1 | 2 | 2 | 2 | 1 | 8 |
| Ahmad N. et al., 2010 | 1 | 1 | 0 | 2 | 0 | 2 | 1 | 7 |
| Arlappa N. et al., 2010(a) | 1 | 1 | 0 | 2 | 2 | 2 | 1 | 9 |
| Arlappa N. et al., 2012(b) | 1 | 1 | 0 | 2 | 2 | 2 | 1 | 9 |
| Arlappa N. et al., 2014(c) | 1 | 1 | 0 | 2 | 2 | 2 | 1 | 9 |
| Arora K et al., 2022 | 1 | 1 | 0 | 2 | 0 | 2 | 0 | 6 |
| Awasthi S et al., 2003 | 0 | 1 | 0 | 2 | 0 | 2 | 1 | 6 |
| Bala DV et al., 2012 | 1 | 1 | 0 | 2 | 0 | 2 | 1 | 7 |
| Banerjee M et al., 2022 | 0 | 0 | 0 | 2 | 0 | 2 | 1 | 5 |
| Basu S et al., 2005 | 0 | 1 | 1 | 2 | 0 | 2 | 1 | 7 |
| Behera S et al., 2016 | 0 | 0 | 0 | 2 | 0 | 2 | 1 | 5 |
| Bentley M.E. et al., 2003 | 1 | 0 | 0 | 2 | 2 | 2 | 1 | 8 |
| Bhagwan D et al., 2016 | 1 | 1 | 0 | 2 | 2 | 2 | 1 | 9 |
| Bharati P. et al., 2008(a) | 1 | 0 | 0 | 2 | 2 | 2 | 1 | 8 |
| Bharati P. et al.,2009(b) | 0 | 0 | 0 | 2 | 0 | 2 | 1 | 5 |
| Bhardwaj A et al., 2013 | 1 | 0 | 0 | 2 | 0 | 2 | 1 | 6 |
| Bhatia V. et al., 2020 | 1 | 1 | 1 | 2 | 0 | 2 | 1 | 8 |
| Bhattacharyya K et al., 2010 | 1 | 0 | 0 | 2 | 0 | 2 | 0 | 5 |
| Biradar S.S. et al., 2012 | 1 | 1 | 0 | 2 | 0 | 2 | 1 | 7 |
| Bone JN et al., 2022 | 0 | 0 | 0 | 2 | 2 | 2 | 1 | 7 |
| Bora R et al., 2014 | 0 | 0 | 0 | 2 | 0 | 2 | 1 | 5 |
| Bulliyy G et al., 2007 | 1 | 1 | 0 | 2 | 2 | 2 | 1 | 9 |
| Chandrakumari AS et al., 2019 | 0 | 1 | 0 | 2 | 2 | 2 | 0 | 7 |
| Chaudhary SM et al., 2008 | 1 | 1 | 0 | 2 | 2 | 2 | 1 | 9 |
| Chauhan S et al., 2022 | 0 | 0 | 1 | 2 | 0 | 2 | 1 | 6 |
| Chowdhury T.K. et al., 2019 | 0 | 0 | 0 | 2 | 2 | 2 | 1 | 7 |
| Corrêa G et al., 2017 | 0 | 0 | 0 | 2 | 2 | 2 | 1 | 7 |
| Debnath A et al., 2021(a) | 0 | 1 | 0 | 2 | 0 | 2 | 1 | 6 |
| Debnath A et al., 2022(b) | 1 | 1 | 1 | 2 | 2 | 2 | 1 | 10 |
| Dey S. et al., 2010 | 0 | 0 | 0 | 2 | 2 | 2 | 1 | 7 |
| Didzun O et al., 2019 | 1 | 0 | 1 | 2 | 2 | 2 | 1 | 9 |
| Dudeja P et al., 2016 | 1 | 1 | 0 | 2 | 0 | 2 | 0 | 6 |
| Finkelstein JL et al., 2020(a) | 1 | 0 | 0 | 2 | 2 | 2 | 1 | 8 |
| Finkelstein JL et al., 2021(b) | 1 | 0 | 0 | 2 | 0 | 2 | 1 | 6 |
| George KA et al., 2000 | 0 | 0 | 0 | 2 | 2 | 2 | 1 | 7 |
| Ghosh P et al., 2020 | 0 | 1 | 0 | 2 | 2 | 2 | 1 | 8 |
| Gogoi I et al., 2016 | 1 | 1 | 0 | 2 | 2 | 2 | 1 | 9 |
| Gonmei Z et al., 2018 | 0 | 0 | 0 | 2 | 0 | 2 | 0 | 4 |
| Gopalakrishnan S et al., 2018 | 1 | 0 | 0 | 2 | 2 | 2 | 1 | 8 |
| Gosdin L et al., 2018 | 1 | 0 | 0 | 2 | 2 | 2 | 1 | 8 |
| Goswmai S et al 2015 | 1 | 0 | 0 | 2 | 2 | 2 | 1 | 8 |
| Goyle A Jr et al., 2009 | 0 | 0 | 0 | 2 | 0 | 2 | 0 | 4 |
| Grover K et al., 2020 | 1 | 1 | 0 | 2 | 2 | 2 | 0 | 8 |
| Gunjal Sandeep, S. et al., 2012 | 1 | 1 | 1 | 2 | 0 | 2 | 1 | 8 |
| Gupta S et al., 2012(a) | 1 | 1 | 0 | 2 | 0 | 2 | 0 | 6 |
| Gupta S et al., 2017(b) | 0 | 1 | 0 | 2 | 0 | 2 | 0 | 5 |
| Gupta S et al., 2021(c) | 1 | 1 | 0 | 2 | 2 | 2 | 1 | 9 |
| Gupta V.K. et al., 2011 | 0 | 0 | 0 | 2 | 0 | 2 | 0 | 4 |
| Haralkar S.J et al., 2013 | 0 | 0 | 1 | 2 | 0 | 2 | 0 | 5 |
| Jagadish Kumar K et al 2017 | 0 | 1 | 0 | 2 | 0 | 2 | 1 | 6 |
| Jain T et al., 2011 | 0 | 1 | 0 | 2 | 2 | 2 | 1 | 8 |
| Jana A et al., 2022 | 1 | 0 | 0 | 2 | 2 | 2 | 1 | 8 |
| Jones AD et. al., 2016 | 0 | 0 | 0 | 2 | 0 | 2 | 1 | 5 |
| Kalhan M et al., 2022 | 1 | 1 | 0 | 2 | 2 | 2 | 0 | 8 |
| Kamath R et al., 2013 | 0 | 1 | 0 | 2 | 0 | 2 | 0 | 5 |
| Kamble BD et al., 2021 | 0 | 0 | 0 | 2 | 2 | 2 | 1 | 7 |
| Kandasamy K. et al., 2017 | 0 | 1 | 0 | 2 | 0 | 2 | 0 | 5 |
| Kant S et al., 2018(a) | 0 | 0 | 0 | 2 | 0 | 2 | 1 | 5 |
| Kant S et al., 2019(b) | 1 | 1 | 1 | 2 | 2 | 2 | 1 | 10 |
| Kapoor D et al., 2002 | 0 | 1 | 0 | 2 | 0 | 2 | 1 | 6 |
| Kaur M 2018 | 1 | 0 | 0 | 2 | 0 | 2 | 1 | 6 |
| Kishore S et al., 2020 | 0 | 0 | 0 | 2 | 0 | 2 | 1 | 5 |
| Krishnaswamy S et al., 2017 | 0 | 0 | 0 | 2 | 0 | 2 | 0 | 4 |
| Krupp K et al., 2018 | 0 | 0 | 0 | 2 | 2 | 2 | 1 | 7 |
| Kumar et al., 2023 | 0 | 0 | 0 | 2 | 0 | 2 | 0 | 4 |
| Kumar P et al., 2021(a) | 1 | 0 | 0 | 2 | 0 | 2 | 0 | 5 |
| Kumar P et al., 2021(b) | 1 | 0 | 0 | 2 | 2 | 2 | 1 | 8 |
| Kumar T et al., 2014 | 0 | 0 | 1 | 2 | 2 | 2 | 1 | 8 |
| Kumar V.et al., 2015 | 1 | 0 | 1 | 2 | 2 | 2 | 1 | 9 |
| Kumari R et al., 2017 | 0 | 0 | 0 | 2 | 0 | 2 | 0 | 4 |
| Kumari S et al., 2019 | 1 | 0 | 0 | 2 | 2 | 2 | 1 | 8 |
| Little M et al., 2018 | 1 | 0 | 0 | 2 | 2 | 2 | 1 | 8 |
| Mahanta T.G. et al 2015 | 1 | 1 | 0 | 2 | 2 | 2 | 1 | 9 |
| Mahashabde P et al., 2014 | 0 | 1 | 0 | 2 | 2 | 2 | 1 | 8 |
| Malhotra P et al., 2004 | 0 | 0 | 0 | 2 | 0 | 2 | 0 | 4 |
| Mandal et al., 2022 | 0 | 0 | 0 | 2 | 2 | 2 | 1 | 7 |
| Mangla M et al., 2016 | 0 | 0 | 0 | 2 | 0 | 2 | 1 | 5 |
| Manjula AA et al., 2003 | 0 | 0 | 0 | 2 | 0 | 2 | 0 | 4 |
| Manjula VD et al., 2014 | 1 | 1 | 0 | 2 | 2 | 2 | 1 | 9 |
| Mehrotra M et al., 2018 | 0 | 0 | 1 | 2 | 2 | 2 | 1 | 8 |
| Meshram II et al., 2020(a) | 0 | 0 | 0 | 2 | 2 | 2 | 1 | 7 |
| Meshram II et.al 2021(b) | 1 | 1 | 0 | 2 | 0 | 2 | 1 | 7 |
| Mishra N. et al., 2016 | 0 | 0 | 0 | 2 | 2 | 2 | 1 | 7 |
| Muthayya S et al., 2007 | 0 | 0 | 0 | 2 | 0 | 2 | 1 | 5 |
| Nadar, S. et al., 2016 | 0 | 0 | 0 | 2 | 0 | 2 | 0 | 4 |
| Nair et al., 2023 | 1 | 1 | 0 | 2 | 2 | 2 | 1 | 9 |
| Nair KM et al., 2016 | 0 | 0 | 0 | 2 | 2 | 2 | 1 | 7 |
| Nair M et al., 2015 | 1 | 1 | 0 | 2 | 2 | 2 | 1 | 9 |
| Nair MS et al., 2022 | 1 | 1 | 0 | 2 | 2 | 2 | 1 | 9 |
| Noronha JA et al., 2010 | 1 | 0 | 0 | 2 | 2 | 2 | 1 | 8 |
| Onyeneho NG et al., 2019 | 1 | 0 | 0 | 2 | 2 | 2 | 1 | 8 |
| Osborn AJ et al., 2021 | 1 | 1 | 0 | 2 | 2 | 2 | 1 | 9 |
| Panchal SS et al., 2022 | 1 | 0 | 0 | 2 | 2 | 2 | 1 | 8 |
| Panyang R et al., 2018 | 0 | 1 | 0 | 2 | 0 | 2 | 1 | 6 |
| Pasricha SR et al., 2010 | 0 | 1 | 0 | 2 | 2 | 2 | 1 | 8 |
| Patel A et al., 2018 | 0 | 0 | 0 | 2 | 2 | 2 | 1 | 7 |
| Pathania A et al., 2019 | 1 | 1 | 0 | 2 | 2 | 2 | 1 | 9 |
| Philip RR et al., 2015 | 1 | 1 | 0 | 2 | 0 | 2 | 1 | 7 |
| Prabhakar SCJ et al., 2009 | 0 | 1 | 0 | 2 | 0 | 2 | 0 | 5 |
| Rahman MHU et al., 2020 | 1 | 0 | 1 | 2 | 2 | 2 | 1 | 9 |
| Rai RK et al., 2023 | 1 | 0 | 1 | 2 | 0 | 2 | 1 | 7 |
| Rajaratnam J et al., 2000 | 0 | 0 | 0 | 2 | 0 | 2 | 0 | 4 |
| Rakesh P et al., 2014 | 0 | 1 | 0 | 2 | 2 | 2 | 1 | 8 |
| Rakesh PS et al., 2019 | 1 | 1 | 1 | 2 | 2 | 2 | 1 | 10 |
| Rakesh SR et al., 2014 | 0 | 0 | 0 | 2 | 2 | 2 | 0 | 6 |
| Ramesh Masthi NR et.al 2012 | 1 | 0 | 0 | 2 | 0 | 2 | 0 | 5 |
| Rani NA et al., 2017 | 1 | 0 | 0 | 2 | 0 | 2 | 1 | 6 |
| Rao S et al., 2011 | 0 | 0 | 0 | 2 | 0 | 2 | 1 | 5 |
| Retnakumar C et al., 2020 | 1 | 1 | 0 | 2 | 2 | 2 | 1 | 9 |
| Rohisha IK et al., 2019 | 1 | 1 | 1 | 2 | 0 | 2 | 0 | 7 |
| S RP et al., 2015 | 1 | 1 | 0 | 2 | 2 | 2 | 1 | 9 |
| Saba F et al., 2014 | 1 | 0 | 0 | 2 | 0 | 2 | 1 | 6 |
| Sahoo J et al., 2021 | 1 | 0 | 1 | 2 | 0 | 2 | 1 | 7 |
| Samuel TM et al., 2013 | 0 | 0 | 0 | 2 | 2 | 2 | 1 | 7 |
| Saraswathi K.S et al.,2013 | 0 | 0 | 0 | 2 | 0 | 2 | 0 | 4 |
| Sarna A et al., 2020 | 1 | 0 | 0 | 2 | 0 | 2 | 1 | 6 |
| Toteja GS et al., 2006 | 1 | 0 | 0 | 2 | 0 | 2 | 0 | 5 |
| Selvaraj R et al., 2019 | 1 | 0 | 0 | 2 | 2 | 2 | 1 | 8 |
| Sen A et al., 2006 | 1 | 0 | 0 | 2 | 0 | 2 | 0 | 5 |
| Seth R.K et al., 2015 | 1 | 1 | 0 | 2 | 2 | 2 | 0 | 8 |
| Shanmugam J et al., 2023 | 0 | 1 | 0 | 2 | 2 | 2 | 1 | 8 |
| Sharma JB et al., 2003 | 0 | 0 | 0 | 2 | 0 | 2 | 1 | 5 |
| Sharma SK et al., 2012 | 1 | 1 | 0 | 2 | 0 | 2 | 1 | 7 |
| Sharma U et al., 2019 | 1 | 1 | 0 | 2 | 2 | 2 | 1 | 9 |
| Shimrah C et al., 2022 | 0 | 1 | 0 | 2 | 0 | 2 | 1 | 6 |
| Shrinivasa BM et al., 2014 | 0 | 1 | 0 | 2 | 2 | 2 | 0 | 7 |
| Siddiqui M.Z et al., 2017 | 0 | 0 | 0 | 2 | 2 | 2 | 1 | 7 |
| Siddiqui R. et al., 2014 | 0 | 0 | 0 | 2 | 0 | 2 | 1 | 5 |
| Simhachalam Naidu C.H et al., 2014 | 0 | 0 | 0 | 2 | 2 | 2 | 0 | 6 |
| Singh A et al., 2022 | 1 | 0 | 1 | 2 | 2 | 2 | 1 | 9 |
| Singh B et al., 2022 | 0 | 0 | 0 | 2 | 0 | 2 | 0 | 4 |
| Singh G. et.al 2017 | 1 | 0 | 0 | 2 | 0 | 2 | 1 | 6 |
| Singh P et al., 2015 | 0 | 1 | 0 | 2 | 2 | 2 | 1 | 8 |
| Singh R.K. 2013 (a) | 0 | 0 | 0 | 2 | 2 | 2 | 1 | 7 |
| Singh RK et al., 2014(b) | 0 | 0 | 0 | 2 | 0 | 2 | 1 | 5 |
| Singh T et al., 2018 | 1 | 1 | 0 | 2 | 2 | 2 | 1 | 9 |
| Sinha A et al., 2021 | 0 | 0 | 0 | 2 | 2 | 2 | 1 | 7 |
| Sinha N.K. et al., 2013 | 0 | 0 | 0 | 2 | 2 | 2 | 1 | 7 |
| Siva PM et al., 2016 | 1 | 1 | 0 | 2 | 2 | 2 | 1 | 9 |
| Srivastava S et al., 2022 | 1 | 0 | 0 | 2 | 0 | 2 | 1 | 6 |
| Stiller CK et al., 2020 | 1 | 1 | 1 | 2 | 0 | 2 | 1 | 8 |
| Subramanian M et al., 2022 | 1 | 1 | 0 | 2 | 2 | 2 | 1 | 9 |
| Sudhagandhi B. et al., 2012 | 0 | 0 | 0 | 2 | 0 | 2 | 1 | 5 |
| Sulakshana B. et al., 2014 | 0 | 0 | 0 | 2 | 2 | 2 | 1 | 7 |
| Suryanarayana R et al., 2017 | 1 | 1 | 0 | 2 | 0 | 2 | 1 | 7 |
| Thankachan P et al., 2007 | 0 | 0 | 0 | 2 | 0 | 2 | 1 | 5 |
| Vemulapalli B et al., 2014 | 0 | 0 | 0 | 2 | 2 | 2 | 0 | 6 |
| Verma K et al., 2022 | 0 | 1 | 0 | 2 | 2 | 2 | 1 | 8 |
| Verma M et al., 1998 | 0 | 0 | 0 | 2 | 0 | 2 | 1 | 5 |
| Verma R. et al., 2015 | 0 | 0 | 0 | 2 | 2 | 2 | 1 | 7 |
| Vibhute NA et al., 2019 | 1 | 0 | 0 | 2 | 0 | 2 | 0 | 5 |
| Vindhya J et al., 2019 | 0 | 0 | 0 | 2 | 2 | 2 | 1 | 7 |
| Wangaskar SA et al., 2021 | 1 | 1 | 0 | 2 | 2 | 2 | 1 | 9 |
| William R.F et al., 2016 | 0 | 1 | 0 | 2 | 2 | 2 | 1 | 8 |
| Yadav U et al., 2020 | 0 | 0 | 0 | 2 | 0 | 2 | 1 | 5 |

Studies having total score >5 are considered as of moderate quality and otherwise weak quality.

### Supplementary Figure 1: Sensitivity analysis of pooled prevalence of anemia- among toddlers (under 3 years)

### Supplementary Figure 2: Cumulative meta-analysis of pooled prevalence of anemia- among toddlers (under 3 years)


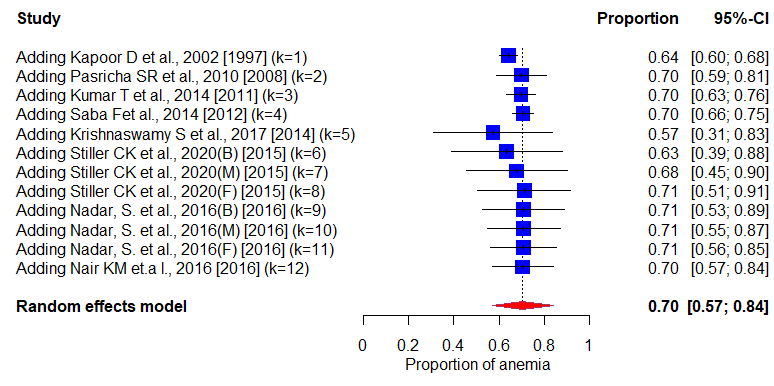


### Supplementary Figure 3: Subgroup analysis of pooled prevalence of anemia- among toddlers (under 3 years) based on region

### Supplementary Figure 4: Subgroup analysis of pooled prevalence of anemia- among toddlers (under 3 years) based on states & union territories

### Supplementary Figure 5: Subgroup analysis of pooled prevalence of anemia- among toddlers (under 3 years) based on gender

### Supplementary Figure 6: Cumulative meta-analysis of pooled prevalence of anemia- among pre-school children (3-5 years)

###
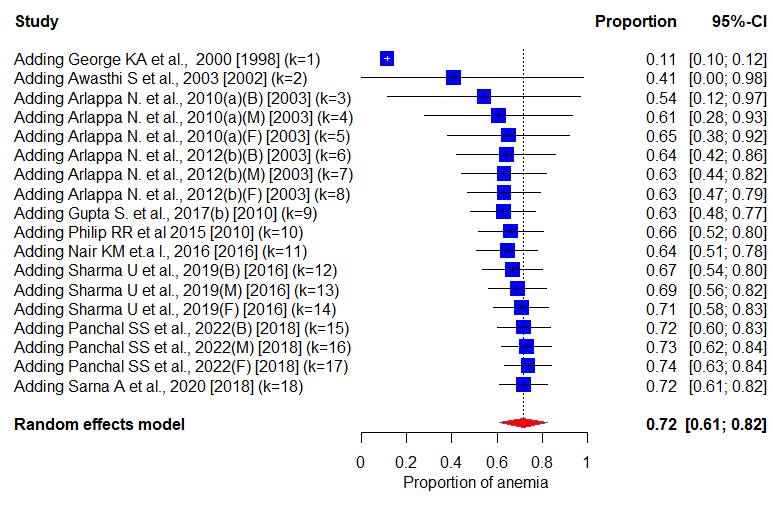


### Supplementary Figure 7: Subgroup analysis of pooled prevalence of anemia- among pre-school children (3-5 years) based on region

### Supplementary Figure 8: Subgroup analysis of pooled prevalence of anemia- among pre-school children (3-5 years) based on states & union territories

### Supplementary Figure 9: Subgroup analysis of pooled prevalence of anemia- among pre-school children (3-5 years) based on gender

### Supplementary Table 3: Meta-analysis of pooled prevalence of anemia- among school children (6-18 years)

| **Study** | **Effect Size** | **95 % CI** | **% Weight** |
| --- | --- | --- | --- |
| Ahankari AS et.al., 2017 | 0.870 | [0.849; 0.891] | 1.17 |
| Arlappa N et al., 2014(c) | 0.611 | [0.578; 0.644] | 1.17 |
| Banerjee M et al., 2022(B) | 0.344 | [0.321; 0.367] | 1.17 |
| Banerjee M et al., 2022(M) | 0.297 | [0.269; 0.325] | 1.17 |
| Banerjee M et al., 2022(F) | 0.444 | [0.406; 0.482] | 1.16 |
| Basu S et al., 2005(B) | 0.163 | [0.141; 0.184] | 1.17 |
| Basu S et al., 2005(M) | 0.077 | [0.054; 0.100] | 1.17 |
| Basu S et al., 2005(F) | 0.239 | [0.205; 0.273] | 1.17 |
| Behera S et al., 2016(B) | 0.689 | [0.627; 0.751] | 1.15 |
| Behera S et al., 2016(M) | 0.729 | [0.635; 0.823] | 1.13 |
| Behera S et al., 2016(F) | 0.661 | [0.579; 0.743] | 1.14 |
| Bharati P et al., 2009(b) | 0.900 | [0.899; 0.901] | 1.17 |
| Bhatia V et al., 2020 | 0.68 | [0.648; 0.712] | 1.17 |
| Biradar S.S et al., 2012 | 0.411 | [0.378; 0.444] | 1.17 |
| Bulliyy G et al., 2007 | 0.965 | [0.957; 0.973] | 1.17 |
| Chandrakumari AS et al., 2019 | 0.486 | [0.421; 0.552] | 1.15 |
| Chaudhary SM et al., 2008 | 0.351 | [0.297; 0.405] | 1.16 |
| Chauhan S et al., 2022 (F-Bihar) | 0.200 | [0.189; 0.211] | 1.17 |
| Chauhan S et al., 2022 (M-Bihar) | 0.087 | [0.074; 0.100] | 1.17 |
| Chauhan S et al., 2022 (F-Uttar Pradesh) | 0.200 | [0.192; 0.208] | 1.17 |
| Chauhan S et al., 2022 (M-Uttar Pradesh) | 0.087 | [0.078; 0.096] | 1.17 |
| Gopalakrishnan S et al., 2018 | 0.848 | [0.803; 0.893] | 1.16 |
| Goyle A Jr et al., 2009 | 0.963 | [0.928; 0.998] | 1.17 |
| Gunjal S et al., 2012 (B) | 0.897 | [0.878; 0.917] | 1.17 |
| Gunjal S et al., 2012 (M) | 0.877 | [0.847; 0.907] | 1.17 |
| Gunjal S et al., 2012 (F) | 0.919 | [0.894; 0.944] | 1.17 |
| Gupta S et al., 2012 (a) | 0.802 | [0.742; 0.862] | 1.15 |
| Gupta S. et al., 2017 (b) | 0.497 | [0.43; 0.564] | 1.15 |
| Jagadish Kumar K et al., 2017 (B) | 0.525 | [0.456; 0.594] | 1.15 |
| Jagadish Kumar K et al., 2017 (M) | 0.525 | [0.447; 0.603] | 1.14 |
| Jagadish Kumar K et al., 2017 (F) | 0.522 | [0.378; 0.666] | 1.07 |
| Jain T et al., 2011 | 0.428 | [0.38; 0.476] | 1.16 |
| Kamble BD et al., 2021 | 0.59 | [0.522; 0.658] | 1.15 |
| Kumar et al., 2023 | 0.279 | [0.234; 0.323] | 1.16 |
| Kumari R et al., 2017 | 0.500 | [0.431; 0.569] | 1.15 |
| Mahanta T.G. et al 2015 | 0.963 | [0.95; 0.976] | 1.17 |
| Manjula AA et al., 2003 | 0.442 | [0.409; 0.475] | 1.17 |
| Muthayya S et al., 2007 | 0.136 | [0.121; 0.151] | 1.17 |
| Nair et al., 2023 | 0.657 | [0.612; 0.702] | 1.16 |
| Prabhakar SCJ et al., 2009 | 0.777 | [0.715; 0.839] | 1.15 |
| Rahman MHU et al., 2020 | 0.234 | [0.227; 0.241] | 1.17 |
| Rai RK et al., 2023(F-Bihar-2016) | 0.598 | [0.562; 0.634] | 1.17 |
| Rai RK et al., 2023(F-Bihar-2019) | 0.738 | [0.705; 0.771] | 1.17 |
| Rai RK et al., 2023(M-Bihar-2016) | 0.286 | [0.257; 0.315] | 1.17 |
| Rai RK et al., 2023(M-Bihar-2019) | 0.307 | [0.277; 0.337] | 1.17 |
| Rai RK et al., 2023(F-Uttar Pradesh-2016) | 0.565 | [0.531; 0.599] | 1.17 |
| Rai RK et al., 2023(F-Uttar Pradesh-2019) | 0.586 | [0.552; 0.620] | 1.17 |
| Rai RK et al., 2023(M-Uttar Pradesh-2016) | 0.369 | [0.337; 0.401] | 1.17 |
| Rai RK et al., 2023(M-Uttar Pradesh-2019) | 0.321 | [0.290; 0.352] | 1.17 |
| Rakesh PS et al., 2019(B) | 0.440 | [0.407; 0.473] | 1.17 |
| Rakesh PS et al., 2019(M) | 0.396 | [0.350; 0.442] | 1.16 |
| Rakesh PS et al., 2019(F) | 0.477 | [0.430; 0.524] | 1.16 |
| Rakesh SR et al., 2014 | 0.723 | [0.710; 0.736] | 1.17 |
| Ramesh Masthi NR et.al., 2012(B) | 0.087 | [0.056; 0.118] | 1.17 |
| Ramesh Masthi NR et.al., 2012(M) | 0.075 | [0.032; 0.118] | 1.16 |
| Ramesh Masthi NR et.al., 2012(F) | 0.097 | [0.053; 0.141] | 1.16 |
| S RP et al., 2015(B) | 0.314 | [0.298; 0.330] | 1.17 |
| S RP et al., 2015(M) | 0.318 | [0.295; 0.341] | 1.17 |
| S RP et al., 2015(F) | 0.311 | [0.288; 0.334] | 1.17 |
| Sahoo J et al., 2021 | 0.455 | [0.424; 0.487] | 1.17 |
| Sarna A et al., 2020 | 0.284 | [0.277; 0.291] | 1.17 |
| Sen A et al., 2006 | 0.670 | [0.619; 0.721] | 1.16 |
| Shanmugam J et al., 2023(B) | 0.886 | [0.873; 0.899] | 1.17 |
| Shanmugam J et al., 2023(F) | 0.502 | [0.472; 0.532] | 1.17 |
| Shanmugam J et al., 2023(M) | 0.498 | [0.470; 0.526] | 1.17 |
| Sharma SK et al., 2012 | 0.715 | [0.702; 0.728] | 1.17 |
| Simhachalam Naidu C.H et al., 2014 | 0.793 | [0.747; 0.839] | 1.16 |
| Siva PM et al., 2016 | 0.210 | [0.160; 0.260] | 1.16 |
| Srivastava S et al., 2022(F-Bihar2016) | 0.588 | [0.569; 0.607] | 1.17 |
| Srivastava S et al., 2022(F-Bihar-2019) | 0.628 | [0.609; 0.647] | 1.17 |
| Srivastava S et al., 2022(M-Bihar-2016) | 0.327 | [0.303; 0.351] | 1.17 |
| Srivastava S et al., 2022(M-Bihar-2019) | 0.305 | [0.281; 0.329] | 1.17 |
| Srivastava S et al., 2022(F-Uttar Pradesh-2016) | 0.588 | [0.571; 0.605] | 1.17 |
| Srivastava S et al., 2022(F-Uttar Pradesh-2019) | 0.628 | [0.612; 0.644] | 1.17 |
| Srivastava S et al., 2022(M-Uttar Pradesh-2016) | 0.327 | [0.309; 0.345] | 1.17 |
| Srivastava S et al., 2022(M-Uttar Pradesh-2019) | 0.305 | [0.287; 0.323] | 1.17 |
| Subramanian M et al., 2022 | 0.717 | [0.663; 0.771] | 1.16 |
| Sulakshana B et al., 2014 | 0.750 | [0.708; 0.792] | 1.16 |
| Toteja GS et al., 2006 | 0.901 | [0.892; 0.910] | 1.17 |
| Verma K et al., 2022 | 0.563 | [0.524; 0.602] | 1.16 |
| Verma M et al., 1998 | 0.515 | [0.493; 0.537] | 1.17 |
| Wangaskar SA et al., 2021(B) | 0.627 | [0.583; 0.671] | 1.16 |
| Wangaskar SA et al., 2021(F) | 0.618 | [0.567; 0.669] | 1.16 |
| Wangaskar SA et al., 2021(M) | 0.652 | [0.564; 0.74] | 1.13 |
| William R.F. et al., 2016 | 0.613 | [0.546; 0.680] | 1.15 |
| **Pooled estimate** | **0.512** | **[0.460; 0.564]** |  |
| Test of Pooled estimate = 0: z = 19.30 Prob > |z| = 0.0000  Test of homogeneity: Q = chi2(84) = 1.9e+05 Prob > Q = 0.0000 | | | |

### Supplementary Table 4: Sensitivity analysis of pooled prevalence of anemia- among school children (6-18 years)

| **Study** | **Effect Size** | **95 % CI** | **% Weight** |
| --- | --- | --- | --- |
| Ahankari AS et.al., 2017 | 0.870 | [0.849; 0.891] | 1.24 |
| Arlappa N et al., 2014(c) | 0.611 | [0.578; 0.644] | 1.24 |
| Banerjee M et al., 2022(B) | 0.344 | [0.321; 0.367] | 1.24 |
| Banerjee M et al., 2022(M) | 0.297 | [0.269; 0.325] | 1.24 |
| Banerjee M et al., 2022(F) | 0.444 | [0.406; 0.482] | 1.24 |
| Basu S et al., 2005(B) | 0.163 | [0.141; 0.184] | 1.24 |
| Basu S et al., 2005(M) | 0.077 | [0.054; 0.100] | 1.24 |
| Basu S et al., 2005(F) | 0.239 | [0.205; 0.273] | 1.24 |
| Behera S et al., 2016(B) | 0.689 | [0.627; 0.751] | 1.22 |
| Behera S et al., 2016(M) | 0.729 | [0.635; 0.823] | 1.20 |
| Behera S et al., 2016(F) | 0.661 | [0.579; 0.743] | 1.21 |
| Bharati P et al., 2009(b) | 0.900 | [0.899; 0.901] | 1.24 |
| Bhatia V et al., 2020 | 0.680 | [0.648; 0.712] | 1.24 |
| Biradar S.S et al., 2012 | 0.411 | [0.378; 0.444] | 1.24 |
| Bulliyy G et al., 2007 | 0.965 | [0.957; 0.973] | 1.24 |
| Chandrakumari AS et al., 2019 | 0.486 | [0.421; 0.552] | 1.22 |
| Chaudhary SM et al., 2008 | 0.351 | [0.297; 0.405] | 1.23 |
| Chauhan S et al., 2022(F-Bihar) | 0.200 | [0.189; 0.211] | 1.24 |
| Chauhan S et al., 2022(M-Bihar) | 0.087 | [0.074; 0.100] | 1.24 |
| Chauhan S et al., 2022(F-Uttar Pradesh) | 0.200 | [0.192; 0.208] | 1.24 |
| Chauhan S et al., 2022(M-Uttar Pradesh) | 0.087 | [0.078; 0.096] | 1.24 |
| Gopalakrishnan S et al., 2018 | 0.848 | [0.803; 0.893] | 1.23 |
| Gunjal Sandeep, S et al., 2012(B) | 0.897 | [0.878; 0.917] | 1.24 |
| Gunjal Sandeep, S et al., 2012(M) | 0.877 | [0.847; 0.907] | 1.24 |
| Gunjal Sandeep, S et al., 2012(F) | 0.919 | [0.894; 0.944] | 1.24 |
| Gupta S et al., 2012(a) | 0.802 | [0.742; 0.862] | 1.23 |
| Gupta S et al., 2017(b) | 0.497 | [0.430; 0.564] | 1.22 |
| Jagadish Kumar K et al., 2017(B) | 0.525 | [0.456; 0.594] | 1.22 |
| Jagadish Kumar K et al., 2017(M) | 0.525 | [0.447; 0.603] | 1.21 |
| Jagadish Kumar K et al., 2017(F) | 0.522 | [0.378; 0.666] | 1.14 |
| Jain T. et al., 2011 | 0.428 | [0.380; 0.476] | 1.23 |
| Kamble BD et al., 2021 | 0.590 | [0.522; 0.658] | 1.22 |
| Mahanta T.G et al., 2015 | 0.963 | [0.950; 0.976] | 1.24 |
| Muthayya S et al., 2007 | 0.136 | [0.121; 0.151] | 1.24 |
| Nair et al., 2023 | 0.657 | [0.612; 0.702] | 1.23 |
| Prabhakar SCJ et al., 2009 | 0.777 | [0.715; 0.839] | 1.22 |
| Rahman MHU et al., 2020 | 0.234 | [0.227; 0.241] | 1.24 |
| Rai RK et al., 2023(F-Bihar-2016) | 0.598 | [0.562; 0.634] | 1.24 |
| Rai RK et al., 2023(F-Bihar-2019) | 0.738 | [0.705; 0.771] | 1.24 |
| Rai RK et al., 2023(M-Bihar-2016) | 0.286 | [0.257; 0.315] | 1.24 |
| Rai RK et al., 2023(M-Bihar-2019) | 0.307 | [0.277; 0.337] | 1.24 |
| Rai RK et al., 2023(F-Uttar Pradesh-2016) | 0.565 | [0.531; 0.599] | 1.24 |
| Rai RK et al., 2023(F-Uttar Pradesh-2019) | 0.586 | [0.552; 0.620] | 1.24 |
| Rai RK et al., 2023(M-Uttar Pradesh-2016) | 0.369 | [0.337; 0.401] | 1.24 |
| Rai RK et al., 2023(M-Uttar Pradesh-2019) | 0.321 | [0.290; 0.352] | 1.24 |
| Rakesh PS et al., 2019(B) | 0.440 | [0.407; 0.473] | 1.24 |
| Rakesh PS et al., 2019(M) | 0.396 | [0.350; 0.442] | 1.23 |
| Rakesh PS et al., 2019(F) | 0.477 | [0.430; 0.524] | 1.23 |
| Rakesh SR et al., 2014 | 0.723 | [0.710; 0.736] | 1.24 |
| Ramesh Masthi NR et.al., 2012(B) | 0.087 | [0.056; 0.118] | 1.24 |
| Ramesh Masthi NR et.al., 2012(M) | 0.075 | [0.032; 0.118] | 1.23 |
| Ramesh Masthi NR et.al., 2012(F) | 0.097 | [0.053; 0.141] | 1.23 |
| S RP et al., 2015(B) | 0.314 | [0.298; 0.330] | 1.24 |
| S RP et al., 2015(M) | 0.318 | [0.295; 0.341] | 1.24 |
| S RP et al., 2015(F) | 0.311 | [0.288; 0.334] | 1.24 |
| Sahoo J et al., 2021 | 0.455 | [0.424; 0.487] | 1.24 |
| Sarna A et al., 2020 | 0.284 | [0.277; 0.291] | 1.24 |
| Sen A et al., 2006 | 0.670 | [0.619; 0.721] | 1.23 |
| Shanmugam J et al., 2023(B) | 0.886 | [0.873; 0.899] | 1.24 |
| Shanmugam J et al., 2023(F) | 0.502 | [0.472; 0.532] | 1.24 |
| Shanmugam J et al., 2023(M) | 0.498 | [0.470; 0.526] | 1.24 |
| Sharma SK et al., 2012 | 0.715 | [0.702; 0.728] | 1.24 |
| Simhachalam Naidu C.H. et al., 2014 | 0.793 | [0.747; 0.839] | 1.23 |
| Siva PM et al., 2016 | 0.210 | [0.160; 0.260] | 1.23 |
| Srivastava S et al., 2022(F-Bihar2016) | 0.588 | [0.569; 0.607] | 1.24 |
| Srivastava S et al., 2022(F-Bihar-2019) | 0.628 | [0.609; 0.647] | 1.24 |
| Srivastava S et al., 2022(M-Bihar-2016) | 0.327 | [0.303; 0.351] | 1.24 |
| Srivastava S et al., 2022(M-Bihar-2019) | 0.305 | [0.281; 0.329] | 1.24 |
| Srivastava S et al., 2022(F-Uttar Pradesh-2016) | 0.588 | [0.571; 0.605] | 1.24 |
| Srivastava S et al., 2022(F-Uttar Pradesh-2019) | 0.628 | [0.612; 0.644] | 1.24 |
| Srivastava S et al., 2022(M-Uttar Pradesh-2016) | 0.327 | [0.309; 0.345] | 1.24 |
| Srivastava S et al., 2022(M-Uttar Pradesh-2019) | 0.305 | [0.287; 0.323] | 1.24 |
| Subramanian M et al., 2022 | 0.717 | [0.663; 0.771] | 1.23 |
| Sulakshana B et al., 2014 | 0.750 | [0.708; 0.792] | 1.23 |
| Toteja GS et al., 2006 | 0.901 | [0.892; 0.910] | 1.24 |
| Verma K et al., 2022 | 0.563 | [0.524; 0.602] | 1.24 |
| Verma M et al., 1998 | 0.515 | [0.493; 0.537] | 1.24 |
| Wangaskar SA et al., 2021(B) | 0.627 | [0.583; 0.671] | 1.23 |
| Wangaskar SA et al., 2021(F) | 0.618 | [0.567; 0.669] | 1.23 |
| Wangaskar SA et al., 2021(M) | 0.652 | [0.564; 0.740] | 1.20 |
| William R.F et al., 2016 | 0.613 | [0.546; 0.680] | 1.22 |
| theta | 0.510 | [0.457; 0.563] |  |

### Supplementary Table 5: Cumulative meta-analysis of pooled prevalence of anemia- among school children (6-18 years)

| **Study** | **proportion** | **95% CI** |
| --- | --- | --- |
| Adding Basu S et al., 2005(B) [2002] (k=1) | 0.1625 | [0.1409; 0.1841] |
| Adding Basu S et al., 2005(M) [2002] (k=2) | 0.1198 | [0.0360; 0.2036] |
| Adding Basu S et al., 2005(F) [2002] (k=3) | 0.1588 | [0.0675; 0.2501] |
| Adding Manjula AA et al., 2003 [2003] (k=4) | 0.2298 | [0.0770; 0.3825] |
| Adding Muthayya S et al., 2007 [2006] (k=5) | 0.2108 | [0.0871; 0.3345] |
| Adding Biradar S.S et al., 2012 [2008] (k=6) | 0.2441 | [0.1237; 0.3645] |
| Adding Gunjal Sandeep, S et al., 2012(B) [2009] (k=7) | 0.3378 | [0.1284; 0.5473] |
| Adding Gunjal Sandeep, S et al., 2012(M) [2009] (k=8) | 0.4052 | [0.1809; 0.6295] |
| Adding Gunjal Sandeep, S. et al., 2012(F) [2009] (k=9) | 0.4623 | [0.2350; 0.6896] |
| Adding Gupta S et al., 2012(a) [2009] (k=10) | 0.4961 | [0.2822; 0.7100] |
| Adding Simhachalam Naidu C.H et al., 2014 [2010] (k=11) | 0.5230 | [0.3224; 0.7237] |
| Adding Gupta S et al., 2017(b) [2010] (k=12) | 0.5209 | [0.3375; 0.7042] |
| Adding Rakesh SR et al., 2014 [2012] (k=13) | 0.5365 | [0.3651; 0.7078] |
| Adding Ramesh Masthi NR et.al., 2012(B) [2012] (k=14) | 0.5043 | [0.3337; 0.6750] |
| Adding Ramesh Masthi NR et.al., 2012(M) [2012] (k=15) | 0.4758 | [0.3073; 0.6442] |
| Adding Ramesh Masthi NR et.al., 2012(F) [2012] (k=16) | 0.4521 | [0.2878; 0.6164] |
| Adding Siva PM et al., 2016 [2014] (k=17) | 0.4379 | [0.2811; 0.5947] |
| Adding S RP et al., 2015(B) [2014] (k=18) | 0.4310 | [0.2825; 0.5795] |
| Adding S RP et al., 2015(M) [2014] (k=19) | 0.4250 | [0.2841; 0.5659] |
| Adding S RP et al., 2015(F) [2014] (k=20) | 0.4193 | [0.2852; 0.5534] |
| Adding Chauhan S et al., 2022(M-Bihar) [2016] (k=21) | 0.4035 | [0.2722; 0.5347] |
| Adding Chauhan S et al., 2022(F-Bihar) [2016] (k=22) | 0.3942 | [0.2677; 0.5207] |
| Adding Chauhan S et al., 2022(M-Uttar Pradesh) [2016] (k=23) | 0.3808 | [0.2571; 0.5044] |
| Adding Chauhan S et al., 2022(F-Uttar Pradesh) [2016] (k=24) | 0.3732 | [0.2539; 0.4925] |
| Adding Rai RK et al., 2023(M-Bihar-2016) [2016] (k=25) | 0.3697 | [0.2551; 0.4843] |
| Adding Rai RK et al., 2023(F-Bihar-2016) [2016] (k=26) | 0.3785 | [0.2670; 0.4900] |
| Adding Rai RK et al., 2023(M-Uttar Pradesh-2016) [2016] (k=27) | 0.3781 | [0.2709; 0.4854] |
| Adding Rai RK et al., 2023(F-Uttar Pradesh-2016) [2016] (k=28) | 0.3848 | [0.2806; 0.4890] |
| Adding Srivastava S et al., 2022(M-Bihar-2016) [2016] (k=29) | 0.3828 | [0.2822; 0.4834] |
| Adding Srivastava S et al., 2022(F-Bihar2016) [2016] (k=30) | 0.3896 | [0.2915; 0.4878] |
| Adding Srivastava S et al., 2022(M-Uttar Pradesh-2016) [2016] (k=31) | 0.3876 | [0.2926; 0.4826] |
| Adding Srivastava S et al., 2022(F-Uttar Pradesh-2016) [2016] (k=32) | 0.3939 | [0.3011; 0.4867] |
| Adding Kumar et al., 2023 [2016] (k=33) | 0.3904 | [0.3002; 0.4806] |
| Adding Gopalakrishnan S et al., 2018 [2017] (k=34) | 0.4038 | [0.3125; 0.4952] |
| Adding Sarna A et al., 2020 [2018] (k=35) | 0.4004 | [0.3114; 0.4893] |
| Adding Rai RK et al., 2023(M-Bihar-2019) [2019] (k=36) | 0.3978 | [0.3112; 0.4844] |
| Adding Rai RK et al., 2023(F-Bihar-2019) [2019] (k=37) | 0.4070 | [0.3208; 0.4931] |
| Adding Rai RK et al., 2023(M-Uttar Pradesh-2019) [2019] (k=38) | 0.4047 | [0.3208; 0.4887] |
| Adding Rai RK et al., 2023(F-Uttar Pradesh-2019) [2019] (k=39) | 0.4094 | [0.3271; 0.4916] |
| Adding Sahoo J et al., 2021 [2019] (k=40) | 0.4105 | [0.3303; 0.4907] |
| Adding Shanmugam J et al., 2023(M) [2019] (k=41) | 0.4126 | [0.3343; 0.4910] |
| Adding Shanmugam J et al., 2023(B) [2019] (k=42) | 0.4239 | [0.3443; 0.5036] |
| Adding Shanmugam J et al., 2023(F) [2019] (k=43) | 0.4258 | [0.3479; 0.5036] |
| Adding Srivastava S et al., 2022(M-Bihar-2019) [2019] (k=44) | 0.4230 | [0.3468; 0.4992] |
| Adding Srivastava S et al., 2022(F-Bihar-2019) [2019] (k=45) | 0.4276 | [0.3525; 0.5026] |
| Adding Srivastava S et al., 2022(M-Uttar Pradesh-2019) [2019] (k=46) | 0.4249 | [0.3513; 0.4985] |
| Adding Srivastava S et al., 2022(F-Uttar Pradesh-2019) [2019] (k=47) | 0.4292 | [0.3568; 0.5017] |
| Adding Wangaskar SA et al., 2021(M) [2019] (k=48) | 0.4338 | [0.3622; 0.5053] |
| Adding Wangaskar SA et al., 2021(B) [2019] (k=49) | 0.4377 | [0.3672; 0.5082] |
| Adding Wangaskar SA et al., 2021(F) [2019] (k=50) | 0.4413 | [0.3718; 0.5107] |
| Adding Verma K et al., 2022 [2020] (k=51) | 0.4436 | [0.3754; 0.5119] |
| Pooled estimate | 0.4436 | [0.3754; 0.5119] |

### Supplementary Table 6: Subgroup analysis of pooled prevalence of anemia- among school children (6-18 years) based on region

| **Study** | **Effect Size** | **[95% Conf. Interval]** | **% Weight** |
| --- | --- | --- | --- |
| **Group: Central** | | | |
| Chauhan S et al., 2022(F-Uttar Pradesh) | 0.200 | [0.192, 0.208] | 1.24 |
| Chauhan S et al., 2022(M-Uttar Pradesh) | 0.087 | [0.078, 0.096] | 1.24 |
| Jain T et al., 2011 | 0.428 | [0.380, 0.476] | 1.23 |
| Rai RK et al., 2023(F-Uttar Pradesh-2016) | 0.565 | [0.531, 0.599] | 1.24 |
| Rai RK et al., 2023(F-Uttar Pradesh-2019) | 0.586 | [0.552, 0.620] | 1.24 |
| Rai RK et al., 2023(M-Uttar Pradesh-2016) | 0.369 | [0.337, 0.401] | 1.24 |
| Rai RK et al., 2023(M-Uttar Pradesh-2019) | 0.321 | [0.290, 0.352] | 1.24 |
| Srivastava S et al., 2022(F-Uttar Pradesh-2016) | 0.588 | [0.571, 0.605] | 1.24 |
| Srivastava S et al., 2022(F-Uttar Pradesh-2019) | 0.628 | [0.612, 0.644] | 1.24 |
| Srivastava S et al., 2022(M-Uttar Pradesh-2016) | 0.327 | [0.309, 0.345] | 1.24 |
| Srivastava S et al., 2022(M-Uttar Pradesh-2019) | 0.305 | [0.287, 0.323] | 1.24 |
| **Pooled estimate 0.400 [0.296, 0.504]** | | | |
| **Group: East** | | | |
| Behera S et al., 2016(B) | 0.689 | [0.627, 0.751] | 1.22 |
| Behera S et al., 2016(M) | 0.729 | [0.635, 0.823] | 1.2 |
| Behera S et al., 2016(F) | 0.661 | [0.579, 0.743] | 1.21 |
| Bhatia V et al., 2020 | 0.680 | [0.648, 0.712] | 1.24 |
| Bulliyy G et al., 2007 | 0.965 | [0.957, 0.973] | 1.24 |
| Chauhan S et al., 2022(F-Bihar) | 0.200 | [0.189, 0.211] | 1.24 |
| Chauhan S et al., 2022(M-Bihar) | 0.087 | [0.074, 0.100] | 1.24 |
| Gupta S et al., 2012(a) | 0.802 | [0.742, 0.862] | 1.23 |
| Kumari R et al., 2017 | 0.500 | [0.431, 0.569] | 1.22 |
| Rai RK et al., 2023(F-Bihar-2016) | 0.598 | [0.562, 0.634] | 1.24 |
| Rai RK et al., 2023(F-Bihar-2019) | 0.738 | [0.705, 0.771] | 1.24 |
| Rai RK et al., 2023(M-Bihar-2016) | 0.286 | [0.257, 0.315] | 1.24 |
| Rai RK et al., 2023(M-Bihar-2019) | 0.307 | [0.277, 0.337] | 1.24 |
| Sahoo J et al., 2021 | 0.455 | [0.424, 0.487] | 1.24 |
| Srivastava S et al., 2022(F-Bihar2016) | 0.588 | [0.569, 0.607] | 1.24 |
| Srivastava S et al., 2022(F-Bihar-2019) | 0.628 | [0.609, 0.647] | 1.24 |
| Srivastava S et al., 2022(M-Bihar-2016) | 0.327 | [0.303, 0.351] | 1.24 |
| Srivastava S et al., 2022(M-Bihar-2019) | 0.305 | [0.281, 0.329] | 1.24 |
| **Pooled estimate 0.529 [0.420, 0.638]** | | | |
| **Group: North** | | | |
| Banerjee M et al., 2022(B) | 0.344 | [0.321, 0.367] | 1.24 |
| Banerjee M et al., 2022(M) | 0.297 | [0.269, 0.325] | 1.24 |
| Banerjee M et al., 2022(F) | 0.444 | [0.406, 0.482] | 1.24 |
| Basu S et al., 2005(B) | 0.163 | [0.141, 0.184] | 1.24 |
| Basu S et al., 2005(M) | 0.077 | [0.054, 0.100] | 1.24 |
| Basu S et al., 2005(F) | 0.239 | [0.205, 0.273] | 1.24 |
| Goyle A Jr et al., 2009 | 0.963 | [0.928, 0.998] | 1.24 |
| Gupta S et al., 2017(b) | 0.497 | [0.430, 0.564] | 1.22 |
| Kamble BD et al., 2021 | 0.590 | [0.522, 0.658] | 1.22 |
| Subramanian M et al., 2022 | 0.717 | [0.663, 0.771] | 1.23 |
| Verma K et al., 2022 | 0.563 | [0.524, 0.602] | 1.24 |
| Verma M et al., 1998 | 0.515 | [0.493, 0.537] | 1.24 |
| **Pooled estimate 0.450 [0.310, 0.590]** | | | |
| **Group: South** | | | |
| Biradar S.S et al., 2012 | 0.411 | [0.378, 0.444] | 1.24 |
| Chandrakumari AS et al., 2019 | 0.486 | [0.421, 0.552] | 1.22 |
| Gopalakrishnan S et al., 2018 | 0.848 | [0.803, 0.893] | 1.23 |
| Jagadish Kumar K et al., 2017(B) | 0.525 | [0.456, 0.594] | 1.22 |
| Jagadish Kumar K et al., 2017(M) | 0.525 | [0.447, 0.603] | 1.21 |
| Jagadish Kumar K et al., 2017(F) | 0.522 | [0.378, 0.666] | 1.14 |
| Kumar et al., 2023 | 0.279 | [0.234, 0.323] | 1.23 |
| Manjula AA et al., 2003 | 0.442 | [0.409, 0.475] | 1.24 |
| Muthayya S et al., 2007 | 0.136 | [0.121, 0.151] | 1.24 |
| Prabhakar SCJ et al., 2009 | 0.777 | [0.715, 0.839] | 1.22 |
| Rakesh PS et al., 2019(B) | 0.440 | [0.407, 0.473] | 1.24 |
| Rakesh PS et al., 2019(M) | 0.396 | [0.350, 0.442] | 1.23 |
| Rakesh PS et al., 2019(F) | 0.477 | [0.430, 0.524] | 1.23 |
| Rakesh SR et al., 2014 | 0.723 | [0.710, 0.736] | 1.24 |
| Ramesh Masthi NR et.al., 2012(B) | 0.087 | [0.056, 0.118] | 1.24 |
| Ramesh Masthi NR et.al., 2012(M) | 0.075 | [0.032, 0.118] | 1.24 |
| Ramesh Masthi NR et.al., 2012(F) | 0.097 | [0.053, 0.141] | 1.23 |
| S RP et al., 2015(B) | 0.314 | [0.298, 0.330] | 1.24 |
| S RP et al., 2015(M) | 0.318 | [0.295, 0.341] | 1.24 |
| S RP et al., 2015(F) | 0.311 | [0.288, 0.334] | 1.24 |
| Shanmugam J et al., 2023(B) | 0.886 | [0.873, 0.899] | 1.24 |
| Shanmugam J et al., 2023(F) | 0.502 | [0.472, 0.532] | 1.24 |
| Shanmugam J et al., 2023(M) | 0.498 | [0.470, 0.526] | 1.24 |
| Simhachalam Naidu C.H et al., 2014 | 0.793 | [0.747, 0.839] | 1.23 |
| Siva PM et al., 2016 | 0.210 | [0.160, 0.260] | 1.23 |
| Sulakshana B et al., 2014 | 0.750 | [0.708, 0.792] | 1.24 |
| Wangaskar SA et al., 2021(B) | 0.627 | [0.583, 0.671] | 1.23 |
| Wangaskar SA et al., 2021(F) | 0.618 | [0.567, 0.669] | 1.23 |
| Wangaskar SA et al., 2021(M) | 0.652 | [0.564, 0.740] | 1.2 |
| William R.F et al., 2016 | 0.613 | [0.546, 0.680] | 1.22 |
| **Pooled estimate 0.477 [0.395, 0.559]** | | | |
| **Group: Northeast** | | | |
| Mahanta T.G et al., 2015 | 0.963 | [0.950, 0.976] | 1.24 |
| Sharma SK et al., 2012 | 0.715 | [0.702, 0.728] | 1.24 |
| **Pooled estimate 0.839 [0.596, 1.082]** | | | |
| **Group: West** | | | |
| Ahankari AS et.al., 2017 | 0.870 | [0.849, 0.891] | 1.24 |
| Arlappa N et al., 2014(c) | 0.611 | [0.578, 0.644] | 1.24 |
| Chaudhary SM et al., 2008 | 0.351 | [0.297, 0.405] | 1.23 |
| Gunjal Sandeep, S et al., 2012(B) | 0.897 | [0.878, 0.917] | 1.24 |
| Gunjal Sandeep, S et al., 2012(M) | 0.877 | [0.847, 0.907] | 1.24 |
| Gunjal Sandeep, S et al., 2012(F) | 0.919 | [0.894, 0.944] | 1.24 |
| Nair et al., 2023 | 0.657 | [0.612, 0.702] | 1.23 |
| Sen A et al., 2006 | 0.670 | [0.619, 0.721] | 1.23 |
| **Pooled estimate 0.733 [0.597, 0.869]** | | | |
| **Overall Pooled estimate 0.509 [0.456, 0.561]** | | | |
| Heterogeneity summary  Test of group differences: Qb = chi2(5) = 23.01 Prob > Qb = 0.000 | | | |

### Supplementary Table 7: Subgroup analysis of pooled prevalence of anemia- among school children (6-18 years) based on states and union territories

| **Study** | **Effect Size** | **[95% Conf. Interval]** | **% Weight** |
| --- | --- | --- | --- |
| **Group: Andhra Pradesh** | | | |
| Kumar et al., 2023 | 0.279 | [0.234, 0.323] | 1.23 |
| **Pooled estimate 0.279 [0.234, 0.323]** | | | |
| **Group: Assam** | | | |
| Mahanta T.G et al., 2015 | 0.963 | [0.950, 0.976] | 1.24 |
| Sharma SK et al., 2012 | 0.715 | [0.702, 0.728] | 1.24 |
| **Pooled estimate 0.839 [0.596, 1.082]** | | | |
| **Group: Bihar** | | | |
| Chauhan S et al., 2022(F-Bihar) | 0.2 | [0.189, 0.211] | 1.24 |
| Chauhan S et al., 2022(M-Bihar) | 0.087 | [0.074, 0.1] | 1.24 |
| Kumari R et al., 2017 | 0.5 | [0.431, 0.569] | 1.22 |
| Rai RK et al., 2023(F-Bihar-2016) | 0.598 | [0.562, 0.634] | 1.24 |
| Rai RK et al., 2023(F-Bihar-2019) | 0.738 | [0.705, 0.771] | 1.24 |
| Rai RK et al., 2023(M-Bihar-2016) | 0.286 | [0.257, 0.315] | 1.24 |
| Rai RK et al., 2023(M-Bihar-2019) | 0.307 | [0.277, 0.337] | 1.24 |
| Srivastava S et al., 2022(F-Bihar2016) | 0.588 | [0.569, 0.607] | 1.24 |
| Srivastava S et al., 2022(F-Bihar-2019) | 0.628 | [0.609, 0.647] | 1.24 |
| Srivastava S et al., 2022(M-Bihar-2016) | 0.327 | [0.303, 0.351] | 1.24 |
| Srivastava S et al., 2022(M-Bihar-2019) | 0.305 | [0.281, 0.329] | 1.24 |
| **Pooled estimate 0.415 [0.293, 0.536]** | | | |
| **Group: Delhi** | | | |
| Kamble BD et al., 2021 | 0.590 | [0.522, 0.658] | 1.22 |
| **Pooled estimate 0.590 [0.522, 0.658]** | | | |
| **Group: Gujarat** | | | |
| Sen A et al., 2006 | 0.670 | [0.619, 0.721] | 1.23 |
| **Pooled estimate 0.670 [0.619, 0.721]** | | | |
| **Group: Haryana** | | | |
| Subramanian M et al., 2022 | 0.717 | [0.663, 0.771] | 1.23 |
| **Pooled estimate 0.717 [0.663, 0.771]** | | | |
| **Group: Jammu & Kashmir** | | | |
| Banerjee M et al., 2022(B) | 0.344 | [0.321, 0.367] | 1.24 |
| Banerjee M et al., 2022(M) | 0.297 | [0.269, 0.325] | 1.24 |
| Banerjee M et al., 2022(F) | 0.444 | [0.406, 0.482] | 1.24 |
| Gupta S et al., 2017(b) | 0.497 | [0.430, 0.564] | 1.22 |
| **Pooled estimate 0.392 [0.304, 0.480]** | | | |
| **Group: Karnataka** | | | |
| Biradar S.S et al., 2012 | 0.411 | [0.378, 0.444] | 1.24 |
| Jagadish Kumar K et al., 2017(B) | 0.525 | [0.456, 0.594] | 1.22 |
| Jagadish Kumar K et al., 2017(M) | 0.525 | [0.447, 0.603] | 1.21 |
| Jagadish Kumar K et al., 2017(F) | 0.522 | [0.378, 0.666] | 1.14 |
| Muthayya S et al., 2007 | 0.136 | [0.121, 0.151] | 1.24 |
| Prabhakar SCJ et al., 2009 | 0.777 | [0.715, 0.839] | 1.22 |
| Ramesh Masthi NR et.al 2012(B) | 0.087 | [0.056, 0.118] | 1.24 |
| Ramesh Masthi NR et.al 2012(M) | 0.075 | [0.032, 0.118] | 1.24 |
| Ramesh Masthi NR et.al 2012(F) | 0.097 | [0.053, 0.141] | 1.23 |
| Sulakshana B et al., 2014 | 0.750 | [0.708, 0.792] | 1.24 |
| **Pooled estimate 0.389 [0.218, 0.56]** | | | |
| **Group: Kerala** | | | |
| Manjula AA et al., 2003 | 0.442 | [0.409, 0.475] | 1.24 |
| Rakesh PS et al., 2019(B) | 0.44 | [0.407, 0.473] | 1.24 |
| Rakesh PS et al., 2019(M) | 0.396 | [0.35, 0.442] | 1.23 |
| Rakesh PS et al., 2019(F) | 0.477 | [0.43, 0.524] | 1.23 |
| Rakesh SR et al., 2014 | 0.723 | [0.71, 0.736] | 1.24 |
| S RP et al., 2015(B) | 0.314 | [0.298, 0.33] | 1.24 |
| S RP et al., 2015(M) | 0.318 | [0.295, 0.341] | 1.24 |
| S RP et al., 2015(F) | 0.311 | [0.288, 0.334] | 1.24 |
| Siva PM et al., 2016 | 0.210 | [0.160, 0.260] | 1.23 |
| **Pooled estimate 0.404 [0.308, 0.5]** | | | |
| **Group: Maharashtra** | | | |
| Ahankari AS et.al 2017 | 0.87 | [0.849, 0.891] | 1.24 |
| Arlappa N. et al., 2014(c) | 0.611 | [0.578, 0.644] | 1.24 |
| Chaudhary SM et al., 2008 | 0.351 | [0.297, 0.405] | 1.23 |
| Gunjal Sandeep, S et al., 2012(B) | 0.897 | [0.878, 0.917] | 1.24 |
| Gunjal Sandeep, S et al., 2012(M) | 0.877 | [0.847, 0.907] | 1.24 |
| Gunjal Sandeep, S et al., 2012(F) | 0.919 | [0.894, 0.944] | 1.24 |
| Nair et al., 2023 | 0.657 | [0.612, 0.702] | 1.23 |
| **Pooled estimate 0.741 [0.586, 0.897]** | | | |
| **Group: Pondicherry** | | | |
| Wangaskar SA et al., 2021(B) | 0.627 | [0.583, 0.671] | 1.23 |
| Wangaskar SA et al., 2021(F) | 0.618 | [0.567, 0.669] | 1.23 |
| Wangaskar SA et al., 2021(M) | 0.652 | [0.564, 0.740] | 1.2 |
| **Pooled estimate 0.627 [0.595, 0.658]** | | | |
| **Group: Rajasthan** | | | |
| Goyle A Jr et al., 2009 | 0.963 | [0.928, 0.998] | 1.24 |
| Verma K et al., 2022 | 0.563 | [0.524, 0.602] | 1.24 |
| **Pooled estimate 0.763 [0.371, 1.155]** | | | |
| **Group: Tamil Nadu** | | | |
| Chandrakumari AS et al., 2019 | 0.486 | [0.421, 0.552] | 1.22 |
| Gopalakrishnan S et al., 2018 | 0.848 | [0.803, 0.893] | 1.23 |
| Shanmugam J et al., 2023(B) | 0.886 | [0.873, 0.899] | 1.24 |
| Shanmugam J et al., 2023(F) | 0.502 | [0.472, 0.532] | 1.24 |
| Shanmugam J et al., 2023(M) | 0.498 | [0.47, 0.526] | 1.24 |
| William R.F et al., 2016 | 0.613 | [0.546, 0.68] | 1.22 |
| **Pooled estimate 0.640 [0.492, 0.787]** | | | |
| **Group: Telangana** | | | |
| Simhachalam Naidu C.H et al., 2014 | 0.793 | [0.747, 0.839] | 1.23 |
| **Pooled estimate 0.793 [0.747, 0.839]** | | | |
| **Group: Uttar Pradesh** | | | |
| Chauhan S et al., 2022(F-Uttar Pradesh) | 0.2 | [0.192, 0.208] | 1.25 |
| Chauhan S et al., 2022(M-Uttar Pradesh) | 0.087 | [0.078, 0.096] | 1.25 |
| Jain T et al., 2011 | 0.428 | [0.38, 0.476] | 1.23 |
| Rai RK et al., 2023(F-Uttar Pradesh-2016) | 0.565 | [0.531, 0.599] | 1.24 |
| Rai RK et al., 2023(F-Uttar Pradesh-2019) | 0.586 | [0.552, 0.62] | 1.24 |
| Rai RK et al., 2023(M-Uttar Pradesh-2016) | 0.369 | [0.337, 0.401] | 1.24 |
| Rai RK et al., 2023(M-Uttar Pradesh-2019) | 0.321 | [0.29, 0.352] | 1.24 |
| Srivastava S et al., 2022(F-Uttar Pradesh-2016) | 0.588 | [0.571, 0.605] | 1.24 |
| Srivastava S et al., 2022(F-Uttar Pradesh-2019) | 0.628 | [0.612, 0.644] | 1.24 |
| Srivastava S et al., 2022(M-Uttar Pradesh-2016) | 0.327 | [0.309, 0.345] | 1.24 |
| Srivastava S et al., 2022(M-Uttar Pradesh-2019) | 0.305 | [0.287, 0.323] | 1.24 |
| **Pooled estimate 0.400 [0.296, 0.504]** | | | |
| **Group: Chandigarh** | | | |
| Basu S et al., 2005(B) | 0.163 | [0.141, 0.184] | 1.24 |
| Basu S et al., 2005(M) | 0.077 | [0.054, 0.100] | 1.24 |
| Basu S et al., 2005(F) | 0.239 | [0.205, 0.273] | 1.24 |
| **Pooled estimate 0.159 [0.068, 0.25]** | | | |
| **Group: Orissa** | | | |
| Behera S et al., 2016(B) | 0.689 | [0.627, 0.751] | 1.22 |
| Behera S et al., 2016(M) | 0.729 | [0.635, 0.823] | 1.2 |
| Behera S et al., 2016(F) | 0.661 | [0.579, 0.743] | 1.21 |
| Bhatia V et al., 2020 | 0.68 | [0.648, 0.712] | 1.24 |
| Bulliyy G et al., 2007 | 0.965 | [0.957, 0.973] | 1.25 |
| Sahoo J et al., 2021 | 0.455 | [0.424, 0.487] | 1.24 |
| **Pooled estimate 0.697 [0.563, 0.831]** | | | |
| **Group: Punjab** | | | |
| Verma M et al., 1998 | 0.515 | [0.493, 0.537] | 1.24 |
| **Pooled estimate 0.515 [0.493, 0.537]** | | | |
| **Group: West Bengal** | | | |
| Gupta S et al., 2012(a) | 0.802 | [0.742, 0.862] | 1.23 |
| **Pooled estimate 0.802 [0.742, 0.862]** | | | |
| **Overall Pooled estimate 0.509 [0.456, 0.561]** | | | |
| Heterogeneity Summary  Test of group differences: Qb = chi2 (18) = 532.16 Prob > Qb = 0.000 | | | |

### Supplementary Table 8: Subgroup analysis of pooled prevalence of anemia- among school children (6-18 years) based on gender

| **Study** | **Effect Size** | **[95% Conf. Interval]** | **% Weight** |
| --- | --- | --- | --- |
| **Group: both male & females** | | | |
| Banerjee M et al., 2022 | 0.344 | [0.321, 0.367] | 1.18 |
| Basu S et al., 2005 | 0.163 | [0.141, 0.184] | 1.18 |
| Behera S et al., 2016 | 0.689 | [0.627, 0.751] | 1.17 |
| Gunjal Sandeep, S et al., 2012 | 0.897 | [0.878, 0.917] | 1.18 |
| Gupta S et al., 2012(a) | 0.802 | [0.742, 0.862] | 1.17 |
| Gupta S. et al., 2017(b) | 0.497 | [0.43, 0.564] | 1.16 |
| Jagadish Kumar K. et al., 2017 | 0.525 | [0.456, 0.594] | 1.16 |
| Manjula AA et al., 2003 | 0.442 | [0.409, 0.475] | 1.18 |
| Muthayya S et al., 2007 | 0.136 | [0.121, 0.151] | 1.18 |
| Prabhakar SCJ et al., 2009 | 0.777 | [0.715, 0.839] | 1.17 |
| Rahman MHU et al., 2020 | 0.234 | [0.227, 0.241] | 1.19 |
| Rakesh PS et al., 2019 | 0.44 | [0.407, 0.473] | 1.18 |
| Rakesh SR et al., 2014 | 0.723 | [0.71, 0.736] | 1.19 |
| Ramesh Masthi NR et.al., 2012 | 0.087 | [0.056, 0.118] | 1.18 |
| S RP et al., 2015 | 0.314 | [0.298, 0.33] | 1.18 |
| Sahoo J et al., 2021 | 0.455 | [0.424, 0.487] | 1.18 |
| Sarna A et al., 2020 | 0.284 | [0.277, 0.291] | 1.19 |
| Shanmugam J et al., 2023 | 0.886 | [0.873, 0.899] | 1.19 |
| Verma M et al., 1998 | 0.515 | [0.493, 0.537] | 1.18 |
| Wangaskar SA et al., 2021 | 0.627 | [0.583, 0.671] | 1.18 |
| **Pooled estimate 0.491 [0.382, 0.601]** | | | |
| **Group: Female** | | | |
| Ahankari AS et.al., 2017 | 0.87 | [0.849, 0.891] | 1.18 |
| Arlappa N. et al., 2014(c) | 0.611 | [0.578, 0.644] | 1.18 |
| Banerjee M et al., 2022 | 0.444 | [0.406, 0.482] | 1.18 |
| Basu S et al., 2005 | 0.239 | [0.205, 0.273] | 1.18 |
| Behera S et al., 2016 | 0.661 | [0.579, 0.743] | 1.15 |
| Bharati P et al., 2009(b) | 0.9 | [0.899, 0.901] | 1.19 |
| Bhatia V et al., 2020 | 0.68 | [0.648, 0.712] | 1.18 |
| Biradar S.S et al., 2012 | 0.411 | [0.378, 0.444] | 1.18 |
| Bulliyy G et al., 2007 | 0.965 | [0.957, 0.973] | 1.19 |
| Chandrakumari AS et al., 2019 | 0.486 | [0.421, 0.552] | 1.16 |
| Chaudhary SM et al., 2008 | 0.351 | [0.297, 0.405] | 1.17 |
| Chauhan S et al., 2022(Bihar) | 0.2 | [0.189, 0.211] | 1.19 |
| Chauhan S et al., 2022(Uttar Pradesh) | 0.2 | [0.192, 0.208] | 1.19 |
| Gopalakrishnan S et al., 2018 | 0.848 | [0.803, 0.893] | 1.18 |
| Goyle A Jr et al., 2009 | 0.963 | [0.928, 0.998] | 1.18 |
| Gunjal Sandeep, S et al., 2012 | 0.919 | [0.894, 0.944] | 1.18 |
| Jagadish Kumar K et al 2017 | 0.522 | [0.378, 0.666] | 1.09 |
| Kamble BD et al., 2021 | 0.59 | [0.522, 0.658] | 1.16 |
| Kumari R et al., 2017 | 0.5 | [0.431, 0.569] | 1.16 |
| Mahanta T.G et al., 2015 | 0.963 | [0.95, 0.976] | 1.19 |
| Nair et al., 2023 | 0.657 | [0.612, 0.702] | 1.18 |
| Rai RK et al., 2023(Bihar-2016) | 0.598 | [0.562, 0.634] | 1.18 |
| Rai RK et al., 2023(Bihar-2019) | 0.738 | [0.705, 0.771] | 1.18 |
| Rai RK et al., 2023(Uttar Pradesh-2016) | 0.565 | [0.531, 0.599] | 1.18 |
| Rai RK et al., 2023(Uttar Pradesh-2019) | 0.586 | [0.552, 0.62] | 1.18 |
| Rakesh PS et al., 2019 | 0.477 | [0.43, 0.524] | 1.17 |
| Ramesh Masthi NR et.al 2012 | 0.097 | [0.053, 0.141] | 1.18 |
| S RP et al., 2015 | 0.311 | [0.288, 0.334] | 1.18 |
| Sen A et al., 2006 | 0.67 | [0.619, 0.721] | 1.17 |
| Shanmugam J et al., 2023 | 0.502 | [0.472, 0.532] | 1.18 |
| Sharma SK et al., 2012 | 0.715 | [0.702, 0.728] | 1.19 |
| Simhachalam Naidu C.H. et al., 2014 | 0.793 | [0.747, 0.839] | 1.18 |
| Siva PM et al., 2016 | 0.21 | [0.16, 0.26] | 1.17 |
| Srivastava S et al., 2022(Bihar2016) | 0.588 | [0.569, 0.607] | 1.18 |
| Srivastava S et al., 2022(Bihar-2019) | 0.628 | [0.609, 0.647] | 1.18 |
| Srivastava S et al., 2022(Uttar Pradesh-2016) | 0.588 | [0.571, 0.605] | 1.18 |
| Srivastava S et al., 2022(Uttar Pradesh-2019) | 0.628 | [0.612, 0.644] | 1.18 |
| Subramanian M et al., 2022 | 0.717 | [0.663, 0.771] | 1.17 |
| Sulakshana B et al., 2014 | 0.75 | [0.708, 0.792] | 1.18 |
| Toteja GS et al., 2006 | 0.901 | [0.892, 0.91] | 1.19 |
| Verma K et al., 2022 | 0.563 | [0.524, 0.602] | 1.18 |
| Wangaskar SA et al., 2021 | 0.618 | [0.567, 0.669] | 1.17 |
| William R.F et al., 2016 | 0.613 | [0.546, 0.680] | 1.16 |
| **Pooled estimate 0.601 [0.535, 0.668]** | | | |
| **Group: Male** | | | |
| Banerjee M et al., 2022 | 0.297 | [0.269, 0.325] | 1.18 |
| Basu S et al., 2005 | 0.077 | [0.054, 0.1] | 1.18 |
| Behera S et al., 2016 | 0.729 | [0.635, 0.823] | 1.14 |
| Chauhan S et al., 2022(Bihar) | 0.087 | [0.074, 0.1] | 1.19 |
| Chauhan S et al., 2022(Uttar Pradesh) | 0.087 | [0.078, 0.096] | 1.19 |
| Gunjal Sandeep, S et al., 2012 | 0.877 | [0.847, 0.907] | 1.18 |
| Jagadish Kumar K. et al., 2017 | 0.525 | [0.447, 0.603] | 1.16 |
| Jain T et al., 2011 | 0.428 | [0.38, 0.476] | 1.17 |
| Kumar et al., 2023 | 0.279 | [0.234, 0.323] | 1.18 |
| Rai RK et al., 2023(Bihar-2016) | 0.286 | [0.257, 0.315] | 1.18 |
| Rai RK et al., 2023(Bihar-2019) | 0.307 | [0.277, 0.337] | 1.18 |
| Rai RK et al., 2023(Uttar Pradesh-2016) | 0.369 | [0.337, 0.401] | 1.18 |
| Rai RK et al., 2023(Uttar Pradesh-2019) | 0.321 | [0.29, 0.352] | 1.18 |
| Rakesh PS et al., 2019 | 0.396 | [0.35, 0.442] | 1.18 |
| Ramesh Masthi NR et.al 2012 | 0.075 | [0.032, 0.118] | 1.18 |
| S RP et al., 2015 | 0.318 | [0.295, 0.341] | 1.18 |
| Shanmugam J et al., 2023 | 0.498 | [0.47, 0.526] | 1.18 |
| Srivastava S et al., 2022(Bihar-2016) | 0.327 | [0.303, 0.351] | 1.18 |
| Srivastava S et al., 2022(Bihar-2019) | 0.305 | [0.281, 0.329] | 1.18 |
| Srivastava S et al., 2022(Uttar Pradesh-2016) | 0.327 | [0.309, 0.345] | 1.18 |
| Srivastava S et al., 2022(Uttar Pradesh-2019) | 0.305 | [0.287, 0.323] | 1.18 |
| Wangaskar SA et al., 2021 | 0.652 | [0.564, 0.740] | 1.15 |
| **Pooled estimate 0.356 [0.271, 0.441]** | | | |
| **Overall Pooled estimate 0.512 [0.460, 0.564]** | | | |
| Heterogeneity summary  Test of group differences: Qb = chi2(2) = 19.89 Prob > Qb = 0.000 | | | |

### Supplementary Figure 10: Sensitivity analysis of pooled prevalence of anemia- among adults (19-59 years)

###

### Supplementary Figure 11: Cumulative meta-analysis of pooled prevalence of anemia- among adults (19-59 years)


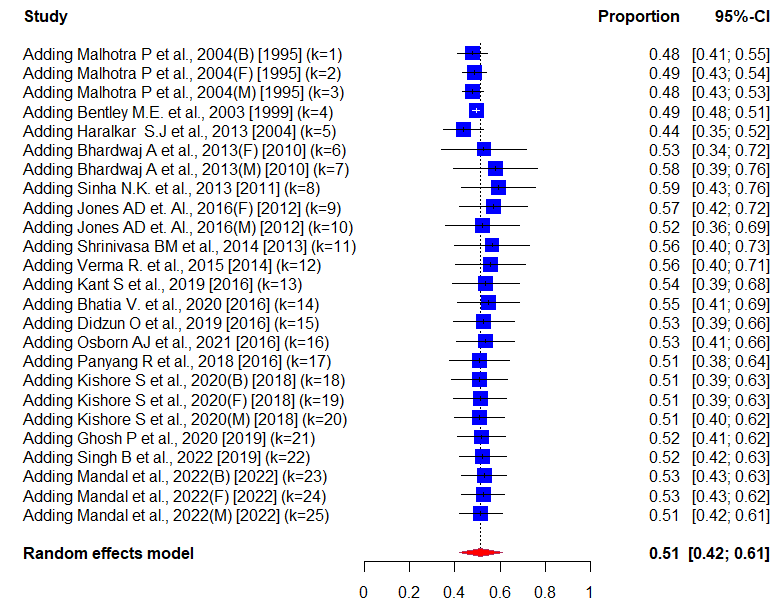


### Supplementary Figure 12: Subgroup analysis of pooled prevalence of anemia- among adults (19-59 years) based on region

### Supplementary Table 9: Subgroup analysis of pooled prevalence of anemia- among adults (19-59 years) based on states and union territories

| **Study** | **Effect Size** | **[95% Conf. Interval]** | **% Weight** |
| --- | --- | --- | --- |
| **Group: Andhra Pradesh** | | | |
| Bentley M.E et al., 2003 | 0.495 | [0.480, 0.510] | 1.98 |
| Finkelstein JL et al., 2021(b) | 0.415 | [0.384, 0.446] | 1.98 |
| Jones AD et al., 2016(F) | 0.400 | [0.382, 0.418] | 1.98 |
| Jones AD et al., 2016(M) | 0.100 | [0.090, 0.110] | 1.99 |
| **Pooled estimate 0.352 [0.182, 0.523]** | | | |
| **Group: Assam** | | | |
| Panyang R et al., 2018 | 0.084 | [0.064, 0.104] | 1.98 |
| **Pooled estimate 0.084 [0.064, 0.104]** | | | |
| **Group: Bihar** | | | |
| Singh R.K 2013 (a) | 0.682 | [0.666, 0.698] | 1.98 |
| **Pooled estimate 0.682 [0.666, 0.698]** | | | |
| **Group: Chattisgarh** | | | |
| Singh R.K 2013 (a) | 0.571 | [0.555, 0.587] | 1.98 |
| **Pooled estimate 0.571 [0.555, 0.587]** | | | |
| **Group: Haryana** | | | |
| Kant S et al., 2019 | 0.279 | [0.254, 0.304] | 1.98 |
| Malhotra P et al., 2004(B) | 0.479 | [0.412, 0.546] | 1.93 |
| Malhotra P et al., 2004(F) | 0.5 | [0.416, 0.584] | 1.9 |
| Malhotra P et al., 2004(M) | 0.443 | [0.333, 0.553] | 1.85 |
| Verma R et al., 2015 | 0.489 | [0.478, 0.500] | 1.99 |
| **Pooled estimate 0.435 [0.348, 0.522]** | | | |
| **Group: Himachal Pradesh** | | | |
| Bhardwaj A et al., 2013(F) | 0.967 | [0.952, 0.982] | 1.99 |
| Bhardwaj A et al., 2013(M) | 0.872 | [0.837, 0.907] | 1.97 |
| **Pooled estimate 0.921 [0.828, 1.014]** | | | |
| **Group: Jharkhand** | | | |
| Singh R.K 2013 (a) | 0.694 | [0.676, 0.712] | 1.98 |
| **Pooled estimate 0.694 [0.676, 0.712]** | | | |
| **Group: Karnataka** | | | |
| Kamath R et al., 2013 | 0.559 | [0.484, 0.634] | 1.92 |
| Thankachan P et al., 2007 | 0.390 | [0.294, 0.486] | 1.88 |
| **Pooled estimate 0.477 [0.312, 0.643]** | | | |
| **Group: Kerala** | | | |
| Rohisha IK et al., 2019 | 0.890 | [0.861, 0.919] | 1.98 |
| Shrinivasa BM et al., 2014 | 0.965 | [0.946, 0.984] | 1.98 |
| **Pooled estimate 0.928 [0.855, 1.002]** | | | |
| **Group: Madhya Pradesh** | | | |
| Singh R.K 2013 (a) | 0.558 | [0.545, 0.571] | 1.99 |
| **Pooled estimate 0.558 [0.545, 0.571]** | | | |
| **Group: Maharashtra** | | | |
| Haralkar S.J et al., 2013 | 0.276 | [0.238, 0.314] | 1.97 |
| Rao S et al., 2011 | 0.770 | [0.730, 0.810] | 1.97 |
| Singh G et.al 2017 | 0.721 | [0.642, 0.801] | 1.91 |
| **Pooled estimate 0.588 [0.279, 0.897]** | | | |
| **Group: Meghalaya** | | | |
| Dey S et al., 2010 | 0.496 | [0.48, 0.512] | 1.98 |
| **Pooled estimate 0.496 [0.48, 0.512]** | | | |
| **Group: Nagaland** | | | |
| Meshram II et al., 2020(a) | 0.402 | [0.344, 0.46] | 1.95 |
| **Pooled estimate 0.402 [0.344, 0.46]** | | | |
| **Group: Rajasthan** | | | |
| Singh R.K 2013 (a) | 0.526 | [0.510, 0.542] | 1.98 |
| **Pooled estimate 0.526 [0.510, 0.542]** | | | |
| **Group: Tamil Nadu** | | | |
| Kandasamy K et al., 2017(B) | 0.730 | [0.668, 0.792] | 1.94 |
| Kandasamy K et al., 2017(F) | 0.470 | [0.379, 0.561] | 1.89 |
| Kandasamy K et al., 2017(M) | 0.260 | [0.166, 0.354] | 1.88 |
| Little M et al., 2018(F) | 0.572 | [0.524, 0.62] | 1.96 |
| Little M et al., 2018(M) | 0.352 | [0.301, 0.403] | 1.96 |
| Osborn AJ et al., 2021 | 0.648 | [0.603, 0.693] | 1.96 |
| **Pooled estimate 0.507 [0.365, 0.65]** | | | |
| **Group: Uttar Pradesh** | | | |
| Seth R.K et al., 2015 | 0.490 | [0.443, 0.537] | 1.96 |
| Singh B et al., 2022 | 0.643 | [0.598, 0.688] | 1.96 |
| Singh R.K 2013 (a) | 0.497 | [0.487, 0.507] | 1.99 |
| **Pooled estimate 0.542 [0.446, 0.639]** | | | |
| **Group: Uttarakhand** | | | |
| Kishore S et al., 2020(B) | 0.532 | [0.519, 0.545] | 1.99 |
| Kishore S et al., 2020(F) | 0.546 | [0.532, 0.560] | 1.99 |
| Kishore S et al., 2020(M) | 0.451 | [0.417, 0.485] | 1.97 |
| Singh R.K 2013 (a) | 0.553 | [0.534, 0.572] | 1.98 |
| **Pooled estimate 0.522 [0.479, 0.566]** | | | |
| **Group: Manipur** | | | |
| Shimrah C et al., 2022 | 0.568 | [0.507, 0.629] | 1.94 |
| **Pooled estimate 0.568 [0.507, 0.629]** | | | |
| **Group: Orissa** | | | |
| Bhatia V et al., 2020 | 0.710 | [0.679, 0.741] | 1.98 |
| Singh R.K 2013 (a) | 0.609 | [0.594, 0.624] | 1.99 |
| **Pooled estimate 0.659 [0.56, 0.757]** | | | |
| **Group: Punjab** | | | |
| Gupta V.K et al., 2011(F) | 0.886 | [0.864, 0.907] | 1.99 |
| **Pooled estimate 0.886 [0.864, 0.907]** | | | |
| **Group: West Bengal** | | | |
| Chowdhury T.K et al., 2019 | 0.8 | [0.755, 0.845] | 1.96 |
| Ghosh P et al., 2020 | 0.708 | [0.627, 0.789] | 1.91 |
| Jana A et al., 2022 | 0.64 | [0.633, 0.647] | 1.99 |
| Mandal et al., 2022(B) | 0.675 | [0.644, 0.705] | 1.98 |
| Mandal et al., 2022(F) | 0.427 | [0.385, 0.47] | 1.97 |
| Mandal et al., 2022(M) | 0.247 | [0.204, 0.291] | 1.96 |
| Sinha N.K. et al., 2013 | 0.697 | [0.639, 0.755] | 1.95 |
| **Pooled estimate 0.599 [0.456, 0.741]** | | | |
| **Overall Pooled estimate 0.562 [0.506, 0.618]** | | | |
| Heterogeneity summary  Test of group differences: Qb = chi2(20) = 7187.44 Prob > Qb = 0.000 | | | |

### Supplementary Figure 13: Subgroup analysis of pooled prevalence of anemia- among adults (19-59 years) based on gender

### Supplementary Figure 14: Sensitivity analysis of pooled prevalence of anemia- among elderly persons (≥60 years)

###

### Supplementary Figure 15: Cumulative meta-analysis of pooled prevalence of anemia- among elderly persons (≥60 years)


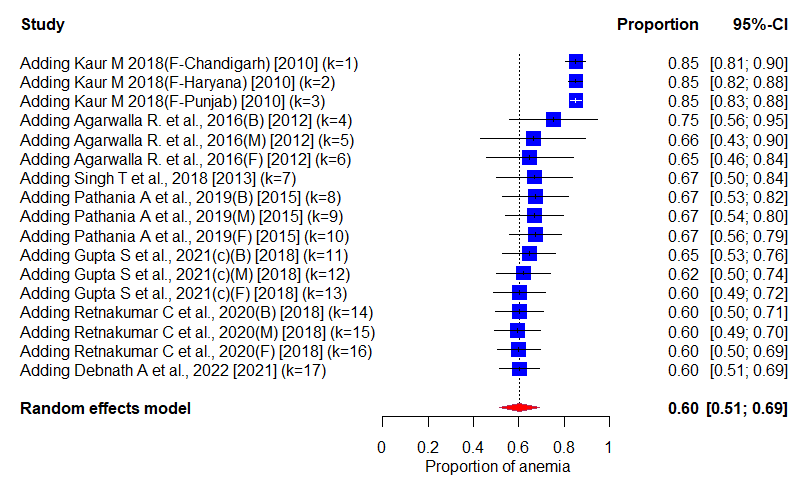


### Supplementary Figure 16: Subgroup analysis of pooled prevalence of anemia- among elderly persons (≥60 years) based on region

### Supplementary Figure 17: Subgroup analysis of pooled prevalence of anemia- among elderly persons (≥60 years) based on states & union territories

### Supplementary Figure 18: Subgroup analysis of pooled prevalence of anemia- among elderly persons (≥60 years) based on gender

### Supplementary Figure 19: Cumulative meta-analysis of pooled prevalence of anemia- among pregnant women

**
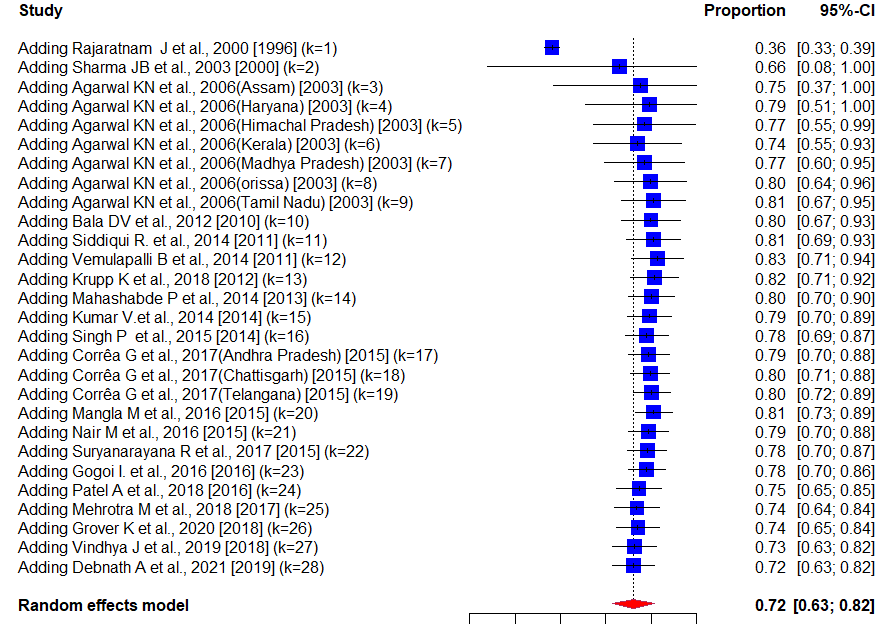
**

### Supplementary Figure 20: Subgroup analysis of pooled prevalence of anemia- among pregnant women based on region

### Supplementary Table 10: Subgroup analysis of pooled prevalence of anemia- among pregnant women based on states & union territories

| **Study** | **Effect Size** | **[95% Conf. Interval]** | **% Weight** |
| --- | --- | --- | --- |
| **Group: Andaman & Nicobar** |  |  |  |
| Mehrotra M et al., 2018 | 0.509 | [0.474, 0.544] | 2.65 |
| **Pooled estimate 0.509 [0.474, 0.544]** | | | |
| **Group: Andhra Pradesh** | | | |
| Corrêa G et al., 2017 | 0.924 | [0.902, 0.946] | 2.66 |
| Vemulapalli B et al., 2014 | 1.000 | [0.999, 1.001] | 2.66 |
| **Pooled estimate 0.963 [0.888, 1.037]** | | | |
| **Group: Assam** | | | |
| Agarwal KN et al., 2006 | 0.939 | [0.898, 0.980] | 2.64 |
| Bora R et al., 2014 | 0.896 | [0.868, 0.924] | 2.65 |
| Gogoi I et al., 2016 | 0.731 | [0.680, 0.782] | 2.63 |
| Nair M et al., 2016 | 0.350 | [0.321, 0.379] | 2.65 |
| **Pooled estimate 0.729 [0.466, 0.992]** | | | |
| **Group: Chhattisgarh** | | | |
| Corrêa G et al., 2017 | 0.924 | [0.902, 0.946] | 2.66 |
| **Pooled estimate 0.924 [0.902, 0.946]** | | | |
| **Group: Delhi** | | | |
| Sharma JB et al., 2003 | 0.960 | [0.949, 0.971] | 2.66 |
| **Pooled estimate 0.960 [0.949, 0.971]** | | | |
| **Group: Gujarat** | | | |
| Bala DV et al., 2012 | 0.698 | [0.619, 0.777] | 2.58 |
| **Pooled estimate 0.698 [0.619, 0.777]** | | | |
| **Group: Haryana** | | | |
| Agarwal KN et al., 2006 | 0.91 | [0.869, 0.951] | 2.64 |
| Grover K et al., 2020 | 0.853 | [0.819, 0.887] | 2.65 |
| Mangla M et al., 2016 | 0.98 | [0.971, 0.989] | 2.66 |
| **Pooled estimate 0.916 [0.842, 0.99]** | | | |
| **Group: Himachal Pradesh** | | | |
| Agarwal KN et al., 2006 | 0.681 | [0.587, 0.775] | 2.55 |
| **Pooled estimate 0.681 [0.587, 0.775]** | | | |
| **Group: Jharkhand** | | | |
| Kumar V.et al., 2014 | 0.664 | [0.588, 0.74] | 2.59 |
| **Pooled estimate 0.664 [0.588, 0.74]** | | | |
| **Group: Karnataka** | | | |
| Bone JN et al., 2022 | 0.885 | [0.879, 0.891] | 2.66 |
| Finkelstein JL et al., 2020(a) | 0.300 | [0.253, 0.347] | 2.63 |
| Krupp K et al., 2018 | 0.669 | [0.646, 0.692] | 2.66 |
| Samuel TM et al., 2013 | 0.303 | [0.256, 0.350] | 2.63 |
| Suryanarayana R et al., 2017 | 0.623 | [0.578, 0.668] | 2.64 |
| Vindhya J et al., 2019 | 0.339 | [0.284, 0.394] | 2.62 |
| **Pooled estimate 0.521 [0.326, 0.715]** | | | |
| **Group: Kerala** | | | |
| Agarwal KN et al., 2006 | 0.578 | [0.516, 0.64] | 2.61 |
| **theta** | **0.578** | **[0.516, 0.64]** |  |
| **Group: Madhya Pradesh** |  |  |  |
| Agarwal KN et al., 2006 | 0.968 | [0.937, 0.999] | 2.65 |
| Mahashabde P et al., 2014 | 0.630 | [0.575, 0.685] | 2.62 |
| **Pooled estimate 0.800 [0.469, 1.131]** | | | |
| **Group: Maharashtra** | | | |
| Ahmad N et al., 2010 | 0.748 | [0.700, 0.797] | 2.63 |
| Arlappa N et al., 2014(c) | 0.759 | [0.716, 0.802] | 2.64 |
| Patel A et al., 2018 | 0.900 | [0.898, 0.902] | 2.66 |
| **Pooled estimate 0.805 [0.707, 0.903]** | | | |
| **Group: Nagaland** | | | |
| Meshram II et al., 2020(a) | 0.522 | [0.388, 0.656] | 2.43 |
| **Pooled estimate 0.522 [0.388, 0.656]** | | | |
| **Group: Tamil Nadu** | | | |
| Agarwal KN et al., 2006(Tamil Nadu) | 0.915 | [0.876, 0.954] | 2.64 |
| Rajaratnam J et al., 2000 | 0.363 | [0.331, 0.395] | 2.65 |
| **Pooled estimate 0.639 [0.098, 1.18]** | | | |
| **Group: Telangana** | | | |
| Corrêa G et al., 2017(Telangana) | 0.924 | [0.902, 0.946] | 2.66 |
| Siddiqui R et al., 2014 | 0.932 | [0.901, 0.963] | 2.65 |
| **Pooled estimate 0.927 [0.909, 0.944]** | | | |
| **Group: Tripura** | | | |
| Debnath A et al., 2021 | 0.600 | [0.532, 0.668] | 2.6 |
| **Pooled estimate 0.600 [0.532, 0.668]** | | | |
| **Group: Uttar Pradesh** | | | |
| Singh P et al., 2015 | 0.583 | [0.527, 0.639] | 2.62 |
| **Pooled estimate 0.583 [0.527, 0.639]** | | | |
| **Group: Uttarakhand** | | | |
| Kishore S et al., 2020 | 0.335 | [0.263, 0.407] | 2.59 |
| **Pooled estimate 0.335 [0.263, 0.407]** | | | |
| **Group: Orissa** | | | |
| Agarwal KN et al., 2006(Orissa) | 0.970 | [0.944, 0.996] | 2.66 |
| Bhatia V et al., 2020 | 0.690 | [0.658, 0.722] | 2.65 |
| **Pooled estimate 0.830 [0.556, 1.105]** | | | |
| **Group: West Bengal** | | | |
| Sinha A et al., 2021 | 0.900 | [0.858, 0.942] | 2.64 |
| **Pooled estimate 0.900 [0.858, 0.942]** | | | |
| **Overall Pooled estimate 0.724 [0.653, 0.795]** | | | |
| Heterogeneity summary  Test of group differences: Qb = chi2(20) = 1267.29 Prob > Qb = 0.000 | | | |

**Supplementary Figure 21: Forest plot of pooled prevalence of anemia- among antenatal women**

### Supplementary Figure 22: Forest plot of pooled prevalence of anemia- among lactating women

### Supplementary Figure 23: Forest plot of pooled prevalence of anemia- among postnatal women

## Supplementary Figure 24: Forest plot of pooled prevalence of anemia- among delivering women

## Supplementary Figure 25: Forest plot of pooled prevalence of anemia- among medical students

**Supplementary Figure 26: Forest plot of pooled prevalence of severe anemia- among toddlers (under 3 years)**

**Supplementary Figure 27: Forest plot of prevalence of severe anemia among pre-school children (3-5 years)**

**Supplementary Figure 28: Forest plot of prevalence of severe anemia among among school children (6-18 years)**

**Supplementary Figure 29: Forest plot of prevalence of severe anemia among young and middle-aged adults (19-59 years)**

**Supplementary Figure 30: Forest plot of prevalence of severe anemia among elderly (≥60 years)**

**Supplementary Figure 31: Forest plot of prevalence of severe anemia among pregnant women**

**Supplementary Figure 32: Funnel plot for prevalence of anemia among toddlers (under 3 years)**

### Supplementary Figure 33: Funnel plot for prevalence of anemia among pre-school children (3-5 years)

**Supplementary Figure 34: Funnel plot for prevalence of anemia among school children (6-18 years)**

### Supplementary Figure 35: Funnel plot for prevalence of anemia among young and middle-aged adults (19-59 years)

**Supplementary Figure 36: Funnel plot for prevalence of anemia among elderly (≥60 years)**

**Supplementary Figure 37: Funnel plot for prevalence of anemia among pregnant women**
